# Updated Meta‐Analysis of Left Bundle Branch Area Pacing Versus Right Ventricular Pacing in Conduction System Disorders: Insights From New Evidence

**DOI:** 10.1002/clc.70278

**Published:** 2026-04-09

**Authors:** Rehan Ishaque, George S. Abela, Ghazal Ishaque, Amina Akram, F. N. U. Reya, Furqan Tarique Memon, Hamza Danish, Amna Ikram, Zulfiqar Qutrio Baloch, Supratik Rayamajhi

**Affiliations:** ^1^ Internal Medicine Liaquat University of Medical Health & Sciences Jamshoro Pakistan; ^2^ Michigan State University Lansing Michigan USA; ^3^ Shaheed Mohtarma Benazir Bhutto Medical College Karachi Pakistan; ^4^ Mayo Clinic Rochester Rochester Minnesota USA; ^5^ Ghulam Muhammad Mahar Medical College Sukkur Pakistan; ^6^ FFCA South High School Calgary Alberta Canada; ^7^ Fatima Jinnah Medical University Lahore Pakistan; ^8^ St. Francis Hospital Bartlett Bartlett Tennessee USA

## Abstract

**Introduction:**

Right ventricular pacing (RVP) has long been the standard therapy for bradyarrhythmias but may induce ventricular dyssynchrony and adverse cardiac remodeling. Physiologic pacing strategies that preserve the native conduction system, particularly left bundle branch area pacing (LBBAP), have emerged as promising alternatives. This study aimed to evaluate the comparative efficacy and safety of LBBAP versus RVP through an updated meta‐analysis.

**Methods:**

Following PRISMA guidelines, we systematically searched PubMed, Cochrane CENTRAL, and ClinicalTrials.gov for relevant studies published through June 2025. Studies comparing LBBAP and RVP in patients undergoing pacemaker implantation were included. Pooled estimates were calculated using random‐effects models.

**Results:**

A total of 40 studies comprising 8290 patients were included. LBBAP was associated with significantly shorter QRS duration compared with RVP (30 studies, *n* = 5510; MD −35.56 ms, 95% CI −41.88 to −29.24; *p* < 0.0001). Structural remodeling also favored LBBAP, with greater improvement in left ventricular ejection fraction (16 studies, *n* = 1693; MD +3.77%, 95% CI 2.43–5.12; *p* < 0.0001) and greater reduction in left ventricular end‐diastolic diameter (13 studies, *n* = 1666; MD −2.33 mm, 95% CI −3.59 to −1.07; *p* < 0.0001). Clinically, LBBAP was associated with lower heart failure hospitalization (RR 0.38, 95% CI 0.29–0.52; *p* < 0.0001) and reduced all‐cause mortality (RR 0.55, 95% CI 0.41–0.72; *p* < 0.0001), along with greater reduction in NT‐proBNP levels.

**Conclusion:**

LBBAP provides superior electrical synchrony, improved cardiac remodeling, and favorable clinical outcomes compared with RVP, while maintaining a comparable procedural safety profile.

## Graphical abstract

In this meta‐analysis of 8290 patients, left bundle branch area pacing (LBBAP) showed improved cardiac remodeling and clinical outcomes, including lower heart failure hospitalization and mortality, while maintaining comparable procedural safety compared with conventional right ventricular pacing.

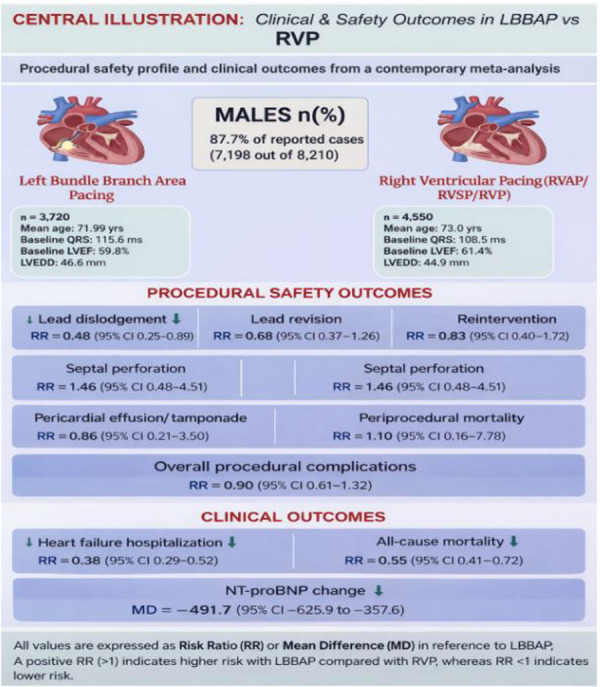

## 1 Introduction

Cardiac pacing therapy has evolved steadily since its early beginnings in the late 1950s—progressing from rudimentary devices to the very complex systems of today that aim to mirror the heart's natural conduction paths very closely [1]. Right ventricular pacing (RVP) has been a mainstay in the management of bradycardia and conduction system disease for decades [2]. Its relative longevity is explained in part by the comparative ease of the procedure, wide availability, and vast cumulative clinical experience. Yet more attention is being directed to the detriments of long‐term RVP, namely its tendency to cause ventricular dyssynchrony—a problem with a cascade of adverse effects, including left ventricular dysfunction, heart failure, atrial fibrillation (AF), and mortality [3, 4]. In an attempt to prevent these problems, physiologic pacing modes that seek to preserve or restore the heart's native conduction system are coming increasingly into the limelight [2, 5]. Among these, left bundle branch area pacing (LBBAP) has been a viable and more physiologic alternative to RVP. By targeting the distal conduction system, LBBAP minimizes electrical disruption [6]. Both when achieved through selective or non‐selective methods, LBBAP showed promising outcomes—electrical synchrony improvement, QRS narrowing, preservation of left ventricular systolic function, and reduction of pacing thresholds and lead‐related complications [7, 8].

Despite its established benefit, LBBAP is not yet universally accepted as the standard of care. The most recent European Society of Cardiology (ESC) and American Heart Association (AHA) guidelines continue to recommend RVP for the majority of clinical scenarios, particularly in individuals with less experience with LBBAP or where long‐term experience is still limited compared to RVP [9, 10]. The primary obstacle to extensive use of LBBAP as a first‐line approach stems from the lack of large randomized controlled studies and the significant heterogeneity of existing studies—including variations in design, patient populations, and clinical endpoints [11]. There is, therefore, a pressing need to synthesize emerging evidence systematically via stringent evaluations to enable clinicians to make fully informed decisions regarding the most appropriate pacing modality. In this study, we aim to provide an updated and comprehensive comparison of LBBAP and RVP by conducting a meta‐analysis of both newly published and previously reported research studies. A previous meta‐analysis by Georgios Leventopoulos established the foundation in this field [12]; our study expands on it with the addition of newer data and an updated approach. Specifically, we evaluate the change from baseline in significant outcomes—left ventricular ejection fraction (LVEF), left ventricular end‐diastolic diameter (LVEDD), and QRS duration—rather than follow‐up values alone. The aim of this review is to establish if LBBAP is clinically superior to conventional RVP in patients with conduction system disease. According to our knowledge, this is the most extensive meta‐analysis performed to date on this subject and could influence future clinical practice by fostering the broader implementation of physiological pacing modalities.

## 2 Methods

### 2.1 Data Sources and Search Strategy

The literature search for this updated systematic review and meta‐analysis was conducted in accordance with the Preferred Reporting Items for Systematic Reviews and Meta‐Analyses (PRISMA) guidelines [13]. Three electronic databases—PubMed, Cochrane Library, and ClinicalTrials.gov—were searched for studies published between November 2022 and June 2025. The search was limited to this time frame because the literature prior to November 2022 had already been comprehensively reviewed in the previously published meta‐analysis by Leventopoulos et al. [12]. We therefore used that review as a baseline and focused our updated search on identifying newly published studies from November 2022 onwards. The search strategy included a combination of MeSH terms and keywords: “left bundle branch area pacing” OR “LBBAP” AND “right ventricular pacing” OR “RVP” OR “RVAP” OR “RVSP.” The initial search yielded 772 results. All the articles were imported onto the Rayyan web version for primary and secondary screening [14].

### 2.2 Selection Criteria

**Figure 1:**
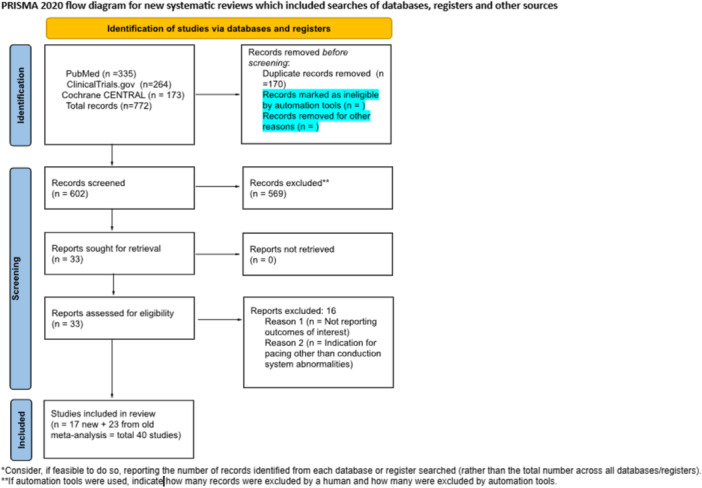
PRISMA flow diagram of study selection. The diagram shows the number of records identified, screened, assessed for eligibility, and included in the systematic review and meta‐analysis, with reasons for exclusions at each stage.

After removing 170 duplicates, the remaining 602 articles were screened independently by two authors. In primary screening, 569 non‐relevant articles (reviews, conference abstracts, case reports, and commentaries) were excluded based on their titles and abstracts. Full texts of the remaining 33 articles were then assessed for eligibility based on predetermined criteria, set after discussion and consensus between all authors. Inclusion criteria were (1): Original studies (clinical trials, retrospective, and prospective observational) (2), studies that compare LBBAP versus RVP (3), studies involving patients with a reported LVEF > 40%, and (4) studies focusing on patients with conduction system disorders as the primary indication for pacing. Exclusion criteria included (1) review articles, case reports, case series, conference abstracts, and commentaries; (2) studies that did not report any of the outcomes of interest; (3) studies involving patients with LVEF < 40%; (4) single‐arm studies without a comparator group; and (5) studies in which the pacing indication was not related to conduction system disorders (Figure 1). Studies evaluating conduction system pacing (CSP) without reporting outcomes separately for LBBAP were excluded to maintain consistency with the predefined comparison of LBBAP versus RVP.

### 2.3 Data Extraction

The data was extracted from scratch for all the studies (the newly screened plus the prior screened by the Georgios Leventopoulos et al. meta‐analysis) to address any discrepancies, if present. The data extraction was done by four reviewers, and any problems arising during the extraction were solved by mutual discussion between all the authors, and a consensus was reached. Following individual data extraction, data sheets were double‐checked for any discrepancies by redistributing the studies again. The data were collected for baseline characteristics, including the number of evaluable patients, age, proportion of males, follow‐up periods, baseline QRS duration, LVEF, LVEDD, use of ACEI/ARBs, proportion of right bundle branch block (RBBB), left bundle branch block (LBBB), hypertension, coronary artery disease (CAD), AF, and diabetes mellitus (DM). The primary outcomes included changes from baseline in QRS duration, LVEF, and LVEDD. Additional procedural and device‐related parameters extracted included pacing impedance, pacing threshold, R‐wave amplitude, procedural time, and fluoroscopy time.

In response to reviewer feedback, additional clinical and safety outcomes were extracted from all included studies. These included procedural complications (lead dislodgement, septal perforation, and pericardial effusion or tamponade), reinterventions, lead revision rates, and periprocedural mortality. Clinical endpoints, including heart failure hospitalization, AF admissions, all‐cause mortality, cardiac resynchronization therapy (CRT) upgrade, pacemaker‐induced cardiomyopathy (PMICM), and change in NT‐proBNP levels, were also collected when reported. Because follow‐up durations varied substantially across studies, binary clinical outcomes were extracted as reported in each study irrespective of follow‐up timepoint. NT‐proBNP was analyzed as a continuous outcome using the longest available follow‐up measurement reported in each study. Data on ventricular pacing burden were also extracted when reported. Ventricular pacing burden was variably reported across studies, including mean or median pacing percentage, categorical thresholds (e.g., > 80% pacing), or expected pacing burden based on pacing indication such as atrioventricular block. Due to heterogeneity in reporting formats and incomplete reporting across studies, ventricular pacing burden could not be quantitatively synthesized or used for subgroup analysis (Supporting Information S1: Table S2).

### 2.4 Risk of Bias Assessment and Quality Evaluation

The risk of bias and quality assessments were conducted appropriately according to the study designs. For randomized controlled trials, the Cochrane Risk of Bias 2.0 (RoB 2.0) tool was used [15]. For observational studies, the Newcastle–Ottawa Scale (NOS) was applied [16]. These tools are designed to identify potential sources of bias related to participant selection, classification of interventions or exposures, outcome measurement, and loss to follow‐up. Based on these assessments, all included studies were categorized as having low, moderate, or high risk of bias, as shown in Supporting Information S1: Table S1.

### 2.5 Data Analysis

To calculate the change in QRS duration from baseline, a common post‐implantation timepoint was selected across studies. This included any QRS duration reported during the immediate post‐implantation period, defined as intraoperative, perioperative, or within 7 days of follow‐up. For changes in LVEF and LVEDD from baseline, follow‐up values were extracted at the longest available follow‐up timepoint reported in each study. When multiple follow‐up measurements were available, the timepoint closest to 12 months post‐implantation was preferentially selected. In studies where only a single follow‐up measurement was reported outside this window, that timepoint was used for analysis. Consequently, the selected follow‐up durations varied across studies depending on the reporting structure of each study. To provide transparency regarding the follow‐up timepoints used for structural outcomes, a detailed table summarizing the reported follow‐up durations and the timepoints selected for the meta‐analysis across all included studies is provided in Supporting Information S1: Table S3.

For pacing impedance, pacing threshold, and R‐wave amplitude post‐implantation, intraoperative values were recorded for analysis to minimize heterogeneity. These parameters were also analyzed at the longest reported follow‐up periods, referred to as “pacing impedance and threshold at follow‐up” and “R‐wave amplitude at follow‐up,” where the longest available follow‐up values were preferentially extracted. Procedural time and fluoroscopy time were considered independent of follow‐up duration and were therefore extracted directly from baseline procedural data reported in each study.

When outcome data were reported only in graphical form, numerical values were extracted using WebPlotDigitizer [17]. For studies reporting medians with interquartile ranges or ranges instead of means and standard deviations, mean ± SD values were estimated using the method described by Wan et al. [18]. To calculate the change from baseline for continuous outcomes such as LVEF, LVEDD, and QRS duration, the mean change was calculated by subtracting the baseline mean from the follow‐up mean. The standard deviation of change from baseline was estimated using the imputation method recommended by the Cochrane Handbook, assuming a correlation coefficient of 0.5 between baseline and follow‐up measurements [19].

For binary outcomes, pooled risk ratios (RRs) with 95% confidence intervals were calculated using random‐effects models. Continuous outcomes were pooled as mean differences (MDs) with corresponding 95% confidence intervals. Statistical heterogeneity across studies was assessed using Cochran's *Q* test and quantified using the *I*² statistic. Sensitivity analyses using a leave‐one‐out (LOO) approach were performed to evaluate the robustness of pooled estimates. Publication bias was assessed using funnel plots and Egger's regression test, where applicable. To assess baseline comparability between groups, pooled estimates of baseline demographic and clinical characteristics were calculated across studies. These included age, hypertension, DM, CAD, AF, and baseline LVEF. Continuous variables were pooled as MDs, while categorical variables were pooled using RRs.

## 3 Results

### 3.1 Baseline Demographics and Transplant Characteristics

A total of 40 studies were included in this updated systematic review and meta‐analysis, of which 17 were newly identified studies [20, 21, 22, 23, 24, 25, 26, 27, 28, 29, 30, 31, 32, 33, 34, 35, 36, 37, 38, 39, 40, 41, 42, 43, 44, 45, 46, 47, 48, 49, 50, 51, 52, 53, 54, 55, 56, 57], comprising 8290 patients overall. The studies by Heckman et al. (2021) and Xie et al. (2021) were excluded because both LBBAP and RVP were assessed in the same participants during the implantation procedure, rather than in independent patient groups. Among them, 3720 patients received LBBAP and 4550 underwent RVP, excluding one study which did not fully differentiate the numbers. The overall gender distribution favored males in 87.7% of reported cases (7198 out of 8210). The weighted mean age was 71.99 years in the LBBAP group and 72.99 years in the RVP group. Reported follow‐up durations varied considerably across studies, ranging from 7 days to up to 4 years. The weighted mean baseline QRS duration was 115.55 ms in the LBBAP group and 108.48 ms in the RVP group. The baseline weighted mean LVEF was 59.75% for LBBAP and 61.43% for RVP. Additionally, the weighted mean baseline LVEDD was 46.56 mm in the LBBAP group and 44.91 mm in the RVP group.

Across 34 studies, hypertension was reported in 67.2% (*n* = 2334/3474) of LBBAP patients and 68.1% (*n* = 2800/4122) of RVP patients. Across 14 studies, the use of ACEIs/ARBs was reported in 49.8% (*n* = 922/1852) of LBBAP patients and 44.8% (*n* = 1138/2539) of RVP patients. Across 20 studies, LBBB was present in 12.4% (*n* = 232/1873) of LBBAP patients and 7.3% (*n* = 156/2125) of RVP patients, while RBBB was observed in 23.6% (*n* = 436/1844) and 19.7% (*n* = 412/2094) of LBBAP and RVP patients, respectively. Across 29 studies, AF was reported in 29.2% (*n* = 897/3069) of LBBAP patients and 27.1% (*n* = 1039/3832) of RVP patients. Across 33 studies, DM was present in 27.8% (*n* = 960/3454) of LBBAP patients and 25.8% (*n* = 1058/4102) of RVP patients. Across 28 studies, CAD was reported in 25.6% (*n* = 802/3136) of LBBAP patients and 24.7% (*n* = 865/3508) of RVP patients. Ventricular pacing burden was reported in a limited subset of studies (Supporting Information S1: Table S2). Among studies reporting quantitative values, the mean pacing burden was generally high in both groups, frequently exceeding 80%–90%, reflecting the predominance of atrioventricular conduction disorders as the pacing indication. However, reporting formats varied substantially (mean percentage, categorical thresholds, or expected pacing dependency), which precluded formal meta‐analysis or subgroup analysis based on pacing burden. Baseline demographic and clinical characteristics were broadly comparable between the LBBAP and RVP groups across the included studies. The pooled analysis demonstrated a slightly lower mean age in the LBBAP group compared with the RVP group (MD −0.97 years, 95% CI −1.49 to −0.44; *p* < 0.001). Baseline LVEF was also marginally lower in the LBBAP group (MD −0.70%, 95% CI −1.32 to −0.09; *p* = 0.02). However, there were no significant differences between groups in the prevalence of AF, hypertension, DM, or CAD. These findings suggest that baseline clinical characteristics were largely comparable between groups (Supporting Information S1: Table S4).

The baseline characteristics of the included studies are summarized in Table 1. A more detailed presentation of baseline characteristics for each included study, including the number and percentage of comorbidities (hypertension, LBBB, RBBB, DM, use of ACE inhibitors/ARBs, and CAD), is provided in Supporting Information S1: Section S6.

**Table 1:** Baseline characteristics of the included studies.

Baseline characteristics table of all the included studies with demographics and clinical characteristics											
**Author**	**Year of publication**	**Sample sizes**		**Study design**	**Baseline QRS duration (mean SD)**		**Baseline LVEF (mean SD)**		**Age (mean ± SD)**		**Follow‐up**
		**LBBAP**	**RVP**		**LBBP**	**RVP**	**LBBP**	**RVP**	**LBBAP**	**RVP**	
Wang X	2024	120	117	Observational study	111 ± 24	114 ± 21	57 ± 13	56 ± 13	74.3 ± 6.6	74.7 ± 6.5	48.5 (34.9–60) months
Zhao	2023	36	36	RCT	NR	NR	62.27 ± 7.23	63.18 ± 5.80	64.26 ± 14.10	68.11 ± 10.04	6 months
Zhang	2024	43	43	Observational study	116.3 ± 26.2	113.1 ± 27.2	62.2 ± 2.5	63.3 ± 5.2	75.0 ± 10.6	72.4 ± 10.0	14.1 ± 7.5 months
Yao	2025	30	30	Observational study	126.10 ± 30.92	128.61 ± 26.94	64.60 ± 6.84	65.23 ± 3.74	70.43 ± 12.68	74.00 ± 9.58	12 months
Liu X et al.	2022	33	21	Observational study	113.58 ± 21.22	113.48 ± 21.80	64.97 ± 6.15	63.57 ± 9.98	73.67 ± 11.87	68.14 ± 11.66	13.80 ± 4.47 months
Li Q et al.	2021	42	42	RCT	109.48 ± 25.58	97.36 ± 22.20	NR	NR	65.36 ± 13.08	68.19 ± 9.52	7 days
Liu et al.	2022	45	46	Observational study	109.09 ± 19.88	106.04 ± 20.78	65 (61.5−70)	67 (61−69.3)	74 ± 9.2	70.5 ± 11.7	LBBAP = 14 ± 6.1, RVSP = 13.3 ± 6.1
Li X et al.	2021	235	120	Observational study	119.5 ± 26.7	117.9 ± 27.9	61.7 ± 7.4	61.5 ± 6.4	63.3 ± 15.5	62.1 ± 17.2	11.4 ± 2.7 months
Li W	2022	30	38	Observational study	122.67 ± 33.19	RVAP = 110.39 ± 25.86	56.40 ± 14.26	RVAP = 61.68 ± 7.62	70.23 ± 9.58	RVAP = 73.13 ± 6.62	12 months
Das et al.	2020	22	28	RCT	131.64 ± /17.80	132.73 ± /16.71	61.15 ± 4.04	62.50 ± 4.00	63.36 ± 7.82	61.64 ± 5.90	6 months
Chen et al.	2020	237	317	Observational study	117.09 ± 25.82	105.04 ± 12.18	NR	NR	67.76 ± 13.29	69.15 ± 11.48	18.13 ± 1.77 months for LBBP, 18.37 ± 2.13 months for RVP
Chen et al.	2022	Total 20	NR	Observational study	118.75 ± 24.63	118.75 ± 24.63	62.12 ± 13.83	62.12 ± 13.83	66.15 ± 13.65	66.15 ± 13.65	18 months
Chen et al.	2018	20	10	Observational study	110.00 ± 33.38	108.70 ± 26.26	60.00 ± 10.60	60.70 ± 6.08	66.90 ± 7.49	71.65 ± 7.80	3 months
Cai B. et al.	2020	20	17	Observational study	NR	NR	53 ± 11	58 ± 9	84 ± 6	83 ± 7	
Byeon K	2022	42	84	Observational study	NR	NR	62 ± 9	63 ± 7	71 ± 16	69 ± 15	6.8 ± 4.8 months
Okubo 2025	2025	81	79	Observational study	113.7 ± 26.3	107.8 ± 26.1	58.8 ± 9.5	59.1 ± 8.7	76.9 ± 11.7	77.4 ± 10.3	12 months
Palmisano	2023	73	201	Observational study	NR	NR	55.5 ± 9.0	56.7 6 9.3	79.2 ± 9.8	79.3 6 ± 10.0	18 months
Ramalingam et al.	2024	50	50	Observational study	113.8 ± 24.1	110.6 ± 26.5	61.7 ± 5.8	60.6 ± 6.5	63 (14)	64 (13)	6 months
Wang Q	2024	109	158	Observational study	108.1 ± 23.7	96.2 ± 21.8	61.3 ± 6.3	62.5 ± 4.3	80.7 ± 4.1	80.8 ± 4.0	LBBAP = 35.2 ± 15.6 months)
RVP = 28.0 ± 17.1											
Chen	2023	393	510	Observational study	NR	NR	65.0 ± 8.15%	65.5 ± 7.41%	71.7 ± 11.7 years	73.0 ± 13.2 years	4 years
Chen	2025	122	166	Observational study	105.8 ± 30.8 ms	104.2 ± 27.0 ms	62.5 ± 7.2%	63.7 ± 5.1%	64.5 ± 13.2	67.3 ± 11.7	24 ± 6 months
Dell'Era	2024	20	18	Observational study	NR	NR	53 ± 11%	58 ± 9%	83 ± 7 years	84 ± 6 years	4.2 ± 2.8 months
Kono	2025	75	296	Observational study	124 ± 36.3 ms	104 ± 37.1 ms	64.7 ± 4.8%	63.3 ± 4.4%	78.0 ± 11.1 years	78.0 ± 8.9 years	2.9 years (IQR 2.0−3.6 years)
Lee‐Ky	2025	243	495	Observational study	121.1 ± 32.4 ms	114.0 ± 28.8 ms	61.2 ± 7.0%	61.2 ± 7.0%	71.5 ± 13.4	72.5 ± 11.6	1 year median follow‐up
Mao	2024	31	29	Observational study	107 ± 24 ms	114 ± 22 ms	65 ± 6%	65 ± 7%	71 ± 10 years	75 ± 9 years	15 ± 9 months
Mao Y	2023	45	33	Observational study	106.89 ± 27.1 ms	88.6 ± 12.6 ms	55.17 ± 17.25%	67.79 ± 5.08%	72.7 ± 12.2	72.9 ± 11.8	1 year
Maqueda	2024	100	100	Observational study	126.17 ± 28.3 ms	130.21 ± 29.1 ms	62.3 ± 7.1%	60.8 ± 6.6%	77.3 ± 8.0 years	78.7 ± 6.7 years	6 months
Zhang S	2021	29	37	Observational study	NR	NR	55.08 ± 4.32%	56.29 ± 5.40%	63.60 ± 8.80	67.40 ± 8.81	2 year max follow up, 12−24 months
Zhu H	2021	406	313	Observational study	112.4 ± 24.1 ms	98.0 ± 18.3 ms	61.2 ± 7.27%	62.5 ± 4.14%	64.9 ± 14.3	67.5 ± 12.2	13.6 ± 7.8 months
Zhu H	2023	257	270	Observational study	111.8 ± 25.5 ms	99.8 ± 20.0 ms	62.8 ± 4.9%	63.1 ± 5.4%	63.6 ± 13.5	66.9 ± 11.5	11.1 months
Miyajima et al.	2022	39	42	Observational study	108 ± 25	107 ± 25	65 ± 6.6	63 ± 10	78 ± 10	79 ± 11	3 months
Niu HX et al.	2021	20	30	Observational study	133.8 ± 32.9	134.9 ± 30.6	51.9 ± 8.5	52.3 ± 9.3	NR	NR	15.0 ± 9.1 months
Riano Ondiviela et al.	2021	60	60	RCT	121.6 ± 29.6	109.9 ± 25.8	NR	NR	76.7 ± 9	79.7 ± 8	3 months
Sharma et al.	2022	321	382	Observational study	117.09 ± 25.82	105.04 ± 12.18	NR	NR	75.33 ± 12.26	74.96 ± 11.85	18 months
Okubo et al.	2022	43	46	Observational study	108.2 ± 20.3	104.8 ± 22.7	58.6 ± 7.9	59.9 ± 9.5	77.4 ± 10.6	76.2 ± 10.9	6 months
Sun Z	2020	16	16	Observational study	106.25 ± 25.00	107.50 ± 28.17	NR	NR	71.4 ± 14.4	73.6 ± 8.9	12 months
Wang JF et al.	2019	66	65	RCT	99.24 ± 13.60	101.88 ± 12.21	61.3 ± 5.7	62.1 ± 6.3	71.12 ± 13.14	72.03 ± 12.11	6 months
Wang Z et al.	2021	52	44	Observational study	110.0 ± 19.9	104.3 ± 20.3	60.1 ± 6.4	61.5 ± 4.7	67.9 ± 12.6	67.2 ± 11.6	13.9 ± 7.0 months
Yao L et al.	2022	25	25	RCT	NR	NR	NR	NR	(66.3 ± 11.0	(69.2 ± 12.8	18 months
Zhang JM	2019	23	21	Observational study	131.83 ± 41.68	93.62 ± 8.28	45.75 ± 18.47	65.93 ± 4.16	64.61 ± 12.65	65.76 ± 13.53	12 months

### 3.2 Change in QRS Duration From Baseline

**Figure 2:**
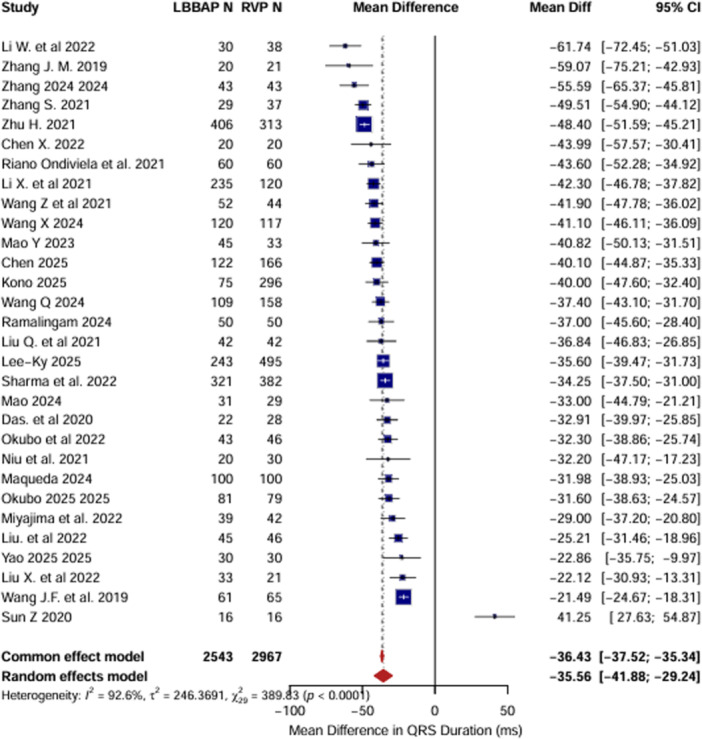
Forest plot representing the mean difference in QRS duration from baseline to post‐implantation between left bundle branch area pacing and right ventricular pacing. Negative values indicate a greater reduction in QRS duration with LBBAP compared to RVP.

The pooled analysis for QRS duration included 30 studies with a total of 5510 patients (LBBAP: 2543, RVP: 2967). The results indicated QRS duration was significantly lower in the LBBAP group compared to the RVP group (MD: –35.56 ms [95% CI: –41.88 to –29.24], *I*² = 92.6%, *τ*² = 246.37, *p* < 0.0001), favoring LBBAP (Figure 2). Heterogeneity analysis across 30 studies confirmed robustness of results (MD range: −37.77 to −34.71 ms), with Sun Z (2020) and Li W et al. (2022) showing the highest influence on the pooled QRS duration MD. Egger's test showed no significant publication bias, and the funnel plot appeared symmetric upon visual inspection.

### 3.3 Change in LVEF From Baseline

**Figure 3:**
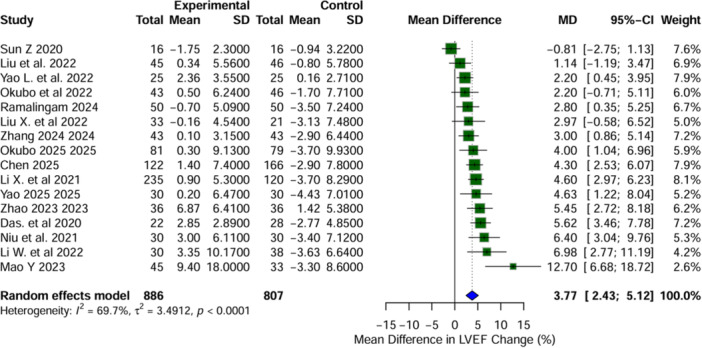
Forest plot representing the mean difference in left ventricular ejection fraction (LVEF) from baseline to post‐implantation between left bundle branch area pacing and right ventricular pacing. Positive values indicate an increase in LVEF with LBBAP compared to RVP.

This meta‐analysis encompassed 16 studies, comprising a total of 1693 patients (LBBAP: 886, RVP: 807). The change in LVEF was notably more pronounced in the LBBAP group compared to the RVP group, with an MD of +3.77% (95% CI: 2.43−5.12, *I*² = 69.7%, *p* < 0.0001), indicating a preference for LBBAP (Figure 3). The heterogeneity analysis corroborated the robustness of LVEF enhancement with LBBAP (MD range: from 3.51 to 4.02), with heterogeneity (*I*²) spanning from 52.0% to 71.7%. Sun Z (2020) and Mao Y (2023) exerted the most substantial influence on the pooled estimates. Egger's test did not reveal significant publication bias (*p* = 0.0946), and the funnel plot appeared symmetric upon visual inspection.

### 3.4 Change in LVEDD From Baseline

**Figure 4:**
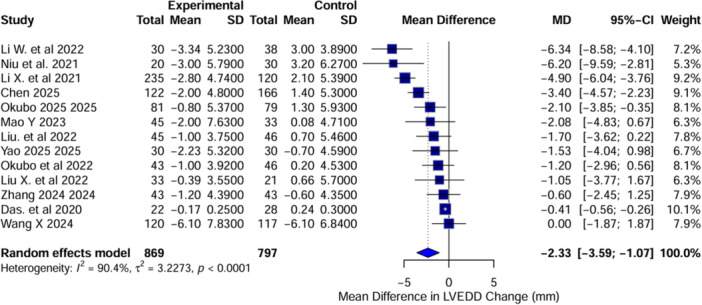
Forest plot representing the mean difference in left ventricular end‐diastolic diameter from baseline to post‐implantation between left bundle branch area pacing and right ventricular pacing. Negative values indicate a greater reduction in LVEDD with LBBAP compared to RVP.

A total of 13 studies were included in this analysis, comprising 1666 patients (LBBAP: 869, RVP: 797). The LVEDD was significantly reduced from baseline in the LBBAP group compared to RVP, with an MD of –2.33 mm (95% CI: –3.59 to –1.07, *I*² = 90.4%, *p* < 0.0001) (Figure 4). The heterogeneity analysis further supported the consistency of these findings, with MD values ranging from –2.55 to –2.01 mm, and heterogeneity (*I*²) ranging from 77.8% to 91.2%. Li W et al. (2022) and Das et al. (2020) were by far the most influential studies, contributing the largest shifts in the pooled estimate. Egger's test reported a significant publication bias (*p* = 0.0132), further supported by asymmetry observed in the funnel plot. These findings suggest that LBBAP is associated with a significantly greater reduction in LVEDD from baseline compared to RVP.

### 3.5 Pacing Impedance

Pacing impedance at the time of implantation reported no statistically significant difference between the groups (MD = –15.27 ohms [95% CI: –34.86 to 4.31], *I*² = 65.5%, *τ*² = 1255.76, *p* < 0.0001). This analysis included 26 studies with a total of 4103 patients (LBBAP: 1753; RVP: 2350). The heterogeneity analysis confirmed that no single study shifted the results to statistical significance. Also, Egger's test (*p* = 0.0077) suggested potential publication bias. Similarly, at the time of follow‐up, no significant difference was found between the groups (MD = –18.93 ohms [95% CI: –41.06 to 3.20], *I*² = 95.5%, *τ*² = 2517.18, *p* < 0.0001). Therefore, 25 studies involving 4899 patients (LBBAP: 2158; RVP: 2741) were included in this analysis. The heterogeneity analysis again showed no influential studies, and Egger's test (*p* = 0.8164) indicated no publication bias.

### 3.6 Pacing Threshold

The pooled analysis for pacing threshold at the time of implantation included 30 studies with a total of 5360 patients (LBBAP: 2435; RVP: 2925), showing no significant difference between LBBAP and RVP (MD = +0.01 V [95% CI: –0.04 to +0.07], *I*² = 91.8%, *p* < 0.0001). Similarly, the pacing threshold at follow‐up reported no statistically significant difference between the groups (MD = +0.04 V [95% CI: –0.00 to +0.08], *I*² = 80.2%, *p* < 0.0001), including 24 studies with a total sample size of 4636 patients (LBBAP: 2078; RVP: 2558). Heterogeneity analysis for both did not substantially change the results, and Egger's test revealed no significant publication bias.

### 3.7 R Wave Amplitude

The R wave amplitude at the time of implantation was significantly higher in the LBBAP group compared to RVP (MD = +1.03 mV [95% CI: 0.26−1.80], *I*² = 76.8%, *τ*² = 2.12, *p* < 0.0001). The analysis included 24 studies with a total of 5275 patients (LBBAP: 2373; RVP: 2902). Similarly, the pooled analysis of R wave amplitude at follow‐up included 21 studies with a total of 5025 patients (LBBAP: 2183; RVP: 2842), demonstrated a significantly higher R wave amplitude in the LBBAP group (MD = +2.85 mV [95% CI: 1.35−3.56], *I*² = 95.8%, *τ*² = 5.27, *p* < 0.0001). Heterogeneity analyses confirmed the robustness of the results, with no single study significantly impacting the statistical significance. Egger's test showed no significant publication bias (*p* = 0.0992).

### 3.8 Procedural Time and Fluoroscopic Time

For the analysis of procedural time, the data came from 15 studies with a total of 3913 patients (LBBAP: 1649; RVP: 2264); While for the fluoroscopic time outcome, data came from 17 studies with 4720 patients (LBBAP: 1982; RVP: 2738). Procedural time was significantly longer in the LBBAP group compared to RVP (MD = +16.37 min [95% CI: 10.58−22.16], *I*² = 95.0%, *p* < 0.0001). Similarly, fluoroscopic time was also significantly longer in the LBBAP group compared to RVP (MD = +3.13 min [95% CI: 1.42−4.83], *I*² = 96.3%, *p* < 0.0001). Heterogeneity analyses confirmed the robustness of the results for both outcomes. Procedural time showed pooled MDs ranging from 15.44 to 17.44 min with significant publication bias (*p* = 0.0005), while fluoroscopic time showed pooled MDs of 2.73–3.42 min without evidence of bias (*p* = 0.5483).

### 3.9 Procedural Safety Outcomes

Procedural safety outcomes were reported across multiple studies, including lead dislodgement, lead revision rates, reintervention, septal perforation, pericardial effusion or cardiac tamponade, periprocedural mortality, and overall procedural complications. Lead dislodgement was reported in 15 studies and occurred significantly less frequently in the LBBAP group compared with the RVP group (RR = 0.48, 95% CI 0.25–0.89; *I*² = 0%, *p* = 0.021). Lead revision rates were reported in seven studies and were comparable between the two groups (RR = 0.68, 95% CI 0.37–1.26; *I*² = 0%, *p* = 0.2247). Similarly, the pooled analysis demonstrated no significant differences in overall procedural complications between LBBAP and RVP (RR = 0.90, 95% CI 0.61–1.32; *I*² = 0%, *p* = 0.5764).

Pericardial effusion or cardiac tamponade was reported in seven studies and showed no statistically significant difference between LBBAP and RVP (RR = 0.86, 95% CI 0.21–3.50; *I*² = 0%, *p* = 0.8351). Periprocedural mortality was rare and did not differ significantly between the groups (RR = 1.10, 95% CI 0.16–7.78; *I*² = 0%, *p* = 0.9204). Reintervention rates were also similar between the two pacing strategies (RR = 0.83, 95% CI 0.40–1.72; *I*² = 0%, *p* = 0.6117). Finally, septal perforation showed no significant difference between LBBAP and RVP (RR = 1.46, 95% CI 0.48–4.51; *I*² = 0%, *p* = 0.5061).

### 3.10 Clinical Outcomes

Clinical outcomes, including heart failure hospitalization, all‐cause mortality, and change in NT‐proBNP, were evaluated across the included studies. Heart failure hospitalizations were reported in 12 studies and demonstrated a significantly lower incidence in the LBBAP group compared with the RVP group (RR = 0.38, 95% CI 0.29–0.52; *I*² = 0%, *p* < 0.0001), indicating a substantial reduction in heart failure admissions associated with LBBAP. All‐cause mortality was also reported in 12 studies and was significantly lower in patients receiving LBBAP compared with RVP (RR = 0.55, 95% CI 0.41–0.72; *I*² = 0%, *p* < 0.0001). Similarly, pooled analysis of NT‐proBNP change from three studies demonstrated a greater reduction in NT‐proBNP levels in the LBBAP group compared with RVP (MD = −491.7, 95% CI −625.9 to −357.6; *I*² = 0%, *p* < 0.0001), suggesting improved cardiac remodeling and reduced neurohormonal activation with LBBAP. Overall, these findings indicate that LBBAP is associated with improved clinical outcomes, including reduced heart failure hospitalizations, lower all‐cause mortality, and greater improvement in NT‐proBNP levels compared with conventional RVP. Other clinical outcomes, such as AF‐related admissions, cardiac CRT upgrade, and PMICM, were reported in only a limited number of studies (typically one to two studies each). Due to the small number of available studies and inconsistent reporting formats, these outcomes could not be pooled quantitatively and were therefore not included in the meta‐analysis.

**Figure 5:**
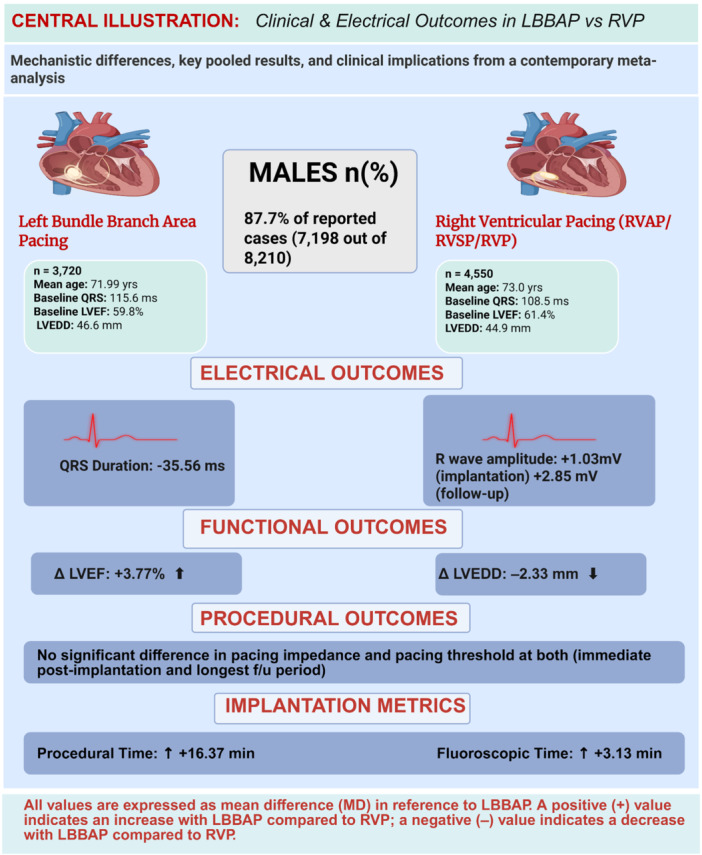
Central illustration summarizing the electrical, functional, and procedural outcomes comparing left bundle branch area pacing (LBBAP) with right ventricular pacing (RVP). The figure highlights pooled estimates for electrical parameters (QRS duration and R‐wave amplitude), structural cardiac remodeling outcomes (change in LVEF and LVEDD), and procedural metrics including pacing impedance, pacing threshold, procedural time, and fluoroscopic time. Effect estimates are expressed as mean differences (MD). Positive values indicate an increase with LBBAP relative to RVP, while negative values indicate a decrease.

**Figure 6:**
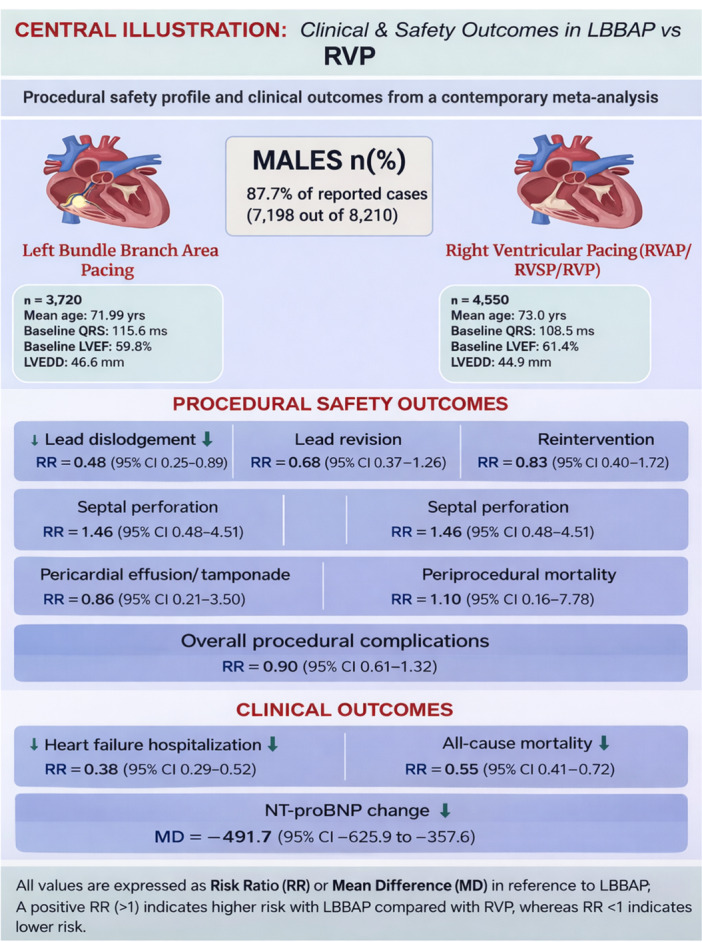
Central illustration summarizing the procedural safety profile and clinical outcomes comparing left bundle branch area pacing (LBBAP) with right ventricular pacing (RVP). The figure presents pooled risk estimates for procedural complications (lead dislodgement, lead revision, reintervention, septal perforation, pericardial effusion or tamponade, periprocedural mortality, and overall complications) as well as major clinical outcomes including heart failure hospitalization, all‐cause mortality, and change in NT‐proBNP levels. Effect estimates are expressed as risk ratios (RR) or mean differences (MD) with corresponding 95% confidence intervals.

Figures 5 and 6 present the central illustrations summarizing the pooled effect estimates from the meta‐analysis comparing LBBAP versus RVP. Figure 5 summarizes electrical, functional, and procedural outcomes, whereas Figure 6 summarizes procedural safety and clinical outcomes.

All the remaining forest plots, sensitivity analyses, funnel plots, and Egger's test are present detailed in Supporting Information S1: S5.

## 4 Discussions

To summarize, our meta‐analysis yielded several clinically important findings. LBBAP was associated with a significant reduction in QRS duration and LVEDD, as well as an improvement in LVEF from baseline after pacemaker implantation. Although no significant differences were observed in pacing impedance and pacing threshold, LBBAP was associated with a significantly higher R wave amplitude and longer procedural and fluoroscopic times compared to RVP.

While prior meta‐analyses—such as those by Ahsan et al. (focusing on patients with atrioventricular block) and Georgios Leventopoulos et al. (assessing broader indications)—have compared LBBAP and RVP, none have comprehensively evaluated the change from baseline in QRS duration, LVEF, and LVEDD at follow‐up [6, 12]. Notably, Ahsan et al. did attempt to calculate the change in LVEF; however, the method employed involved the simple subtraction of standard deviations from pre‐ and post‐intervention values, which is statistically invalid. In contrast, our study followed Cochrane‐recommended methodology for calculating change scores, thereby ensuring methodological rigor [19]. Compared to the meta‐analysis by Peng et al. [58], which broadly evaluated physiologic pacing versus RVP, our study focused exclusively on LBBAP versus RVP, offering a more targeted and homogeneous comparison. While Peng et al. reported a significantly lower pacing threshold with RVP compared to physiologic pacing overall, our analysis—focusing specifically on LBBAP versus RVP—did not find a statistically significant difference in pacing thresholds at implantation or follow‐up. This discrepancy may reflect Peng et al.'s inclusion of His‐bundle pacing (HBP), which is known to require higher pacing outputs, whereas our study provides a more focused and homogeneous comparison of LBBAP alone. Furthermore, previous meta‐analyses often lacked detailed reporting of baseline characteristics, which limits the interpretability and applicability of their findings. Our study addresses this gap by providing a comprehensive synthesis of patient demographics and clinical variables—including hypertension, DM, AF, bundle branch blocks, CAD, and ACEI/ARB usage—across the included studies. This enhances the contextual relevance of our results and enables more accurate comparisons between the two pacing strategies. Importantly, our meta‐analysis represents the most extensive evidence base to date, incorporating 40 studies and a total of 8290 patients. This large sample size strengthens the statistical power of our findings and increases their generalizability across diverse patient populations.

In addition to its large sample size and comprehensive baseline characterization, our study applied robust methods to assess the validity of the results. For each reported outcome, we performed funnel plot analyses and Egger's regression tests to evaluate potential publication bias [59]. Furthermore, LOO sensitivity analyses were conducted across all outcomes to assess the influence of individual studies on the pooled estimates, thereby strengthening the reliability and consistency of our findings [60].

The clinical relevance of pacing modality is closely related to ventricular pacing burden, as higher percentages of RVP exposure are known to increase the risk of pacing‐induced cardiomyopathy. In many of the included studies, patients underwent pacemaker implantation for atrioventricular conduction disorders and were therefore expected to require a high ventricular pacing burden. However, ventricular pacing burden was inconsistently reported across studies, and the reporting format varied widely (mean pacing percentage, median pacing percentage, or categorical thresholds). As a result, formal subgroup analysis based on pacing burden was not feasible. Nevertheless, the observed improvements in electrical synchrony and favorable structural remodeling with LBBAP suggest that physiologic pacing may mitigate the deleterious effects associated with high RVP exposure.

Despite its strengths, this meta‐analysis has several limitations. Most of the included studies were observational in design, with only a few randomized controlled trials, introducing potential biases inherent to non‐randomized data. The follow‐up durations and patient selection criteria varied across studies, which may contribute to heterogeneity in outcome estimates. For outcomes such as pacing impedance, threshold, and R wave amplitude, we extracted values from the longest available follow‐up time in each study, which may have introduced temporal variability and additional heterogeneity. Although baseline comorbidities—including hypertension, DM, AF, CAD, and conduction disorders—were thoroughly analyzed and reported, we did not perform subgroup analyses or meta‐regression. This decision was primarily due to the lack of uniformly stratified outcome data across studies and the limitations of aggregate (study‐level) data. Many studies did not report outcomes separately by comorbidity status or other relevant subgroups, precluding statistically valid subgroup or moderator analyses. Similarly, several clinically relevant outcomes, including AF admissions, CRT upgrade, and PMICM, were sparsely reported across the included studies, which limited the ability to perform quantitative pooled analyses for these endpoints. In addition, most included studies enrolled patients with preserved baseline left ventricular systolic function undergoing pacemaker implantation for bradyarrhythmia, consistent with our inclusion criteria (LVEF > 40%). Therefore, subgroup analyses comparing preserved versus reduced baseline LVEF were not feasible. Future studies evaluating conduction system pacing in patients with reduced LVEF may help clarify whether the benefits of LBBAP extend to populations with pre‐existing ventricular dysfunction.

## Author Contributions

Rehan Ishaque and George S. Abela conceptualized the study. Ghazal Ishaque and Amina Akram performed literature screening and data extraction. Hamza Danish contributed to the extraction of additional clinical outcome data and statistical pooling during the revision process. Furqan Tarique Memon performed statistical analysis. Amna Ikram drafted the manuscript. All authors critically reviewed the manuscript and approved the final version.

## AI Use Disclosure

ChatGPT (OpenAI, San Francisco, CA, USA) was used solely for language editing and improving text clarity. No part of the scientific content, analysis, or conclusions was generated by artificial intelligence. All content was written, reviewed, and approved by the authors, who take full responsibility for the final manuscript.

## Funding

The authors received no specific funding for this work.

## Ethics Statement

Ethical approval was not required for this study, as it is a systematic review and meta‐analysis of previously published studies using de‐identified data.

## Conflicts of Interest

The authors declare no conflicts of interest.

## Supporting information

Supporting file Cardio.

## Data Availability

The data supporting the findings of this study are derived from previously published articles and are included within the article and its supporting materials.
