# Supplementary material for: Updated Meta‐Analysis of Left Bundle Branch Area Pacing Versus Right Ventricular Pacing in Conduction System Disorders: Insights From New Evidence
Source: Clin Cardiol. 2026 Apr 9;49(4):e70278. doi: 10.1002/clc.70278 (PMC13065497; doi:10.1002/clc.70278)
Supplement: Supplementary file 1 — Supporting file Cardio. [file CLC-49-e70278-s001.docx]

**SUPPLEMENTARY FILES:**

**Supplementary Table S1:**

| **ROB assessment of observational studies using NOS scale.** | | | | | |
| --- | --- | --- | --- | --- | --- |
| **Author Name** | **Year** | **Selection** | **Comparability** | **Outcome** | **Overall NOS** |
| Zhu H. et al., 2023 | 2023 | 4/4 | 1/2 | 3/3 | 8/9 |
| Zhu H. et al., 2021 | 2021 | 4/4 | 2/2 | 3/3 | 9/9 |
| Zhang S. et al. | 2021 | 3/4 | 0/2 | 3/3 | 6/9 |
| Ramos-Maqueda et al. | 2024 | 4/4 | 0/2 | 3/3 | 7/9 |
| Mao Y. et al. | 2023 | 3/4 | 1/2 | 3/3 | 7/9 |
| Mao Y. et al. | 2024 | 4/4 | 1/2 | 3/3 | 8/9 |
| Lee-KY et al | 2025 | 4/4 | 1/2 | 2/3 | 7/9 |
| DelEra G | 2024 | 4/4 | 2/2 | 3/3 | 9/9 |
| Chen et al | 2025 | 4/4 | 1/2 | 2/3 | 7/9 |
| Chen et al | 2023 | 3/4 | 1/2 | 3/3 | 7/9 |
| Zhang J.M et al | 2019 | 4/4 | 0/2 | 3/3 | 7/9 |
| Wang Q et al. | 2024 | 4/4 | 2/2 | 3/3 | 9/9 |
| Wang Z. et al 2021 | 2021 | 4/4 | 1/2 | 3/3 | 8/9 |
| Sun Z. et al | 2020 | 4/4 | 2/2 | 2/3 | 8/9 |
| P.S Sharma et al | 2022 | 4/4 | 2/2 | 3/3 | 9/9 |
| Okubo et al | 2022 | 4/4 | 2/2 | 3/3 | 9/9 |
| Niu H.X.et al. | 2021 | 4/4 | 2/2 | 3/3 | 9/9 |
| Mayajima et al | 2022 | 4/4 | 1/2 | 3/3 | 8/9 |
| Liu X. et al | 2022 | 4/4 | 2/2 | 2/3 | 8/9 |
| Liu et al. | 2022 | 4/4 | 2/2 | 3/3 | 9/9 |
| Li X et al | 2021 | 4/4 | 2/2 | 3/3 | 9/9 |
| Li W et al | 2022 | 4/4 | 2/2 | 3/3 | 9/9 |
| Chen X. et al | 2020 | 4/4 | 1/2 | 2/3 | 7/9 |
| Chen X. et al | 2022 | 4/4 | 2/2 | 2/3 | 8/9 |
| Chen K. et al | 2018 | 4/4 | 2/2 | 2/3 | 8/9 |
| Cai B. 2020 | 2020 | 3/4 | 2/2 | 2/3 | 7/9 |
| Byeon K. | 2020 | 4/4 | 2/2 | 2/3 | 9/9 |
| Zhang et al | 2024 | 4/4 | 2/2 | 2/3 | 8/9 |
| Yao et al | 2025 | 4/4 | 1/2 | 3/3 | 8/9 |
| Wang X. et al | 2024 | 4/4 | 1/2 | 3/3 | 8/9 |
| Ramalingam et al | 2024 | 4/4 | 1/2 | 3/3 | 8/9 |
| Palmisano et al | 2023 | 4/4 | 2/2 | 3/3 | 9/9 |
| Okubo et al | 2025 | 4/4 | 2/2 | 3/3 | 9/9 |
| Kono et al. | 2025 | 4/4 | 2/2 | 3/3 | 9/9 |

| **ROB assessment of Clinical trials using Cochrane ROB Tool 2.0** | | | | | | | |
| --- | --- | --- | --- | --- | --- | --- | --- |
| **Author** | **Year** | **Randomization** | **Deviations** | **Missing** | **Measurement** | **Reporting** | **Overall** |
| Yao et al. | 2022 | 1/1 | 1/1 | 1/1 | 1/1 | 1/1 | 5/5 |
| Wang J.F. et al. | 2019 | 1/1 | 1/1 | 1/1 | 1/1 | 1/1 | 5/5 |
| Riano Ondiviela et al. | 2021 | 1/1 | 1/1 | 1/1 | 1/1 | 1/1 | 5/5 |
| Liu Q et al | 2021 | 0/1 | 1/1 | 1/1 | 1/1 | 1/1 | 4/5 |
| Das et al. | 2020 | 0/1 | 1/1 | 1/1 | 1/1 | 1/1 | 4/5 |
| Zhao et al | 2023 | 1/1 | 0/1 | 1/1 | 0/1 | 1/1 | 3/5 |

**Supplementary Table S2:**

| **Ventricular Pacing Burden Across The Included Studies** | | | | |
| --- | --- | --- | --- | --- |
| **Sr. no** | **Name of the Study** | **Year of Publication** | **Ventricular Pacing Burden (VP Burden)** | |
|  |  |  | **LBBAP (mean +- SD or median (IOQR)** | **RVP (mean +- SD or median (IOQR)** |
| 1 | Chen | 2023 | 83.81 ± 28.69 | 82.94 ± 29.29 |
| 2 | Lee-Ky | 2025 | 95.5 ± 13.5 | 86.3 ± 25.4 |
| 3 | Zhang | 2024 | 99.6 ± 1.0 | 88.1 ± 20.9 |
| 4 | Zhang S. | 2021 | 95.47 ± 1.22 | 94.86 ± 1.56% |
| 5 | Wang Z et al | 2021 | 80% | 51% |
| 6 | Wang Q. et al | 2024 | >20% = 67 >40%=65.1 | >20%= 48.1 >40%= 37.3 |
| 7 | Sun Z. | 2020 | >70% | >70% |
| 8 | Sharma et al | 2022 | >20% = 72.8 >40%= 71.2 | >20%= 57.4 >40%= 50.5 |
| 9 | Okubo | 2025 | 90.8±20.4 | 86.2±22.6 |
| 10 | Okubo | 2022 | 84.8 ± 25.5 | 79.9 ± 24.6 |
| 11 | Niu H. X. et al | 2021 | 91.6 ± 7.1 | 91.3 ± 10.0 |
| 12 | Miyajima et al | 2022 | 100 ± 0.0 | 100 ± 0.0 |
| 13 | Wang Q | 2024 | >20% = (73/109), >40% = 71/109 | >20% = (76/158), >40% = (59/158) |

**Supplementary Table S3.**

| **Supplementary Table S5. Follow-up timepoints reported in included studies and the timepoints selected for meta-analysis of structural outcomes (LVEF and LVEDD)** | | | |
| --- | --- | --- | --- |
| **Author Names** | **Outcomes** | **Follow-up Timepoints Reported** | **Timepoint Used for Meta-analysis** |
| Sun Z 2020 | LVEF | Baseline, intraoperative, 7 days f/u | 7 Days F/U |
| Liu et al. 2022 | LVEF, LVEDD | Baseline, and 14 months F/U | 14 months F/U |
| Yao et al. 2022 | LVEF | Baseline, 6m, 12m, 18m | 12 months F/U |
| Okubo et al. 2022 | LVEF, LVEDD | Baseline, 6months | 6 months F/U |
| Ramalingam et al. 2024 | LVEF | Baseline, 6 months | 6 months F/U |
| Liu X. et al 2022 | LVEF, LVEDD | Baseline, last f/u (13 months) | 13 months F/U |
| Zhang 2024 | LVEF | Baseline, 12 months f/u | 12 months F/U |
| Okubo et al. 2025 | LVEF, LVEDD | Baseline, 12 months f/u | 12 months F/U |
| Chen 2025 | LVEF, LVEDD | Baseline, 12 months f/u | 12 months F/U |
| Li X. et al 2021 | LVEF, LVEDD | Baseline, 6, and 12m | 12 months F/U |
| Yao et al. 2025 | LVEF, LVEDD | Baseline, last f/u (52-53 months) | Last F/U |
| Zhao 2023 | LVEF | Baseline, 3m and 6m | 6 months F/U |
| Das et al. 2020 | LVEF, LVEDD | Baseline, 6 months | 6 months F/U |
| Niu et al. 2021 | LVEF, LVEDD | Baseline, 15 months | 15 months F/U |
| Mao Y 2023 | LVEF, LVEDD | Baseline, 12 months F/U | 12 months F/U |
| Li W. et al 2022 | LVEF, LVEDD | Baseline, 1w, 3m, 6m, 12 m | 12 months F/U |
| Wang X 2024 | LVEDD | Baseline, 1y, 2y, 3y, 4y, 5y | 12 months F/U |

**Supplementary Table S4. Pooled baseline characteristics of patients undergoing LBBAP versus RVP.**

| **Outcome** | **Measure** | **Estimate** | **Lower CI** | **Upper CI** | **P-value** | **I2** | **Studies Included** |
| --- | --- | --- | --- | --- | --- | --- | --- |
| **Age** | MD | -0.966 | -1.494 | -0.437 | 0.0003416 | 0.13 | 38 |
| **Baseline LVEF** | MD | -0.704 | -1.32 | -0.088 | 0.02499 | 0.62 | 31 |
| **Atrial fibrillation** | RR | 1.078 | 0.934 | 1.243 | 0.3047 | 0.47 | 28 |
| **Hypertension** | RR | 0.977 | 0.948 | 1.008 | 0.1459 | 0 | 34 |
| **Diabetes mellitus** | RR | 1.069 | 0.969 | 1.18 | 0.1835 | 0.2 | 33 |
| **Coronary artery disease** | RR | 0.967 | 0.863 | 1.082 | 0.5573 | 0.23 | 28 |

**S5: FOREST PLOTS, SENSITIVITY ANALYSES, FUNNEL PLOTS, EGGER’S TESTS**

1. **QRS duration**
2. **LVEF**
3. **LVEDD**
4. **Pacing impedance at time of implantation**
5. **Pacing impedance at time of followup**
6. **Pacing threshold at time of implantation**
7. **Pacing threshold at time of follow up**
8. **R wave amplitude at time of implantation**
9. **R wave amplitude at time of follow-up**
10. **Procedural time**
11. **Flouroscopic time**
12. **Lead Dislodgement**
13. **Lead Revision Rate**
14. **Overall Complications Rate**
15. **Pericardial effusion/tamponade**
16. **Reintervention rate**
17. **Periprocedural mortality**
18. **Septal Perforation**
19. **HF Hospitalizations**
20. **All Cause Mortality**
21. **NT-pro BNP Change**

**I. FOREST PLOTS:**

**A.**

**B.
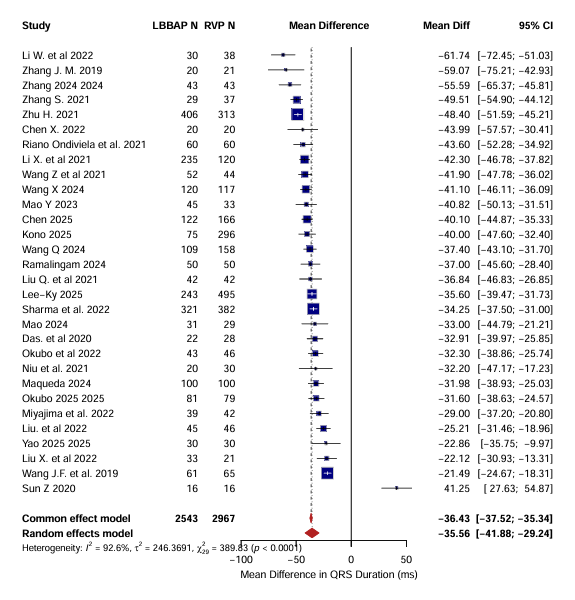
**

**
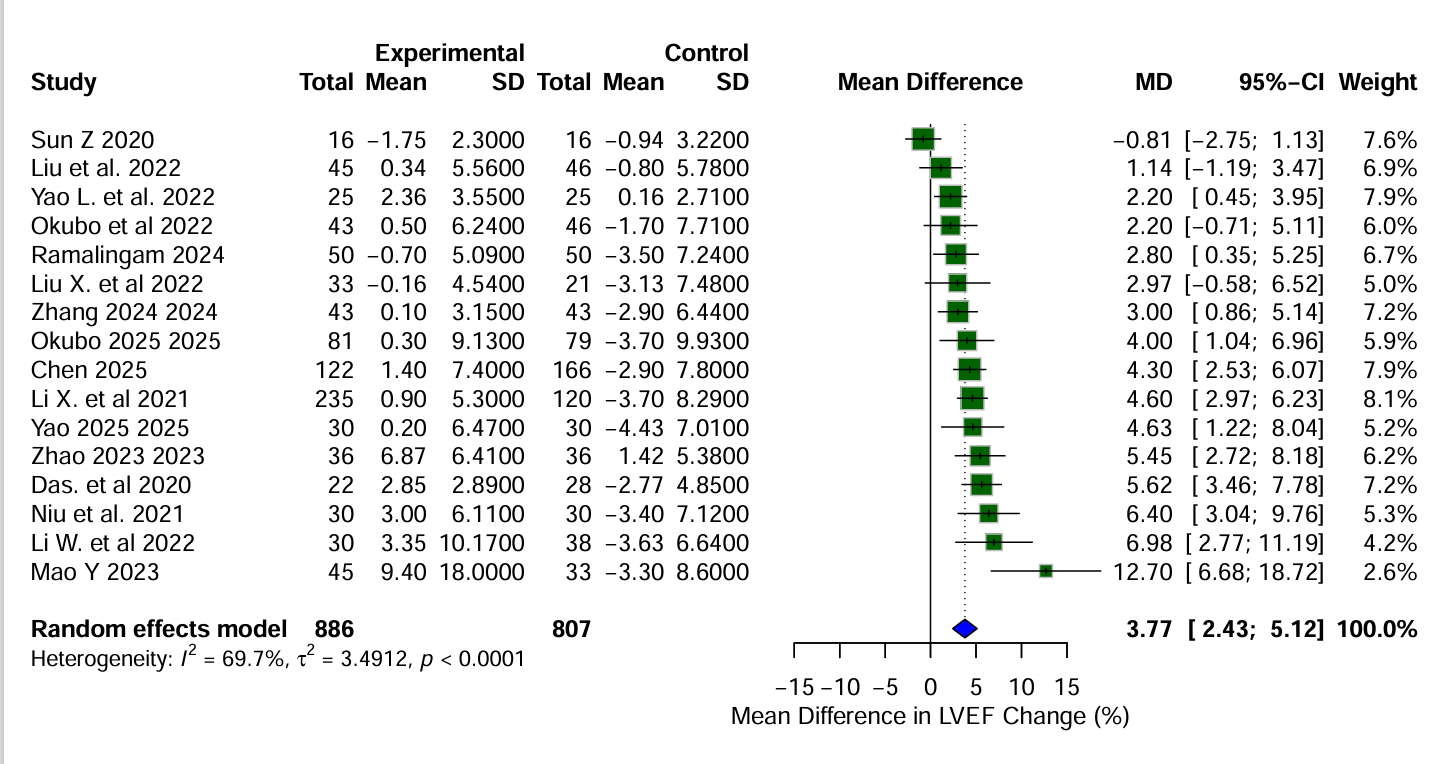
**

**C.**

**
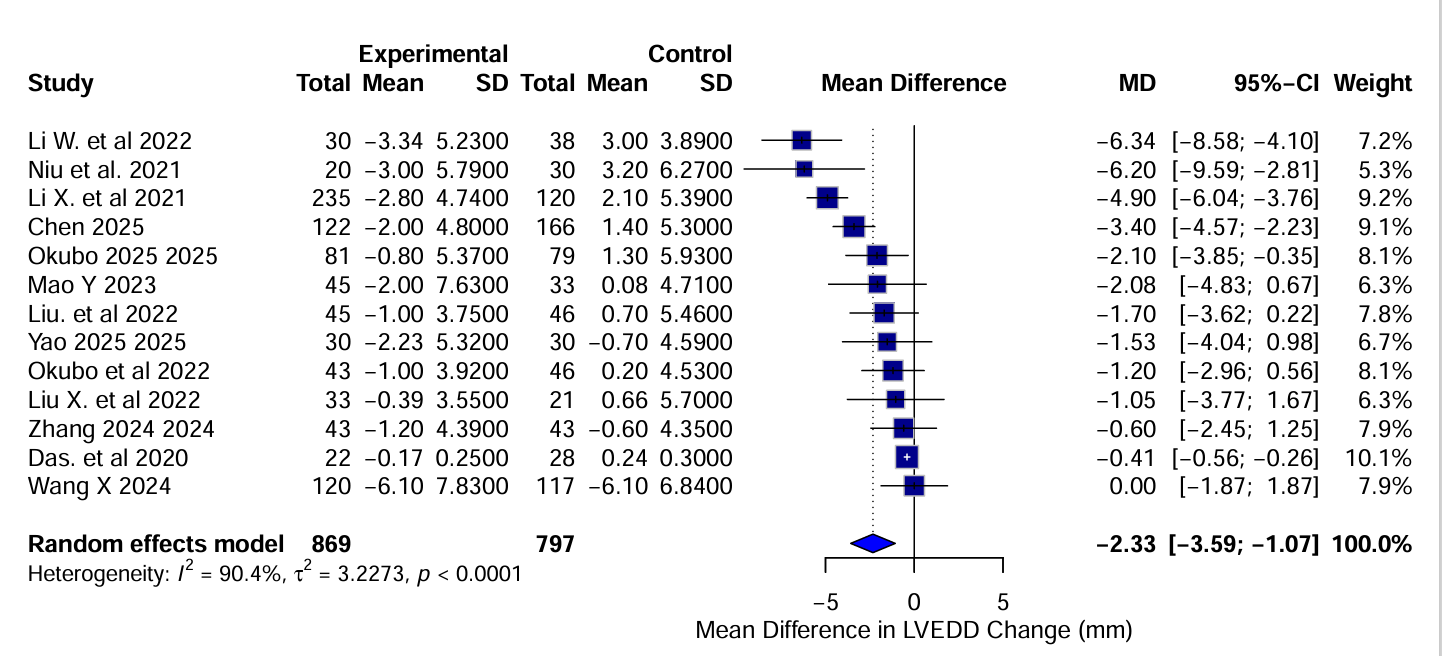
**

**D.
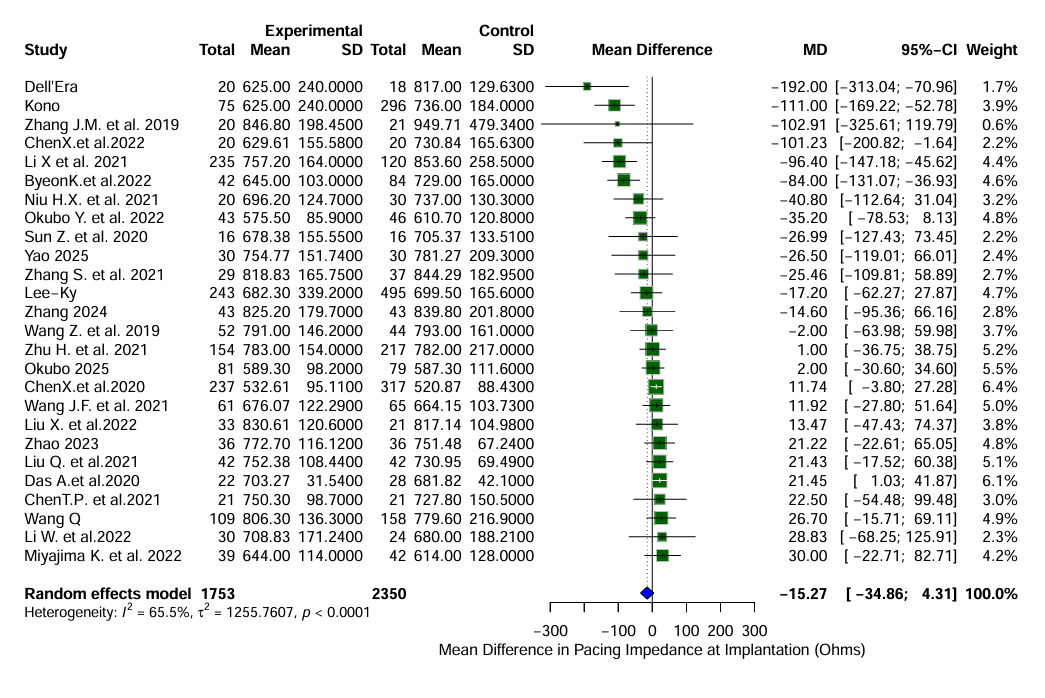
E.
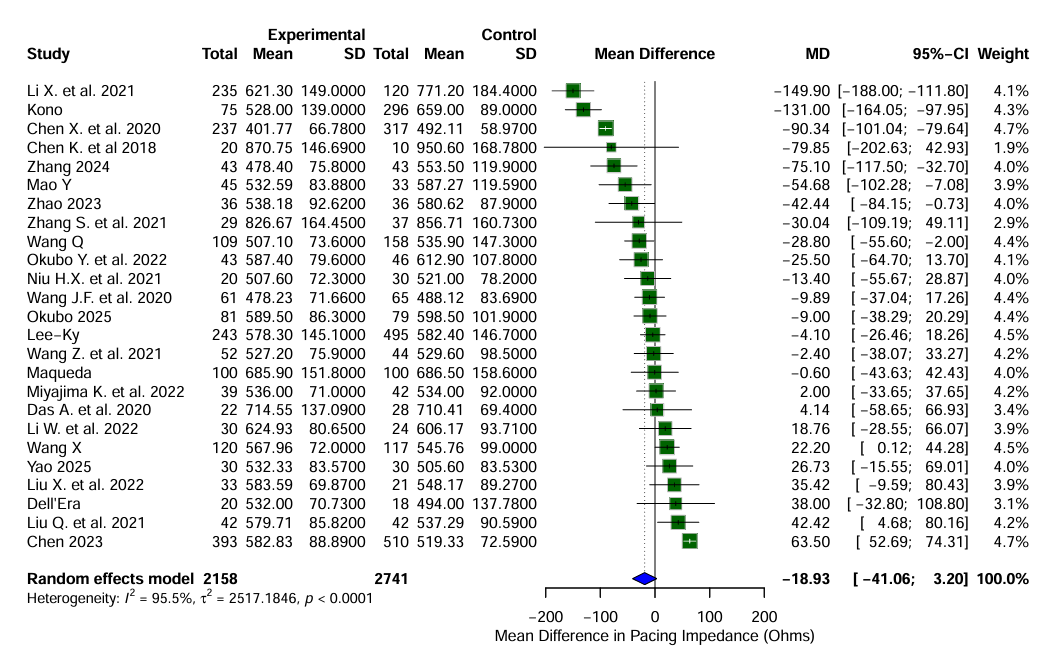
F.
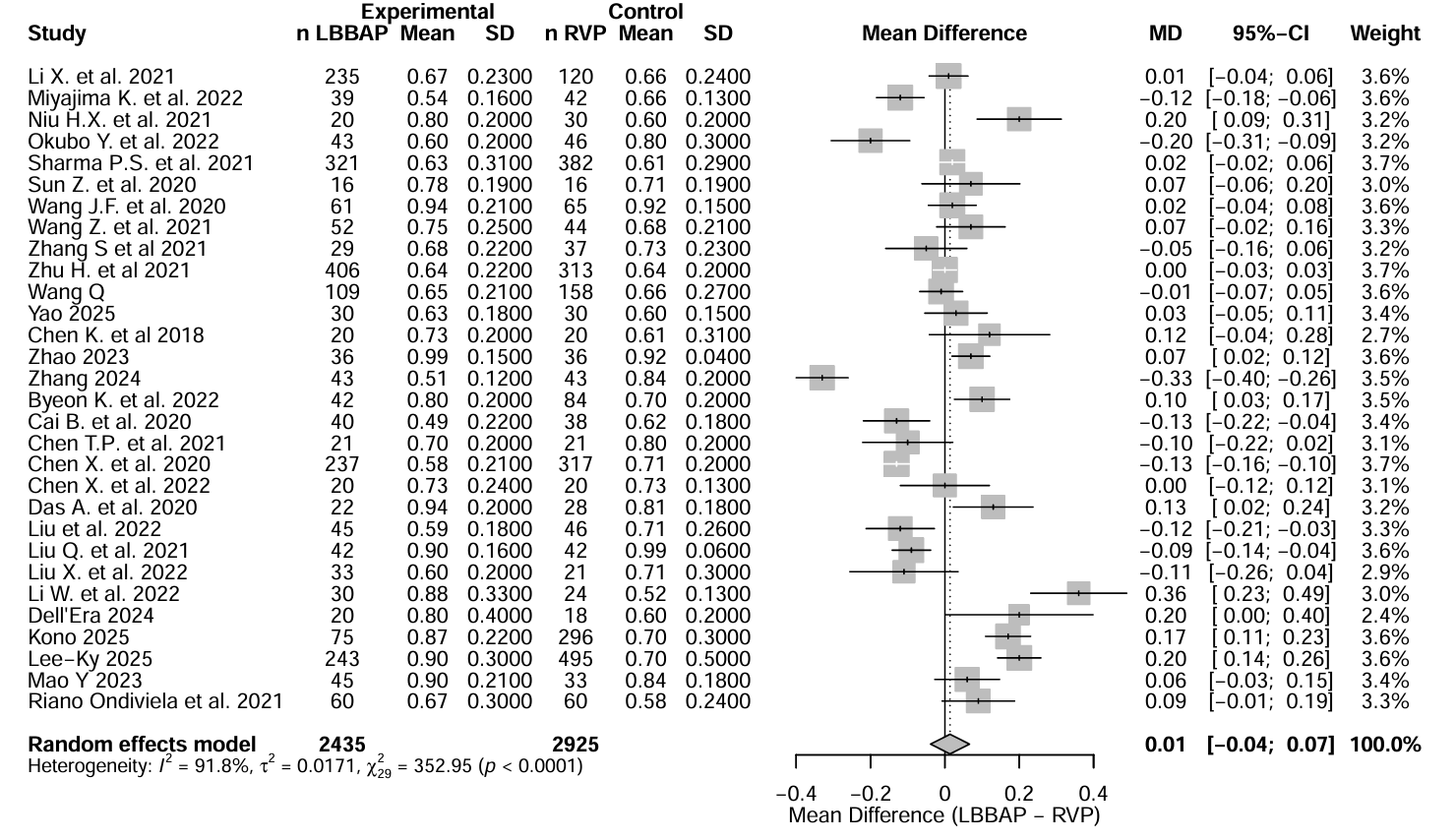
G.
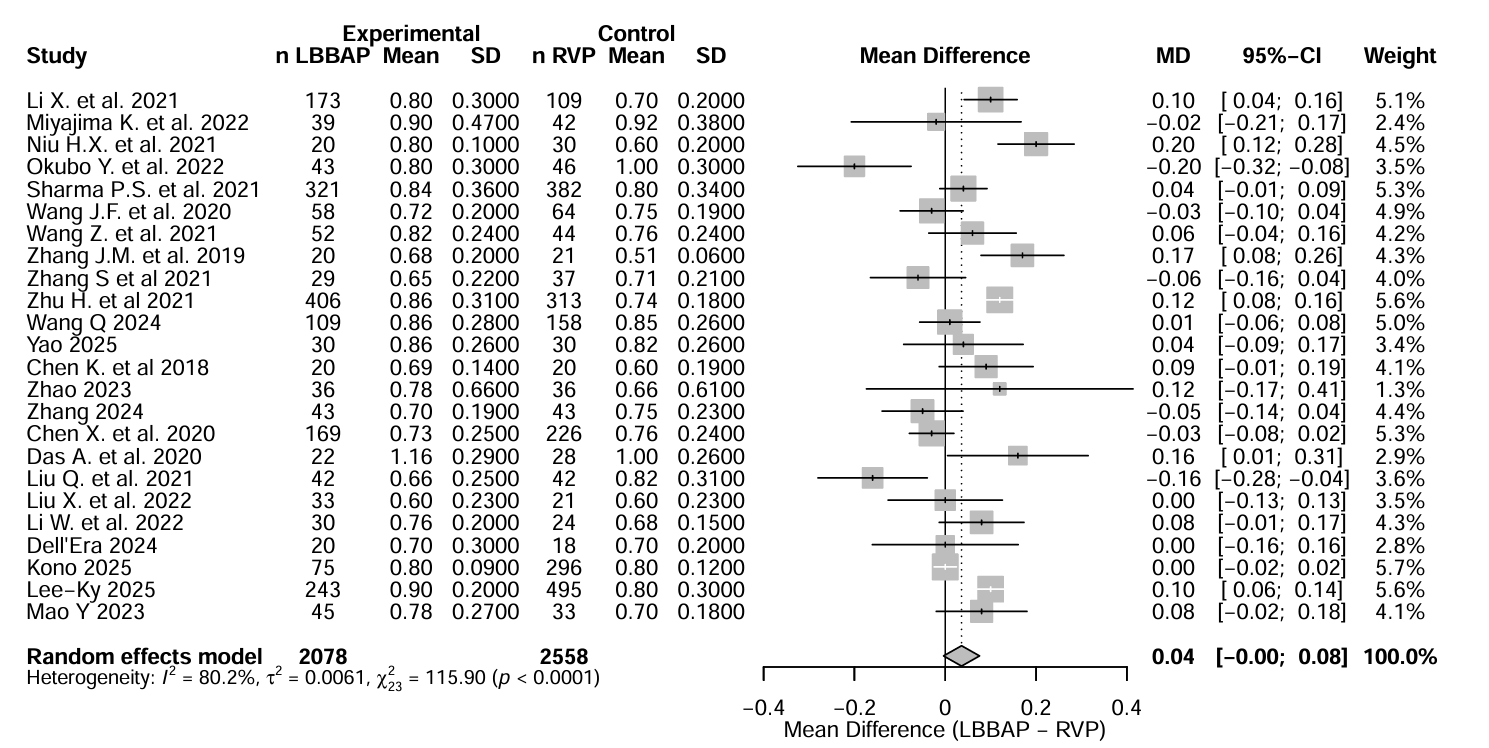
H.
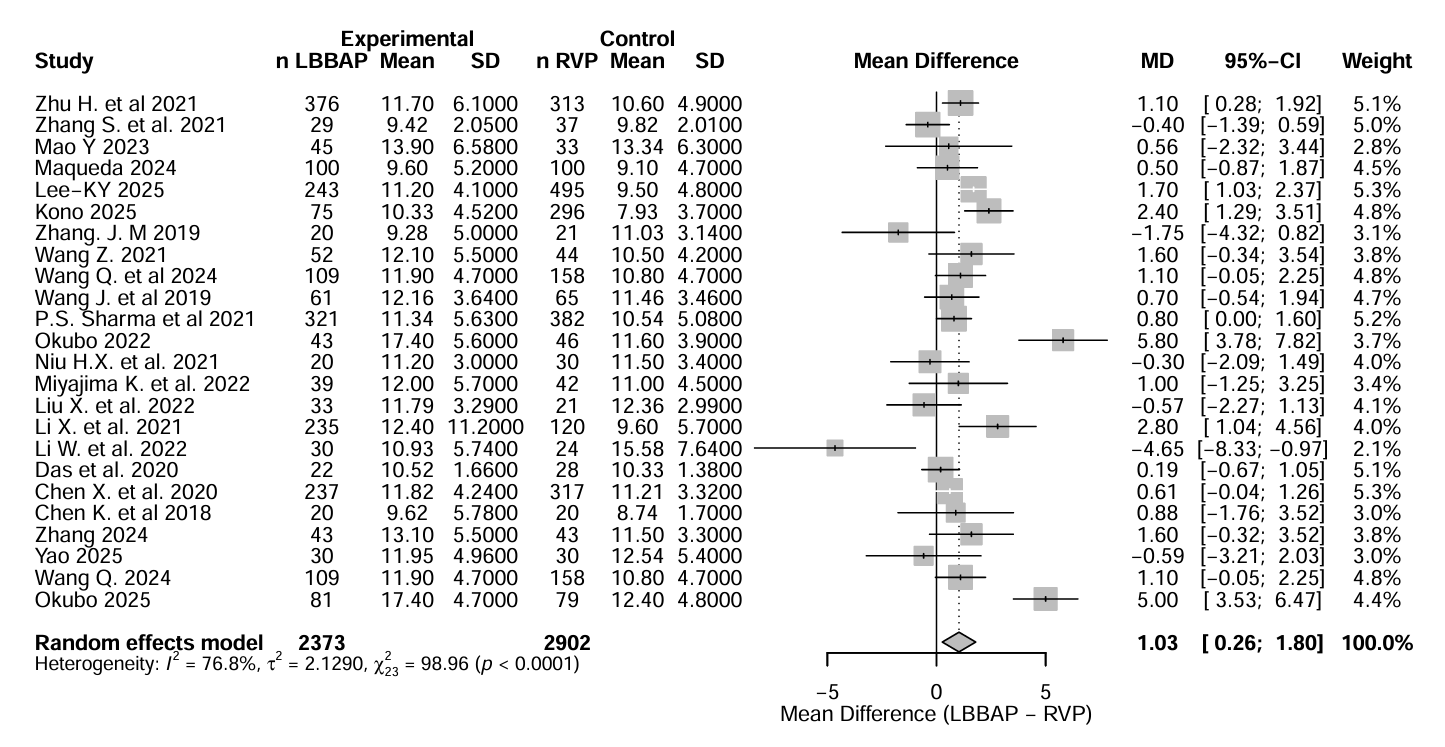
**

**I.**
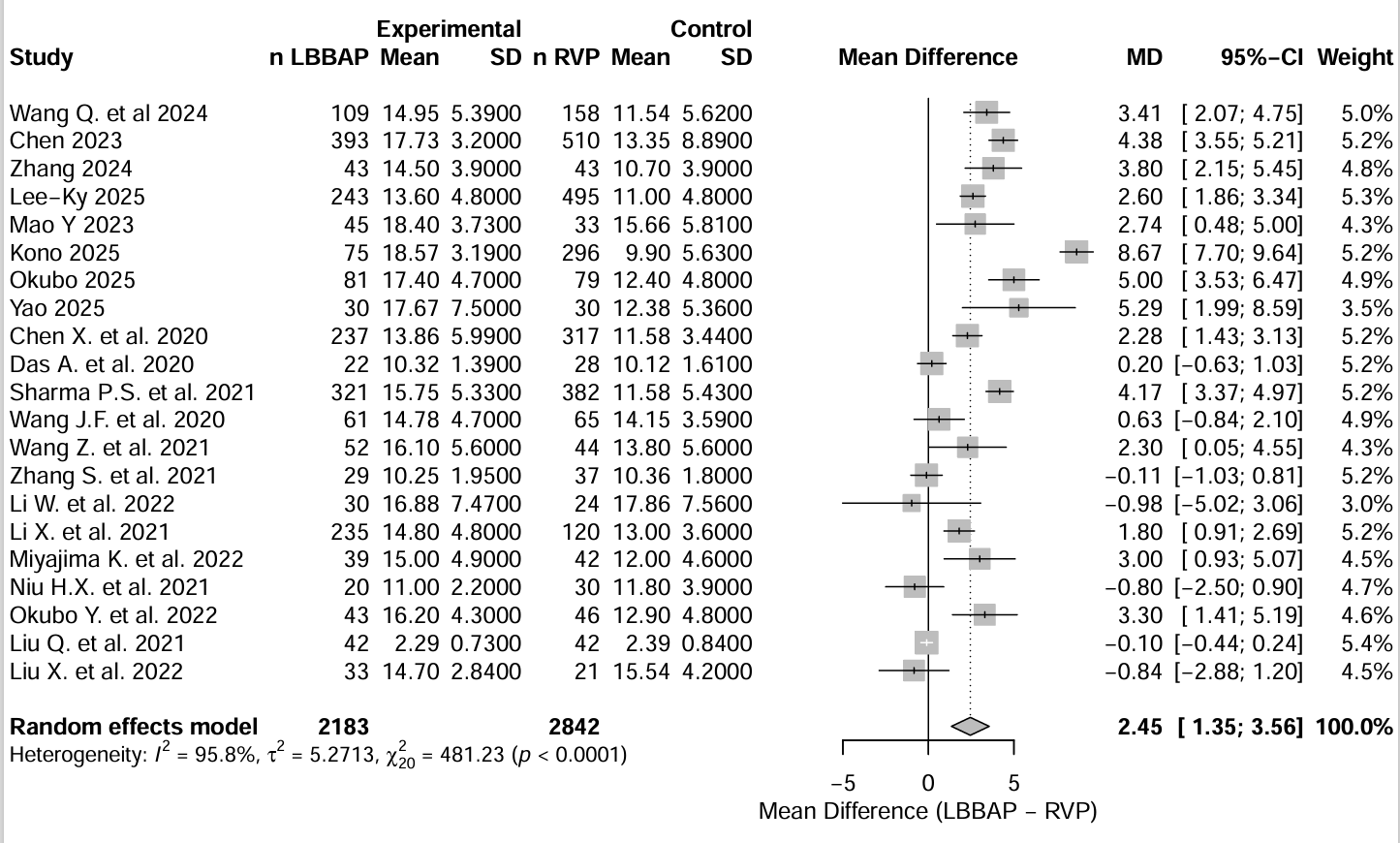
**J.**
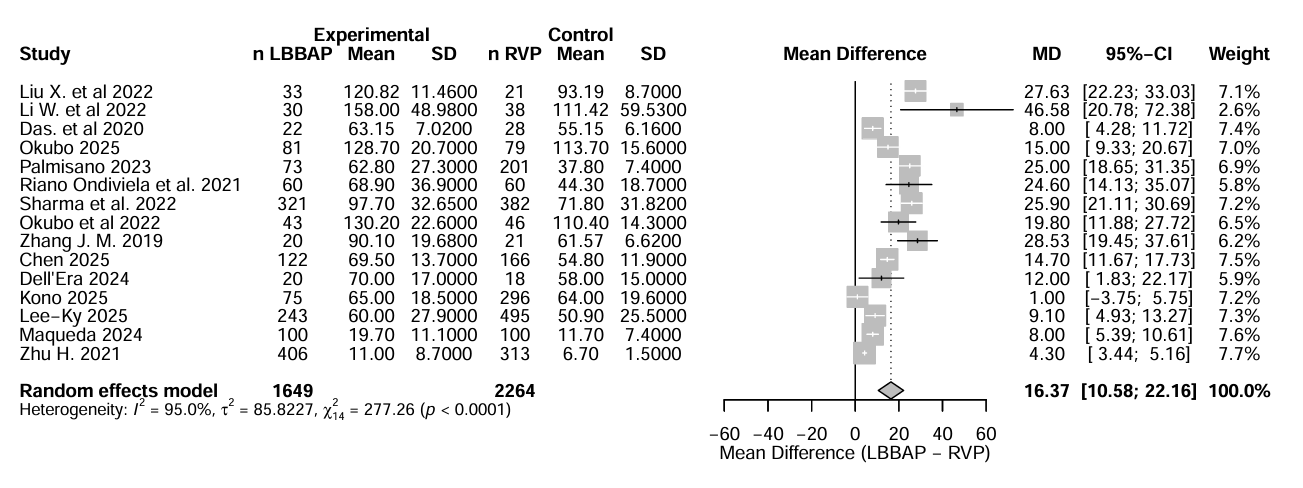
**K.**
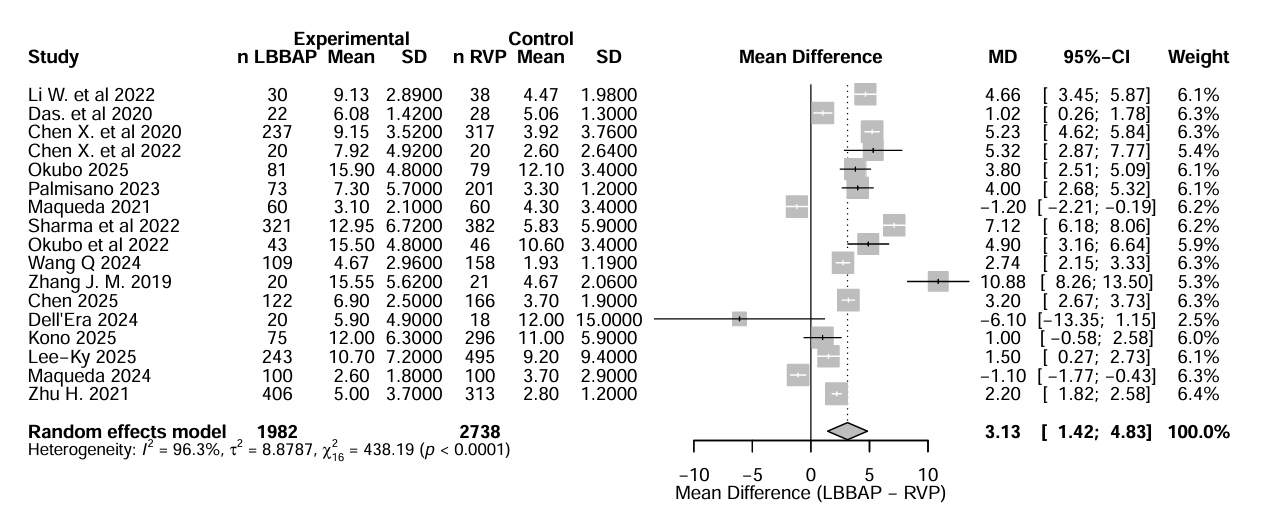


**L.**

**
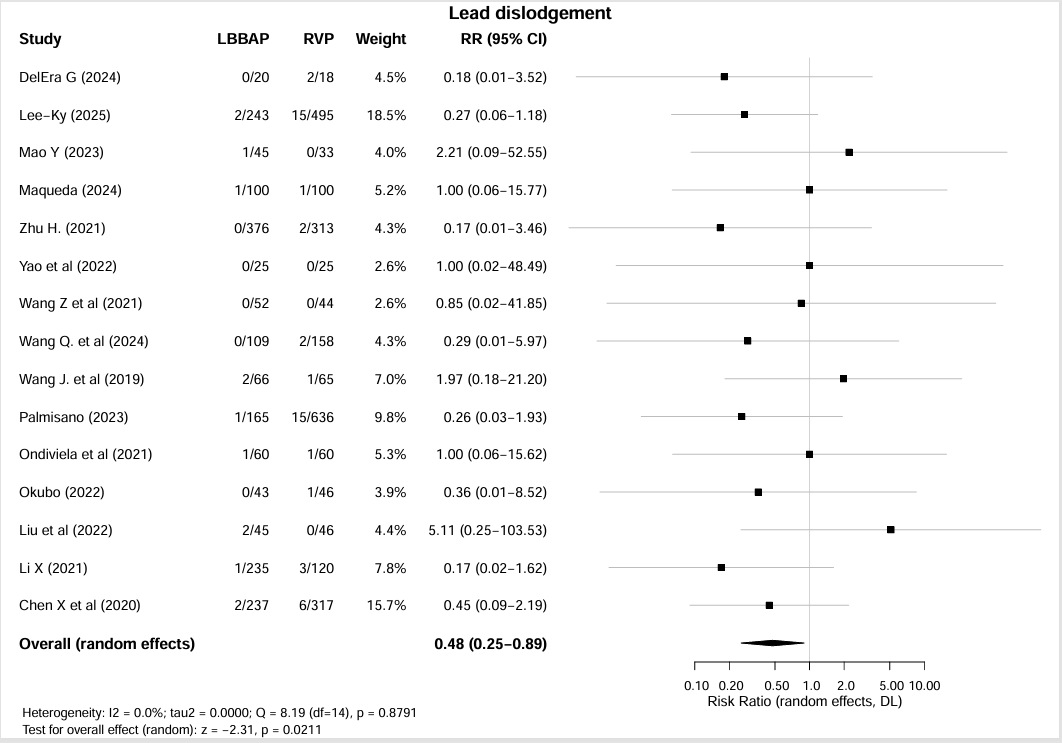
**

**M.**

**
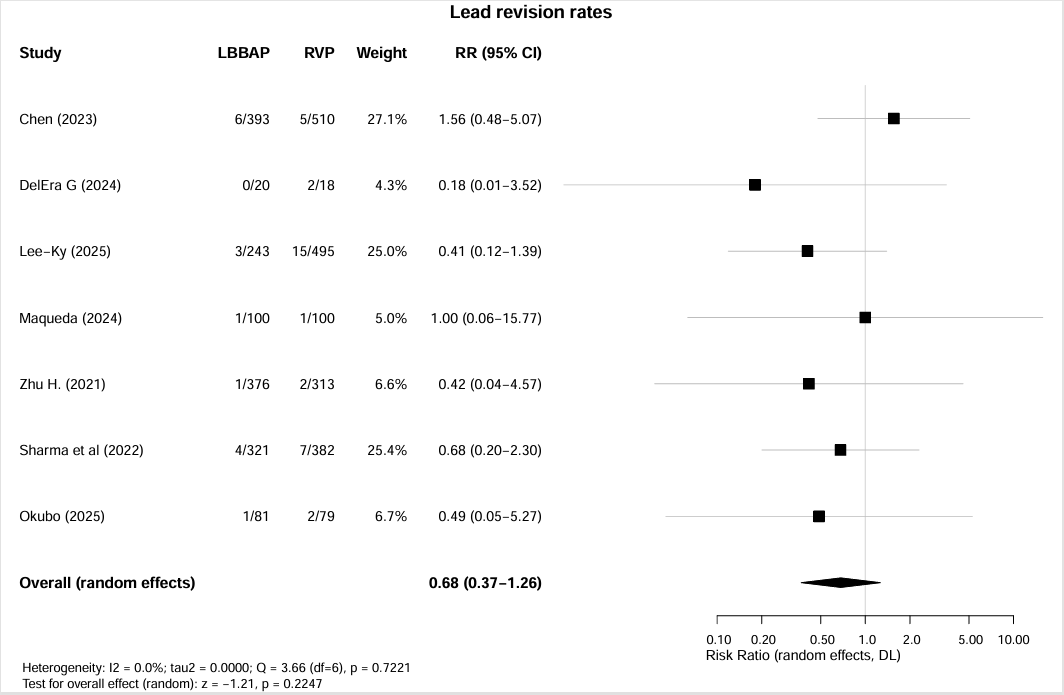
**

**N.**

**
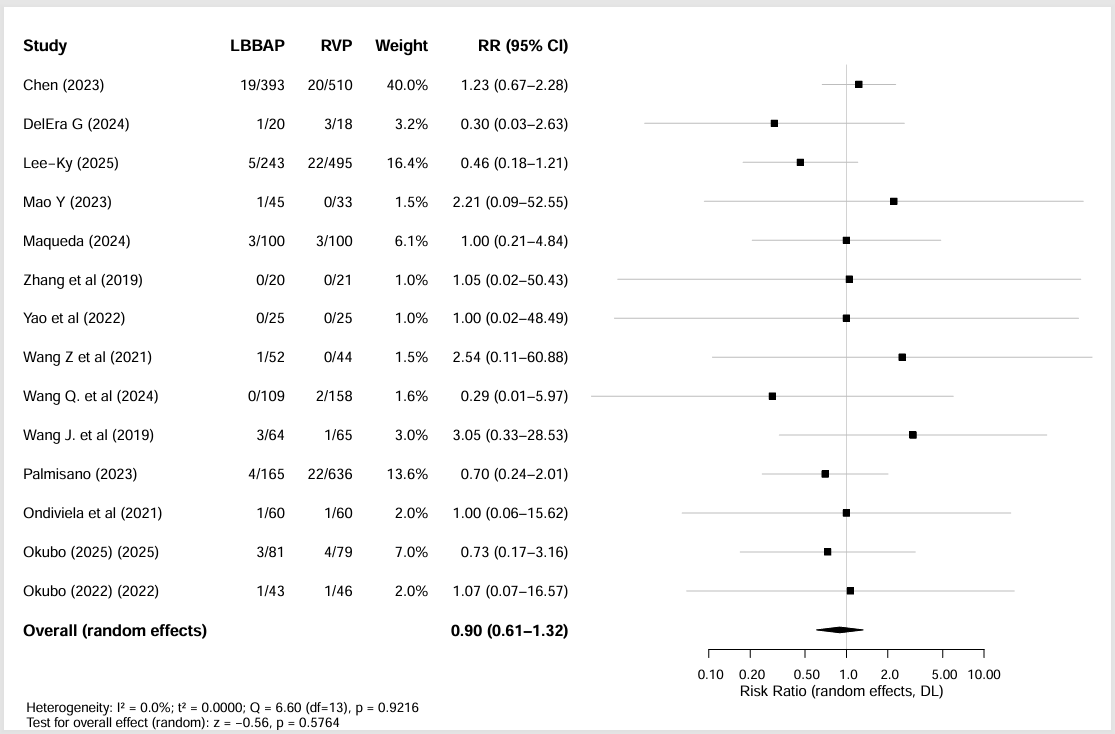
**

**O.**

**
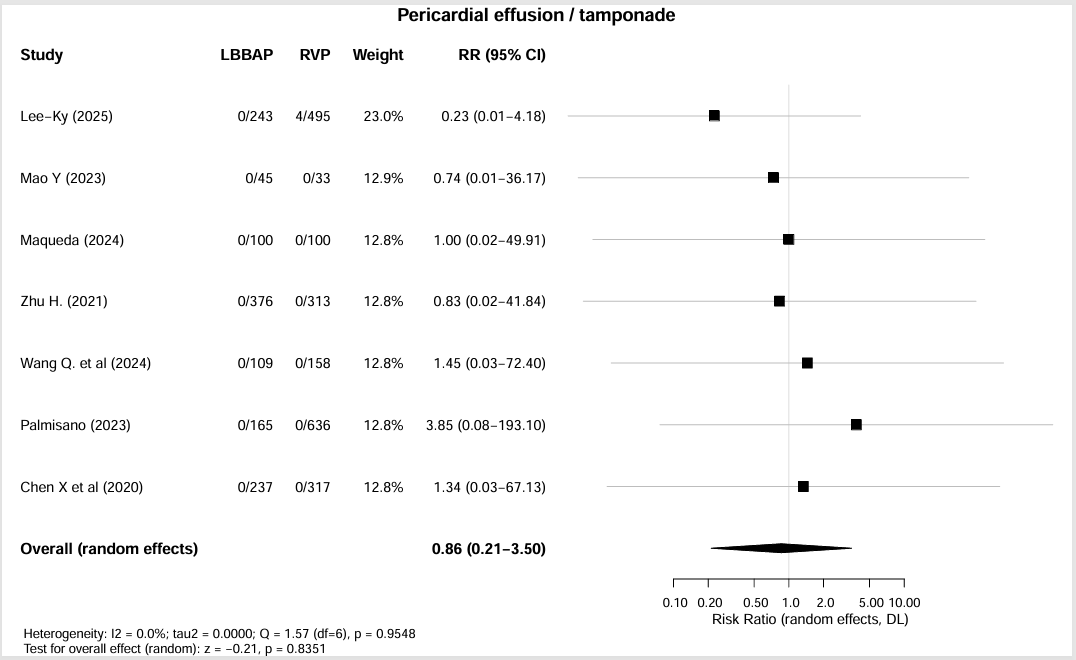
**

**P.**

**
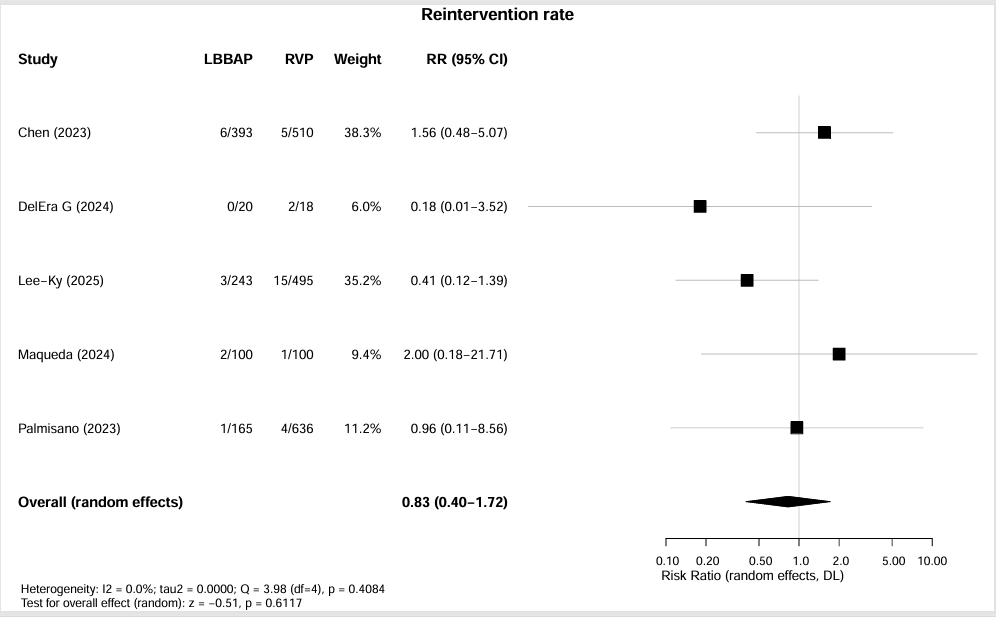
**

**Q.**

**
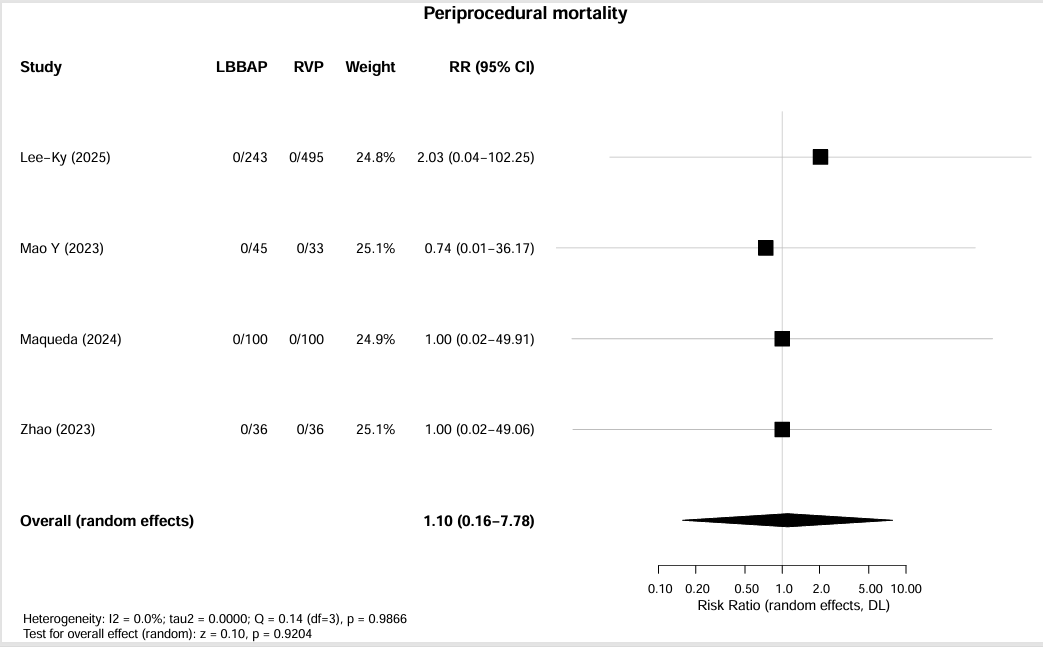
**

**R.**

**
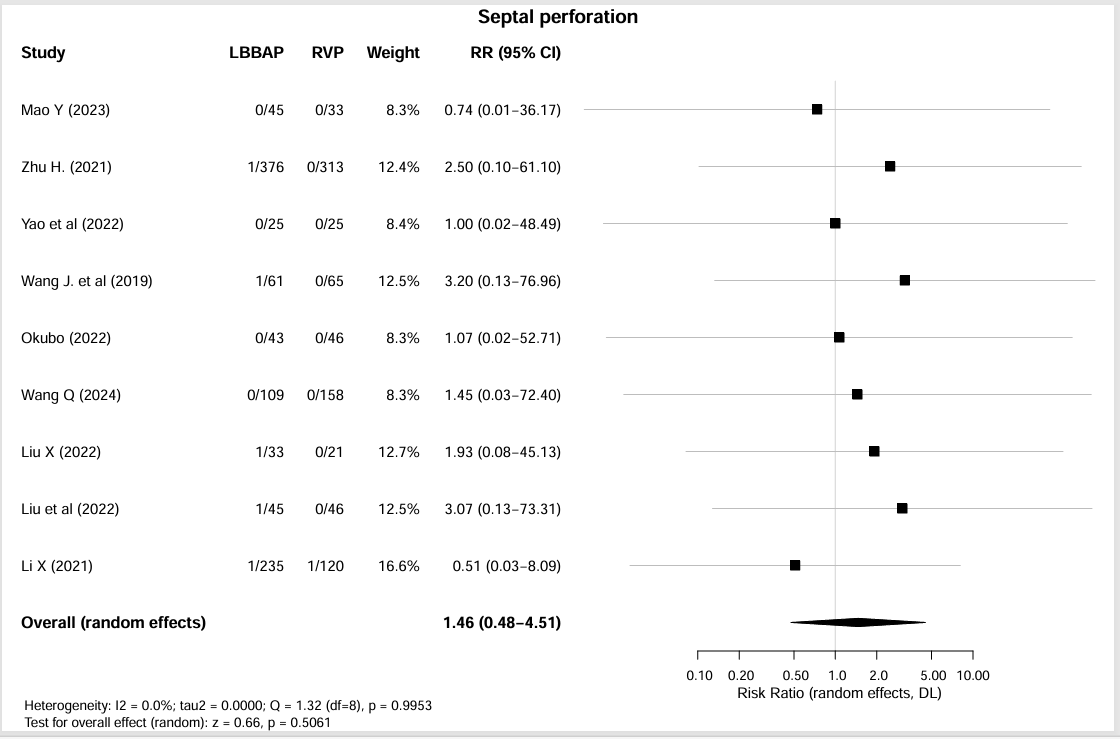
**

**S.**

**
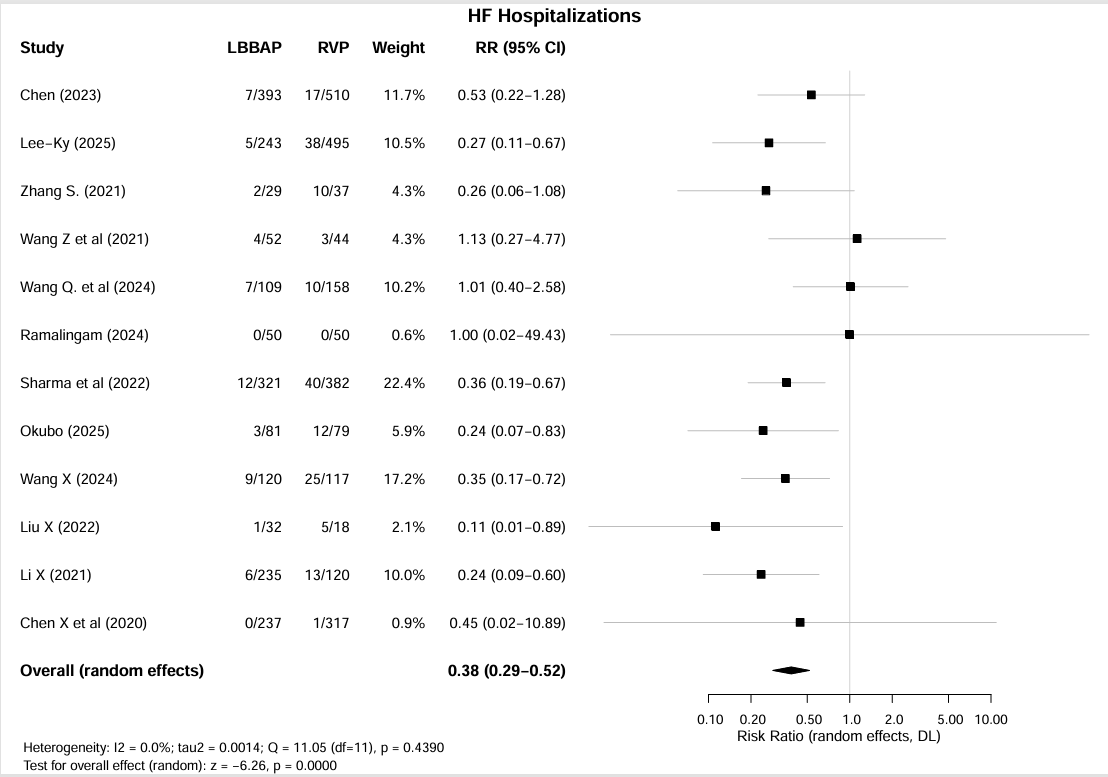
**

**T.**

**
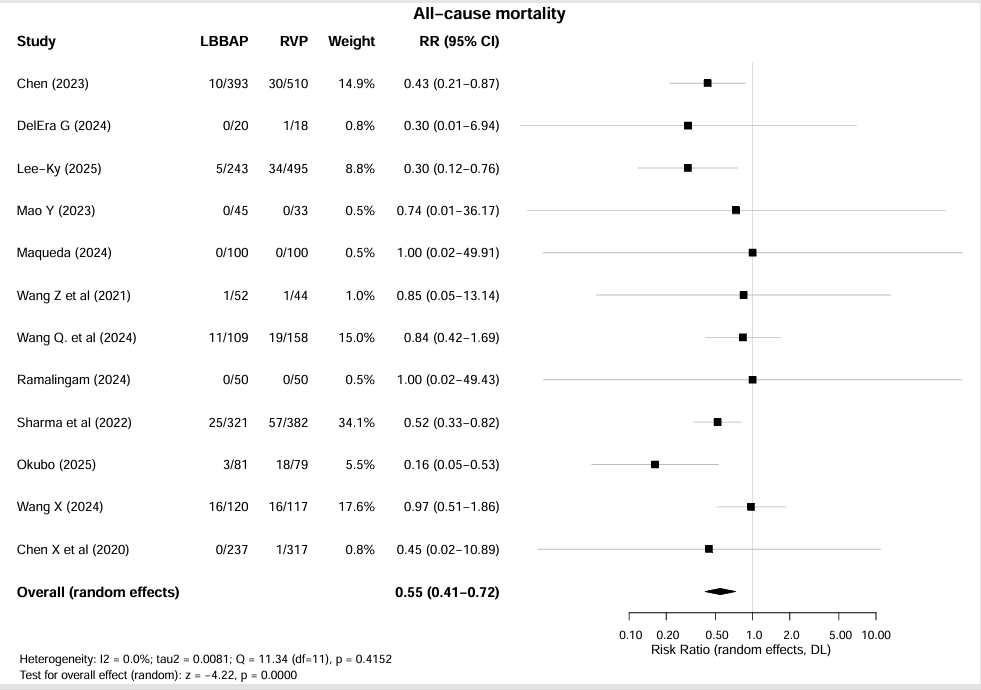
**

**U.**

**
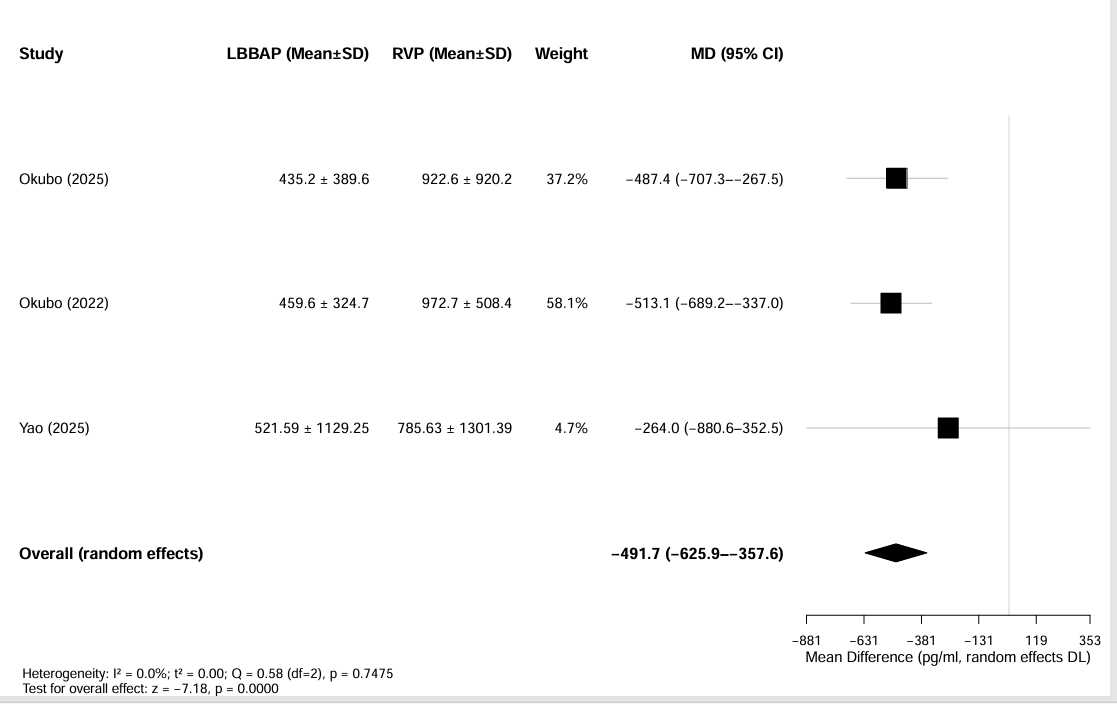
**

**SENSITIVITY ANALYSES:**

**A.
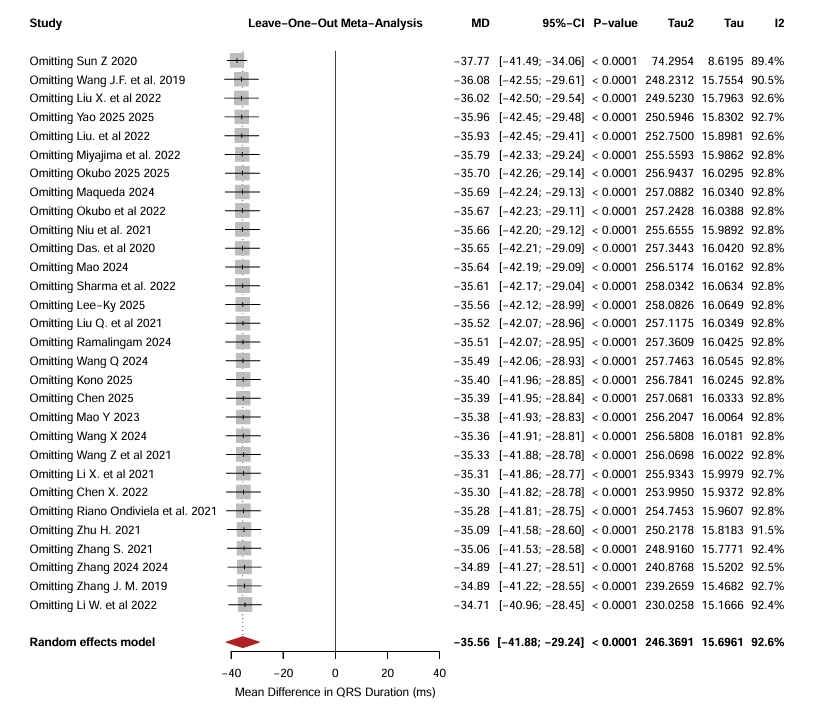
**

**B.
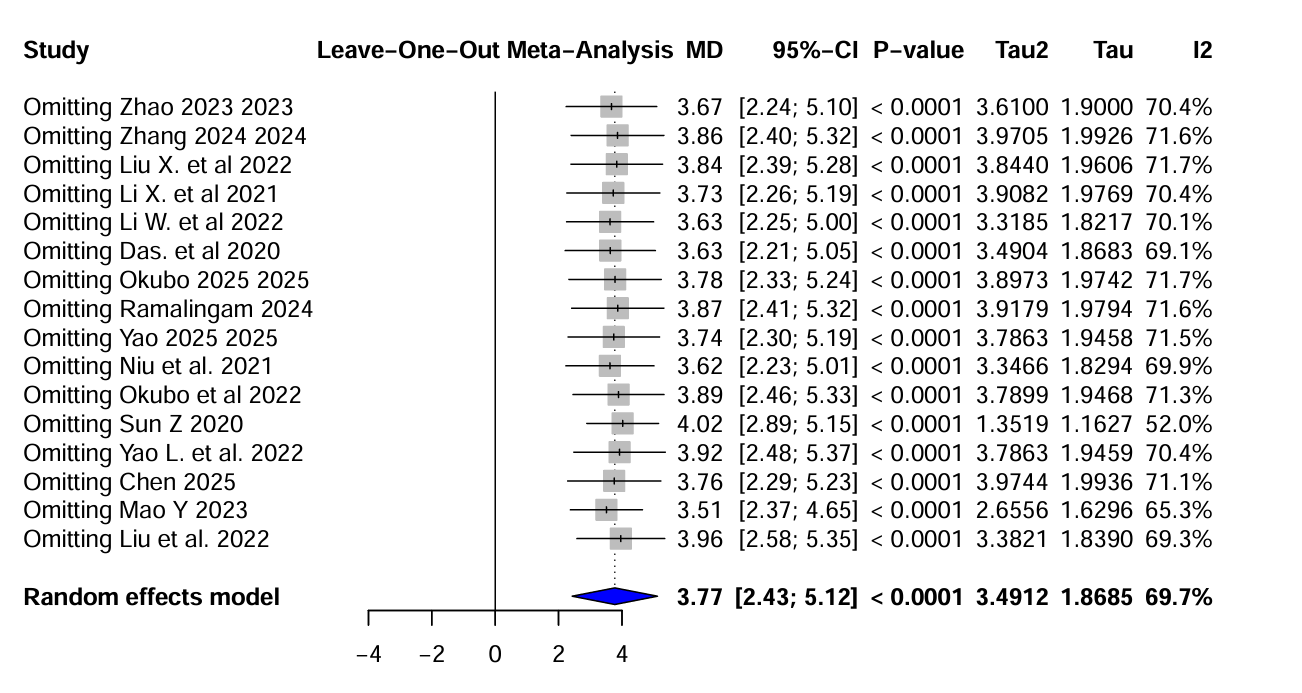
C.
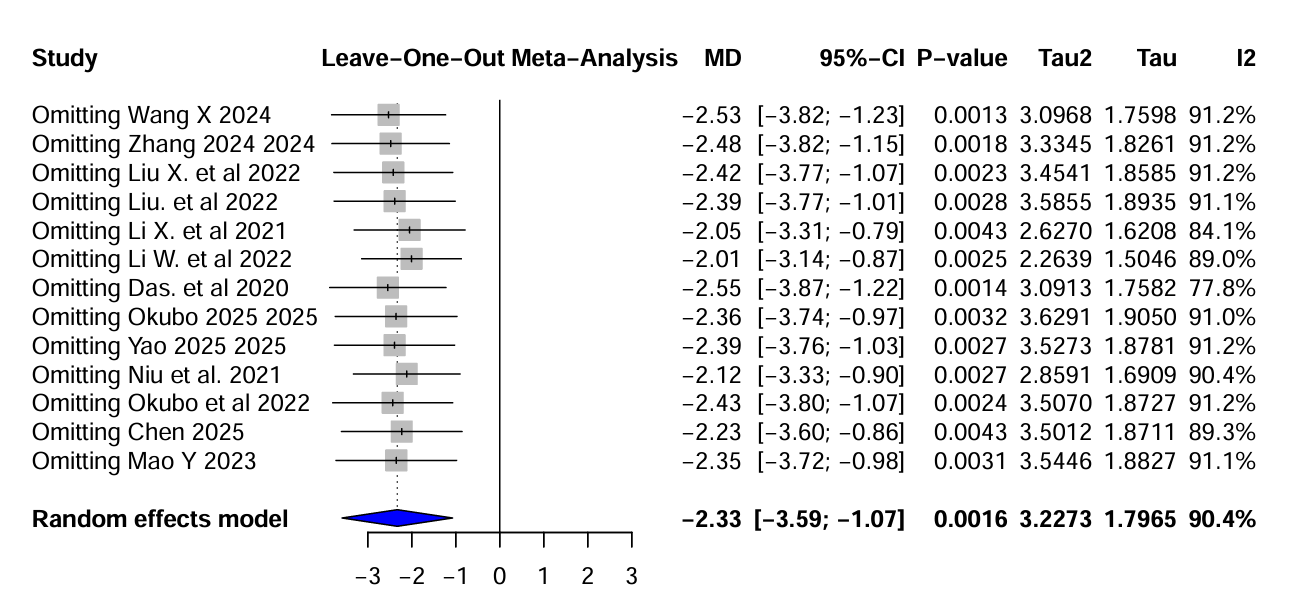
D.
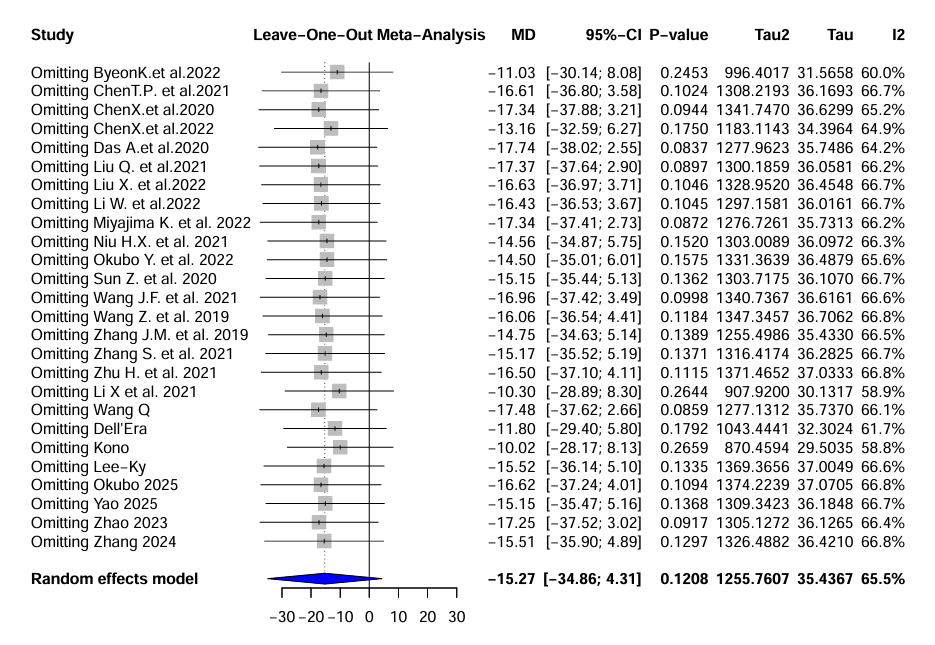
E.
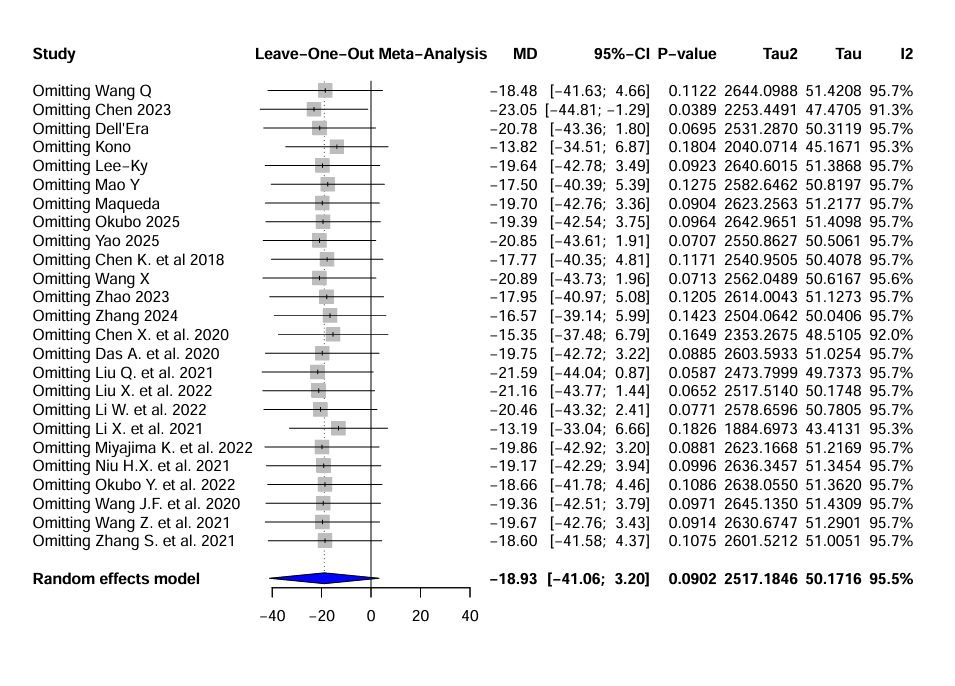
F.
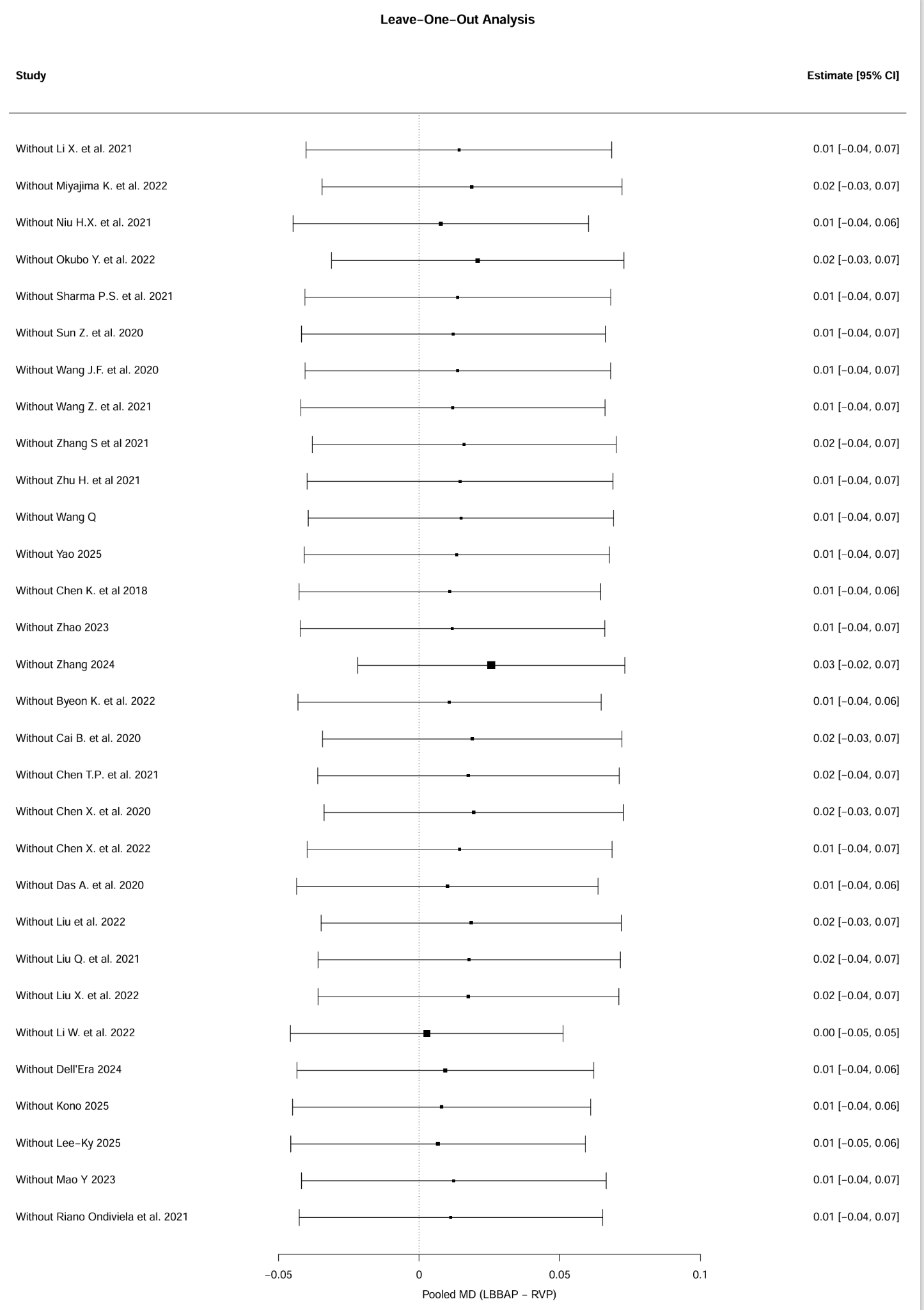
**

**G.
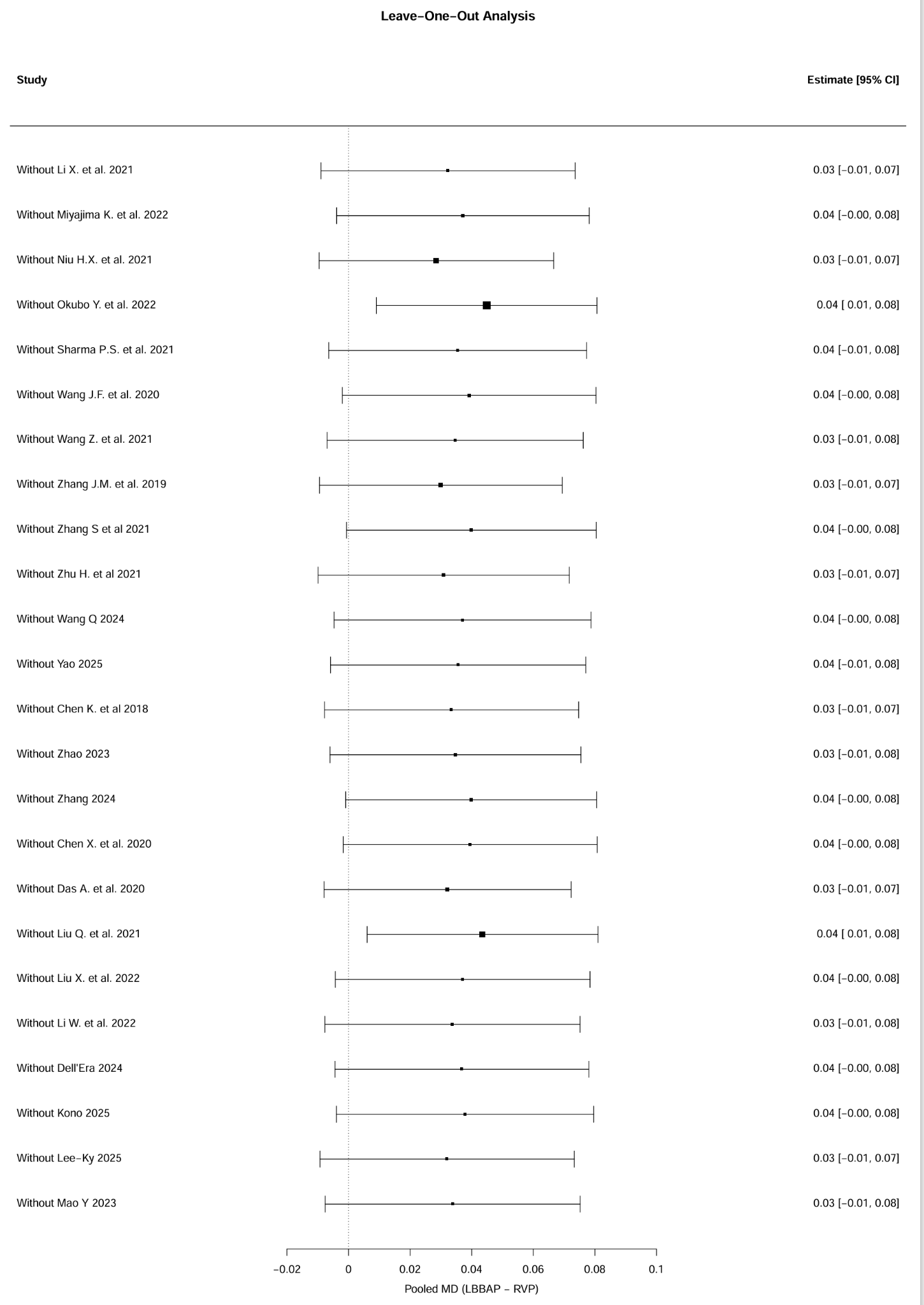
H.
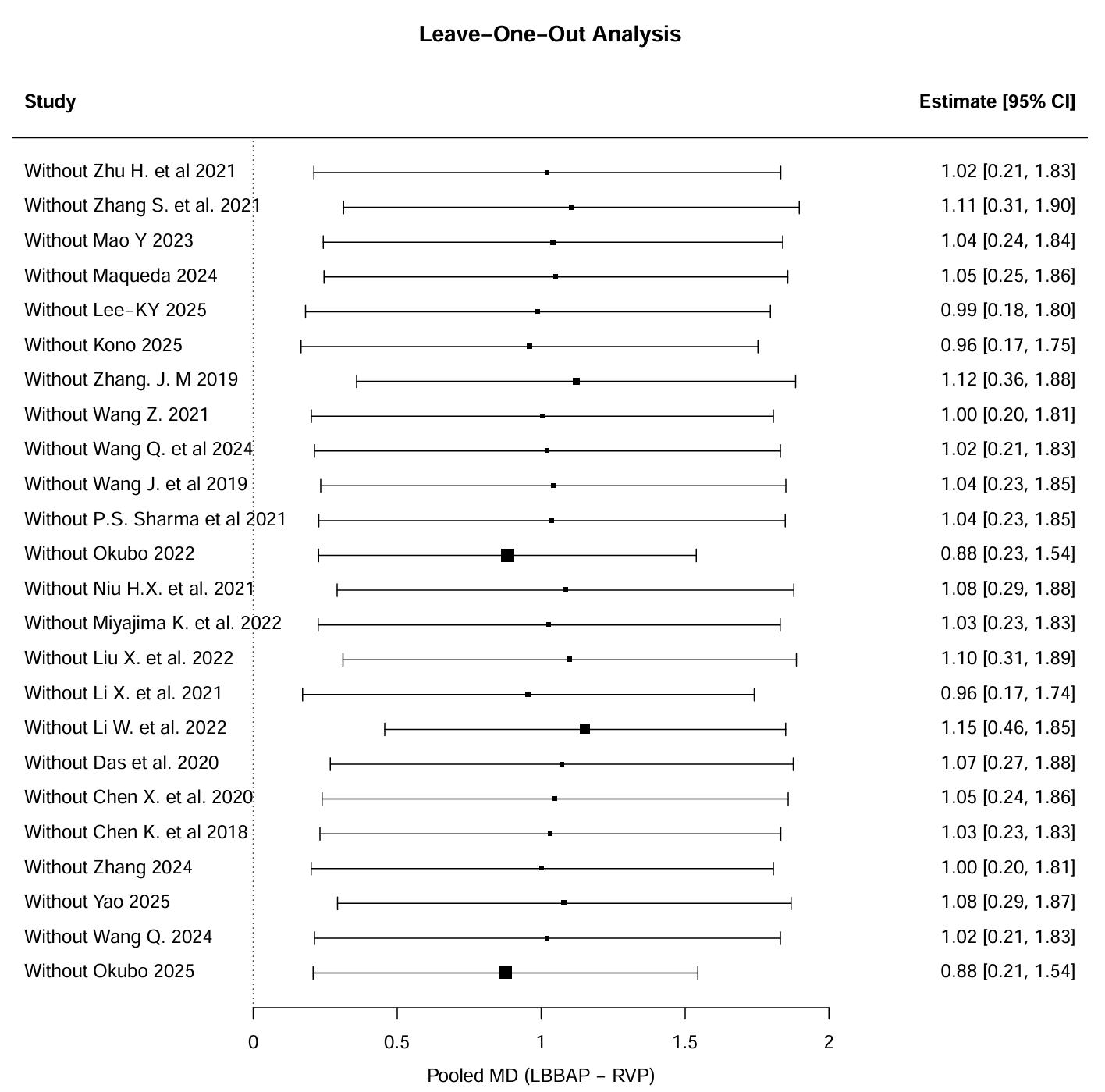
**

**I.
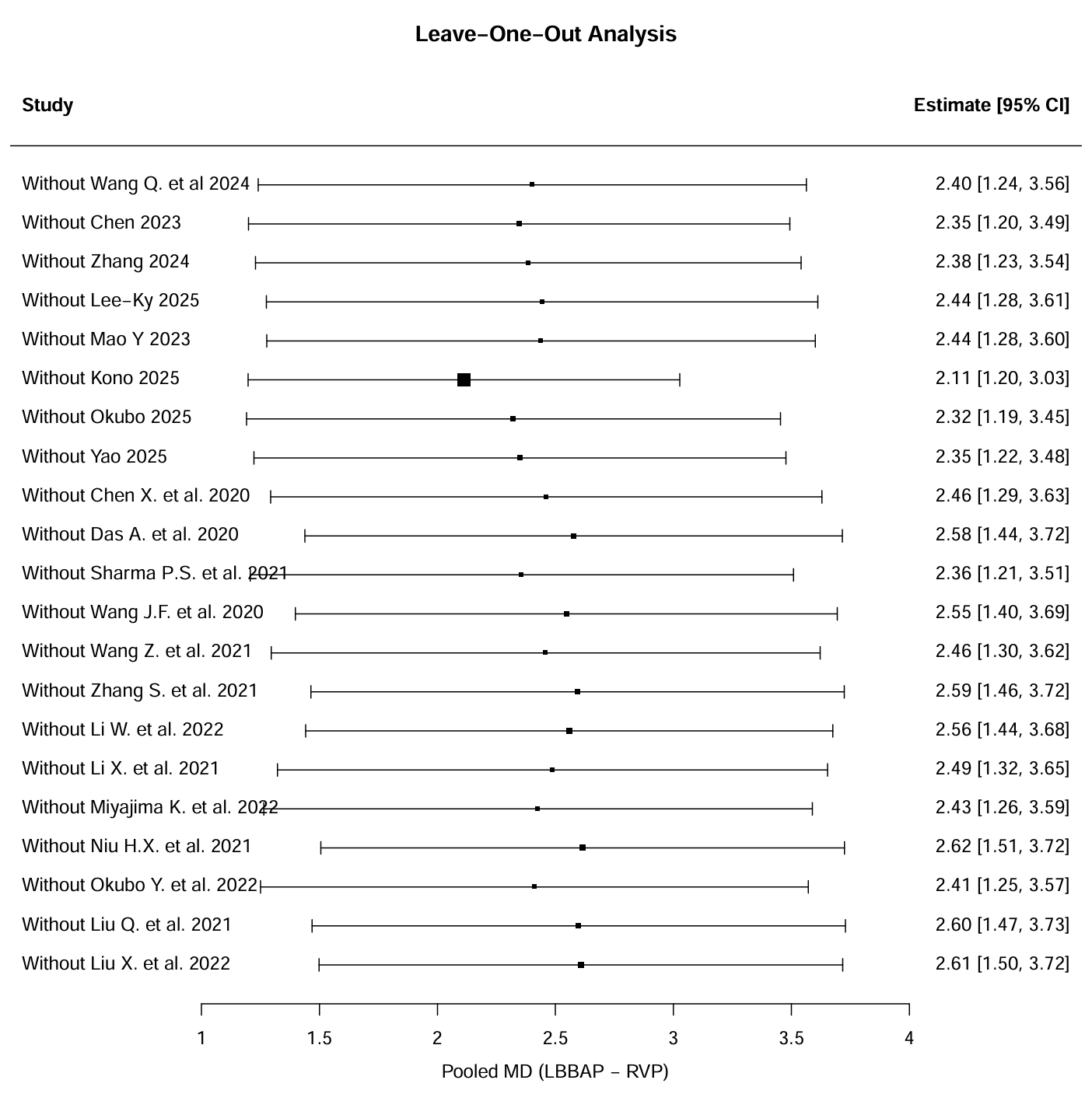
J.
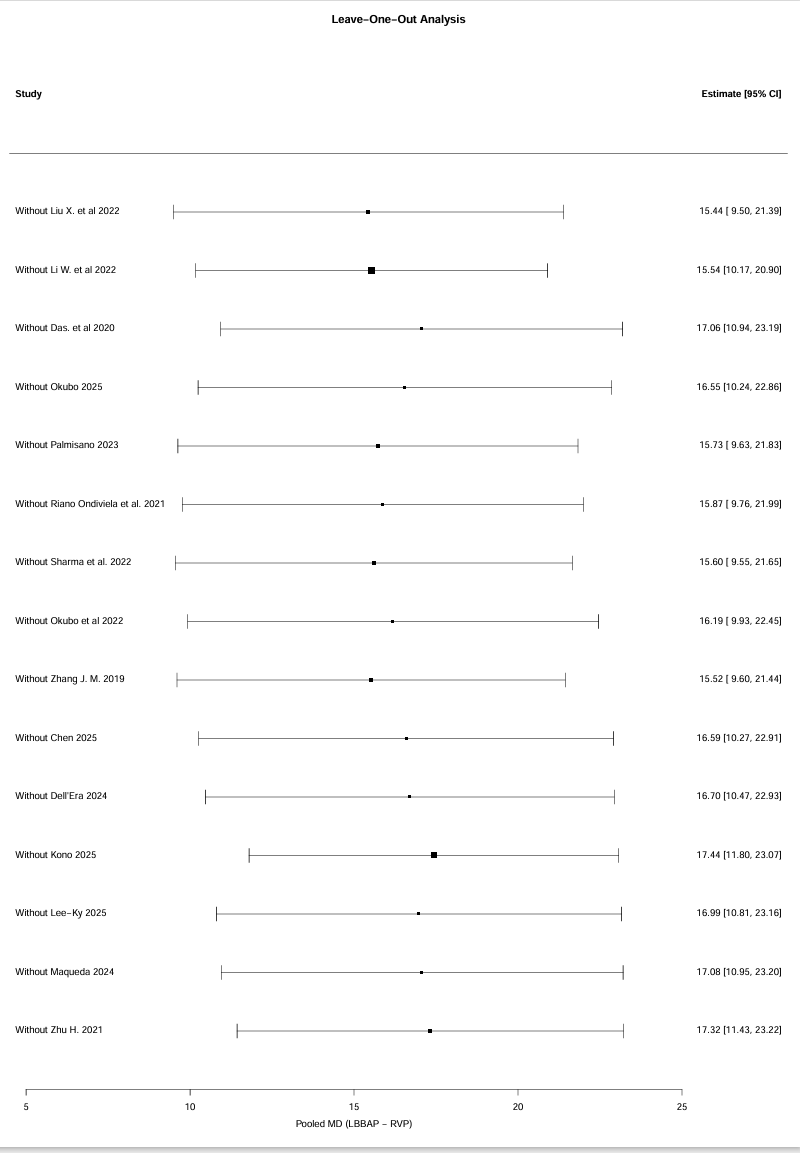
**

**K.
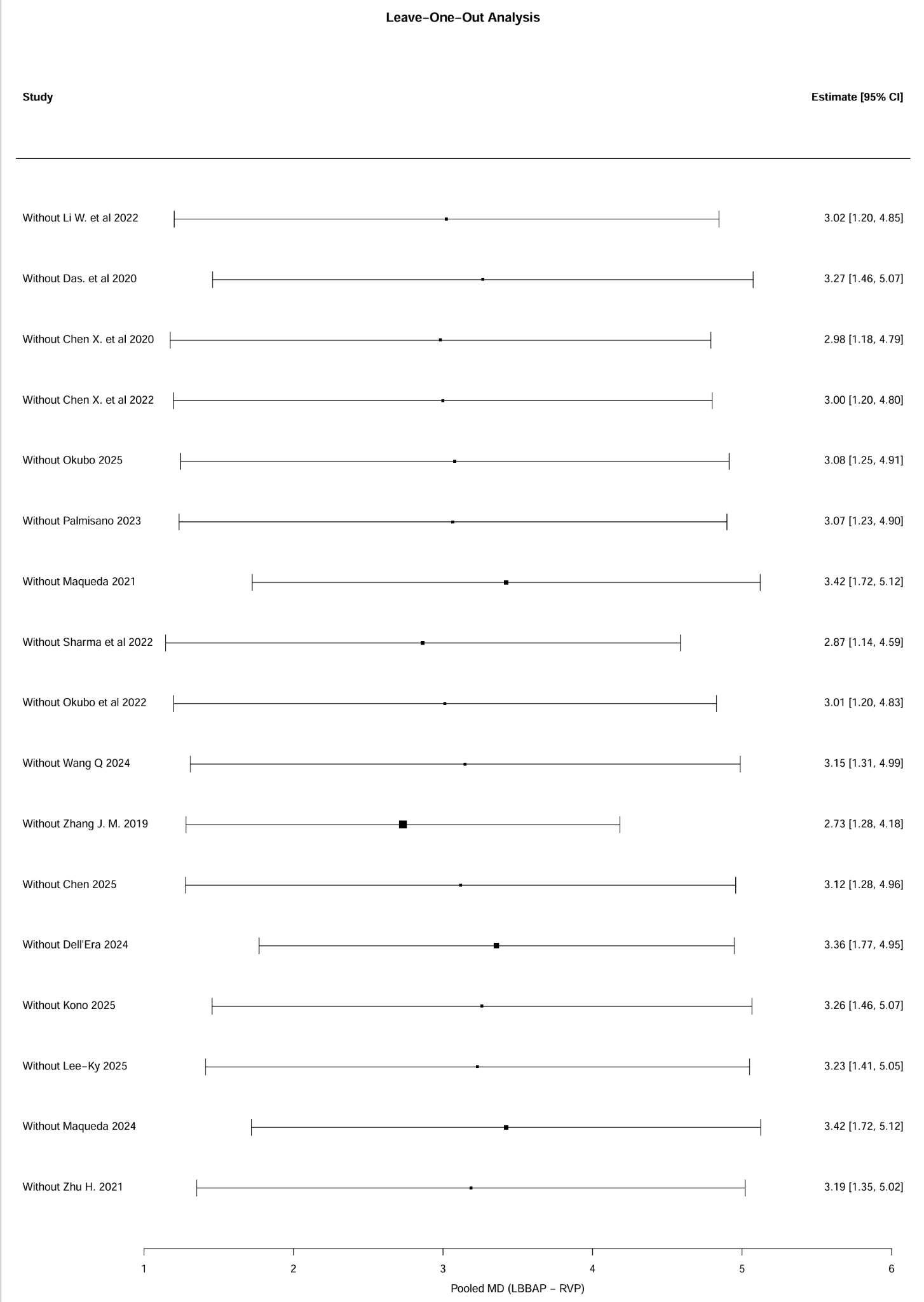
**

**L.**

**
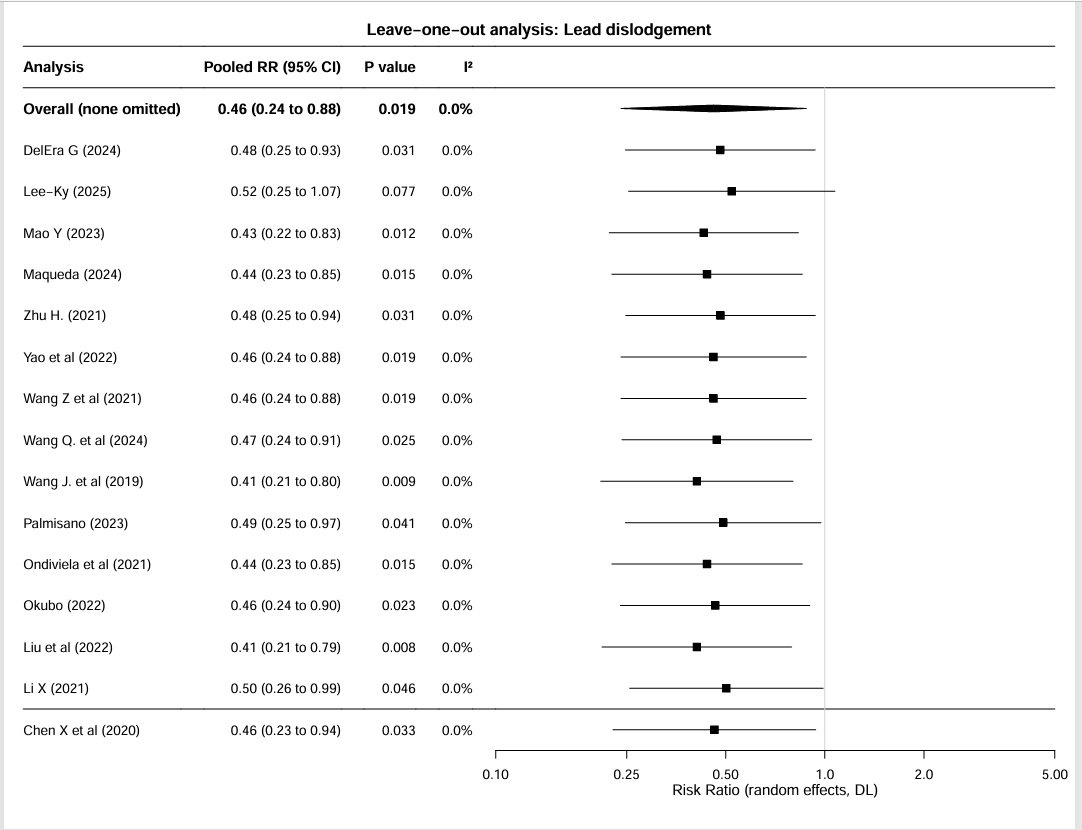
**

**M.**

**
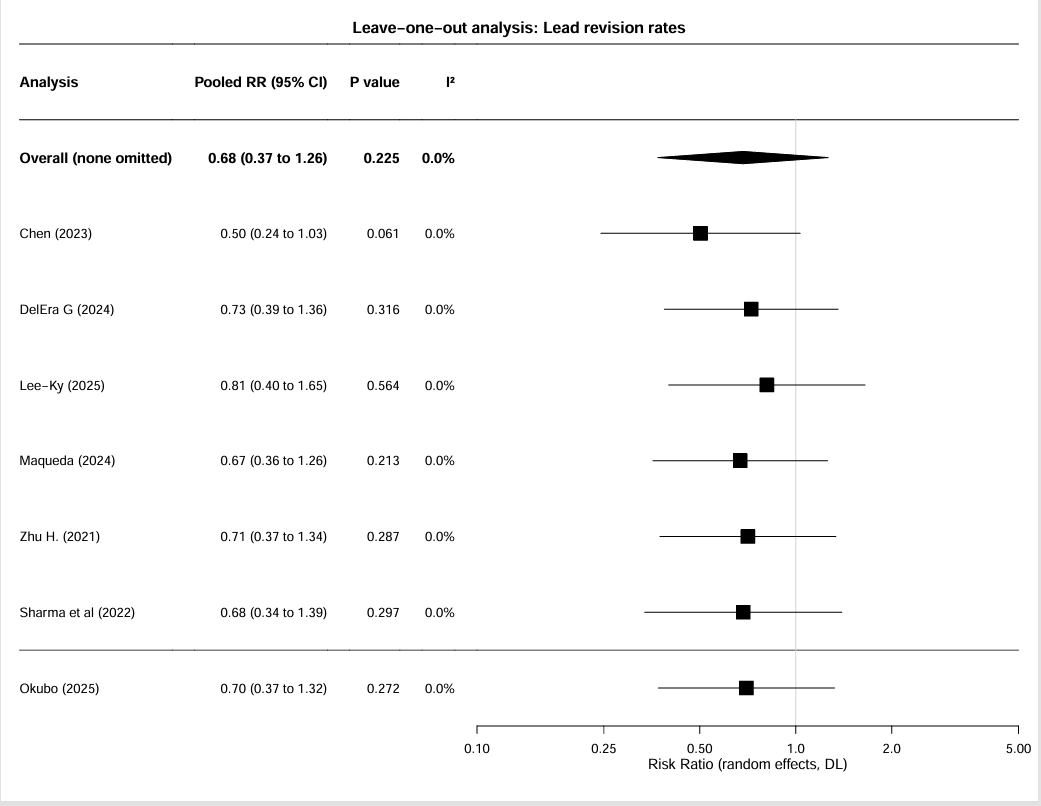
**

**N.**

**
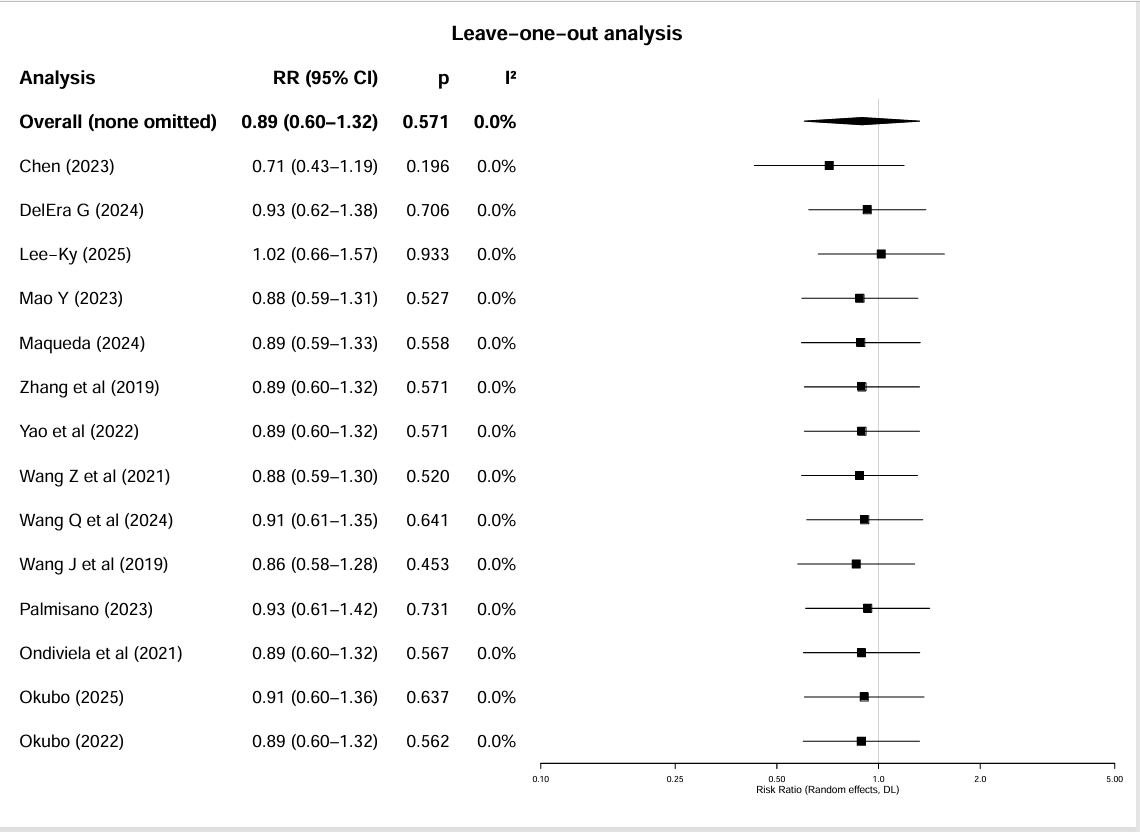
**

**O.**

**
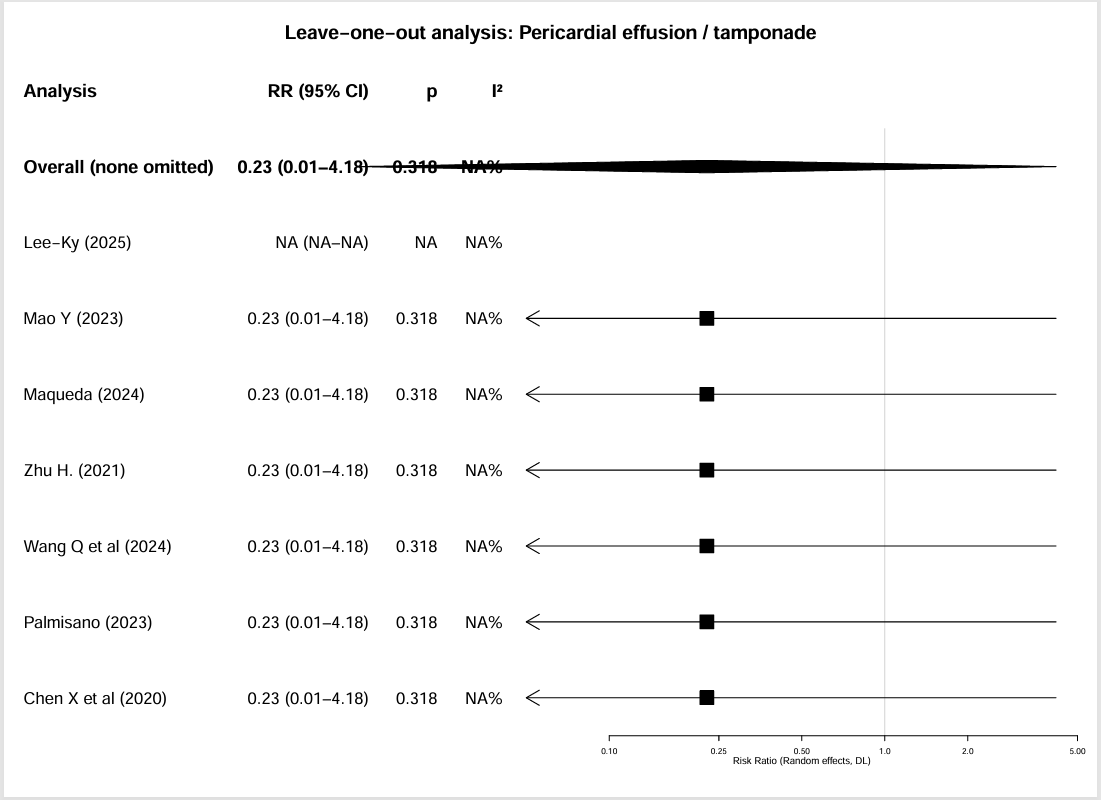
**

**P.**

**
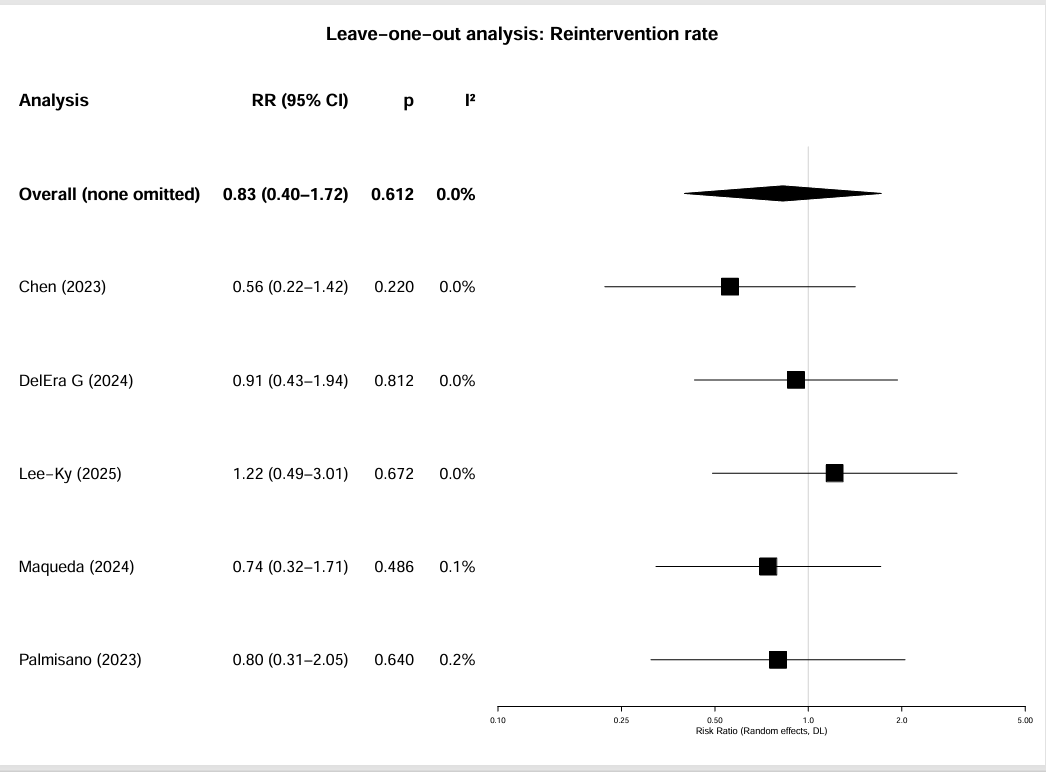
**

**Q.**

**Not applicable.**

**R.**

**
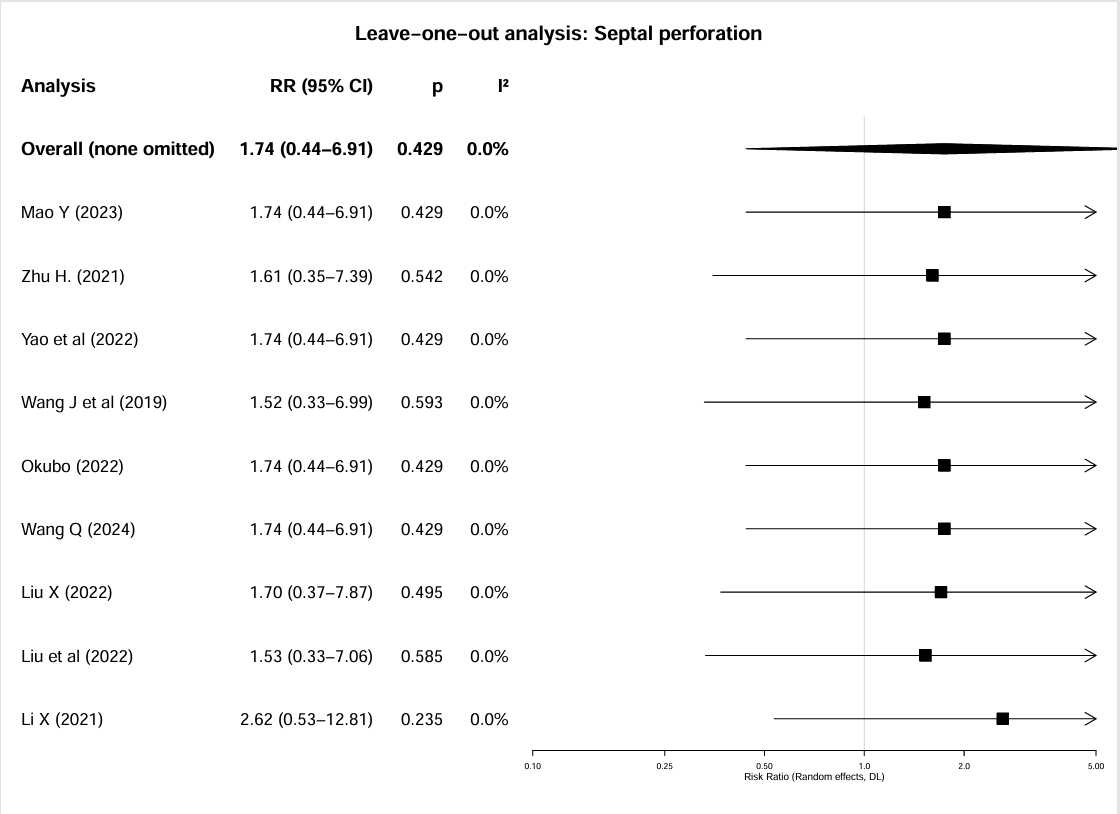
**

**S.**

**
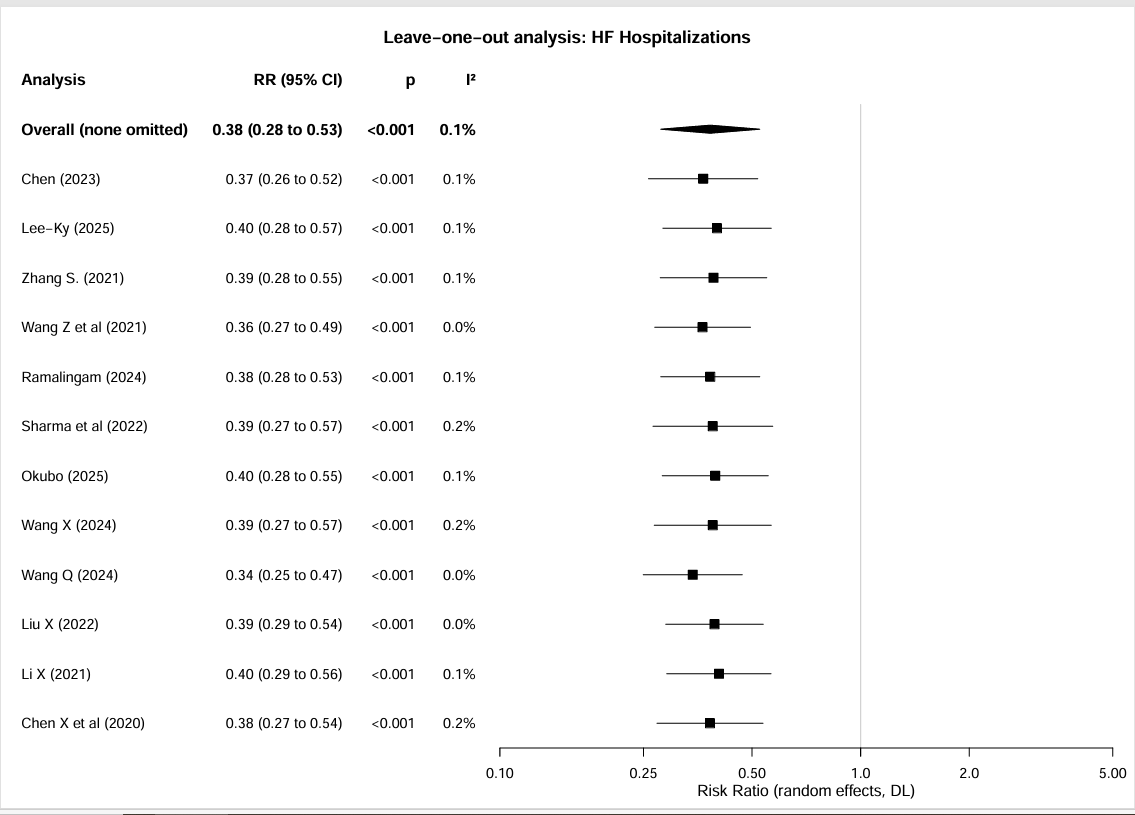
**

**T.**

**
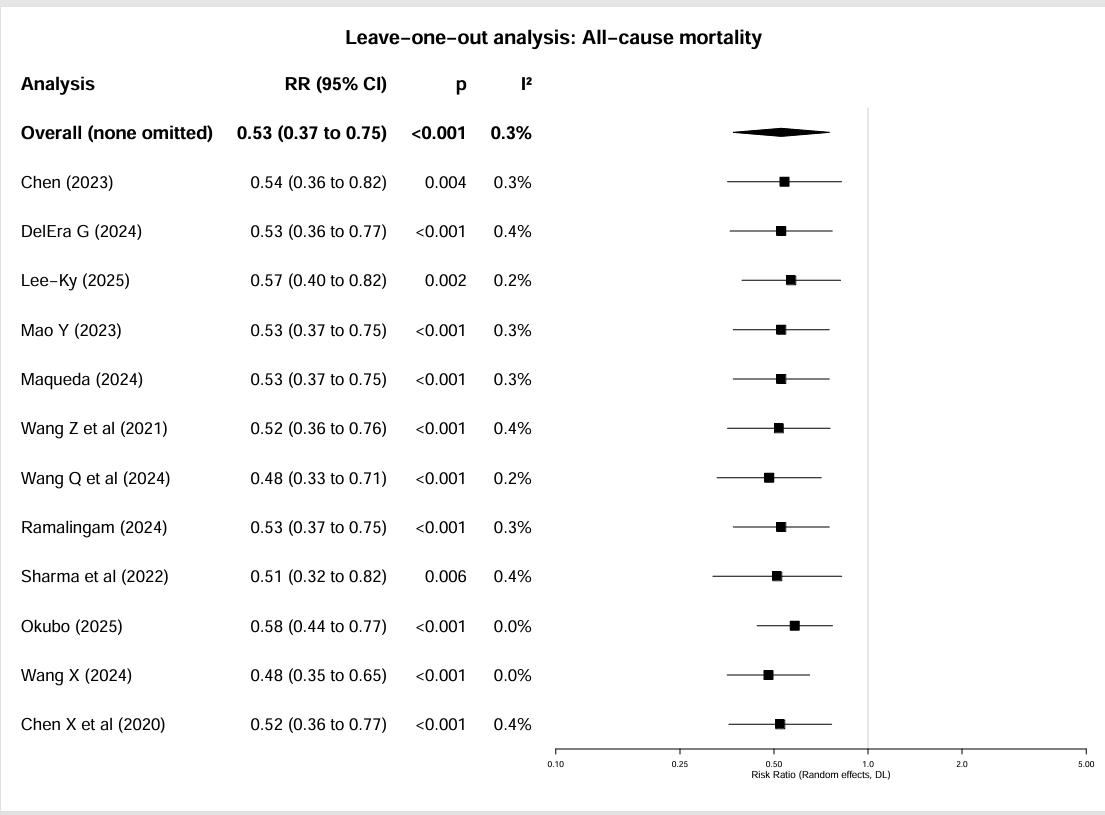
**

**U.**

**
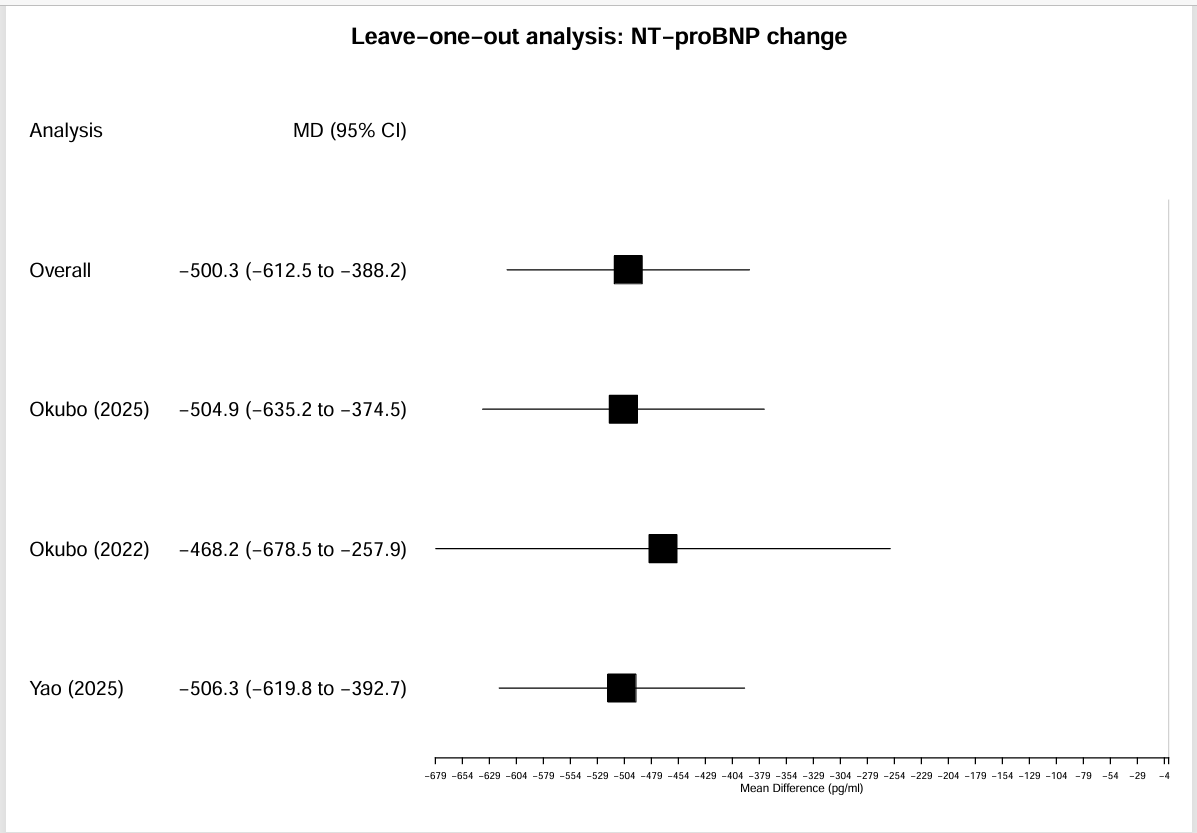
**

**FUNNEL PLOTS:**

**A.
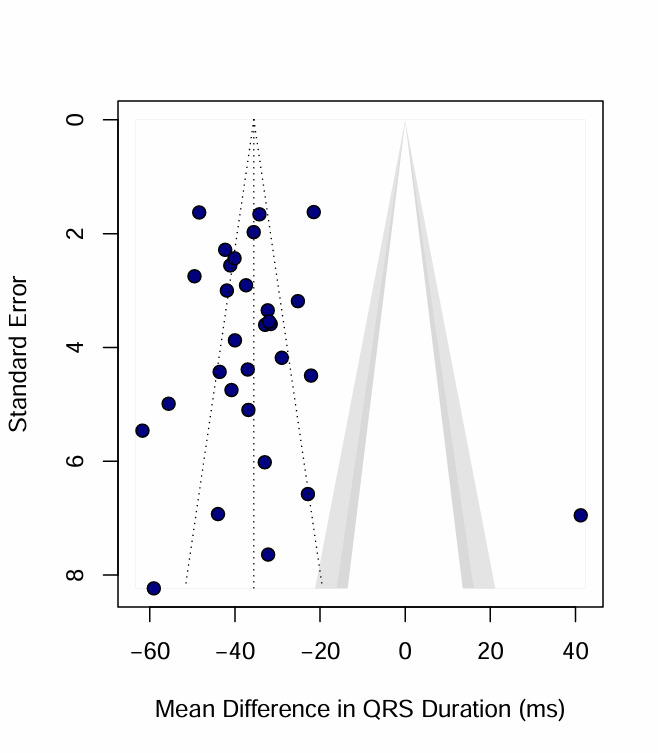
**

**B.
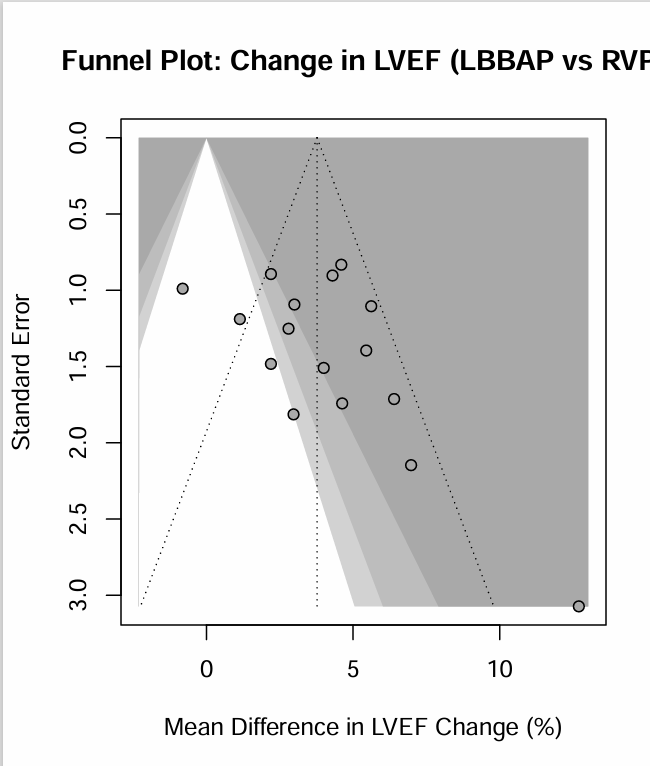
**

**C.
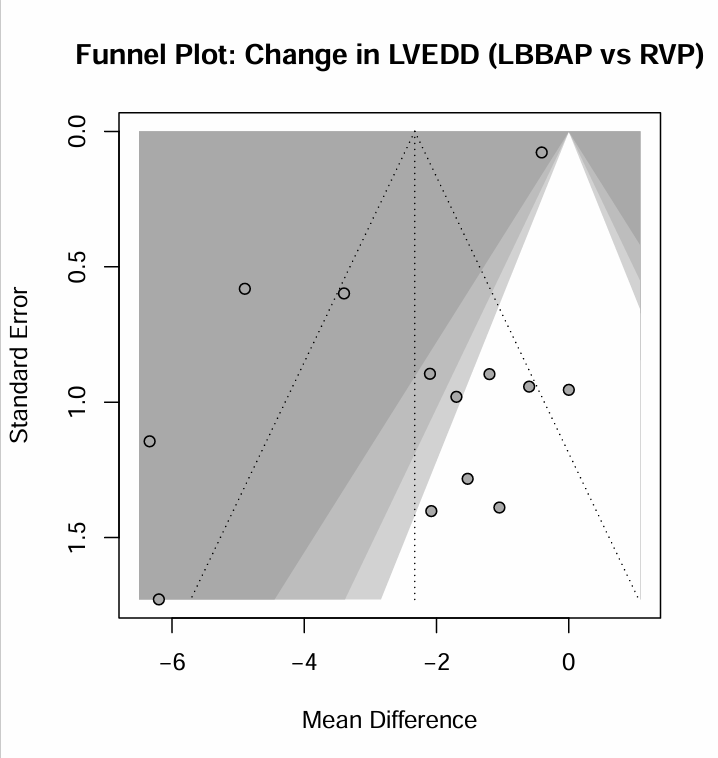
D.
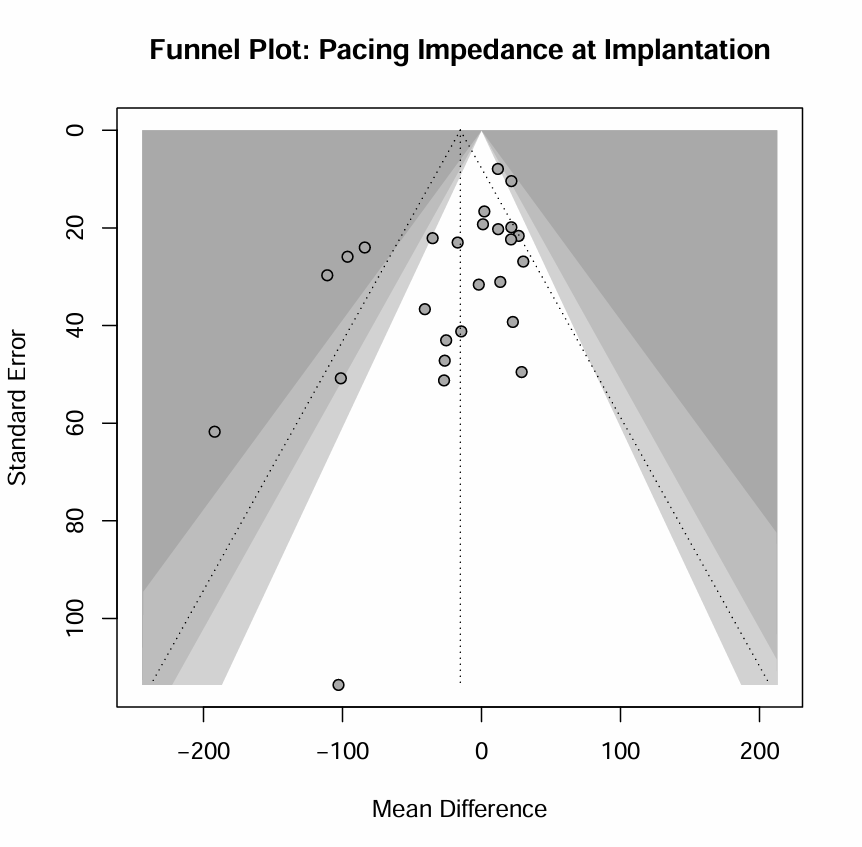
E.
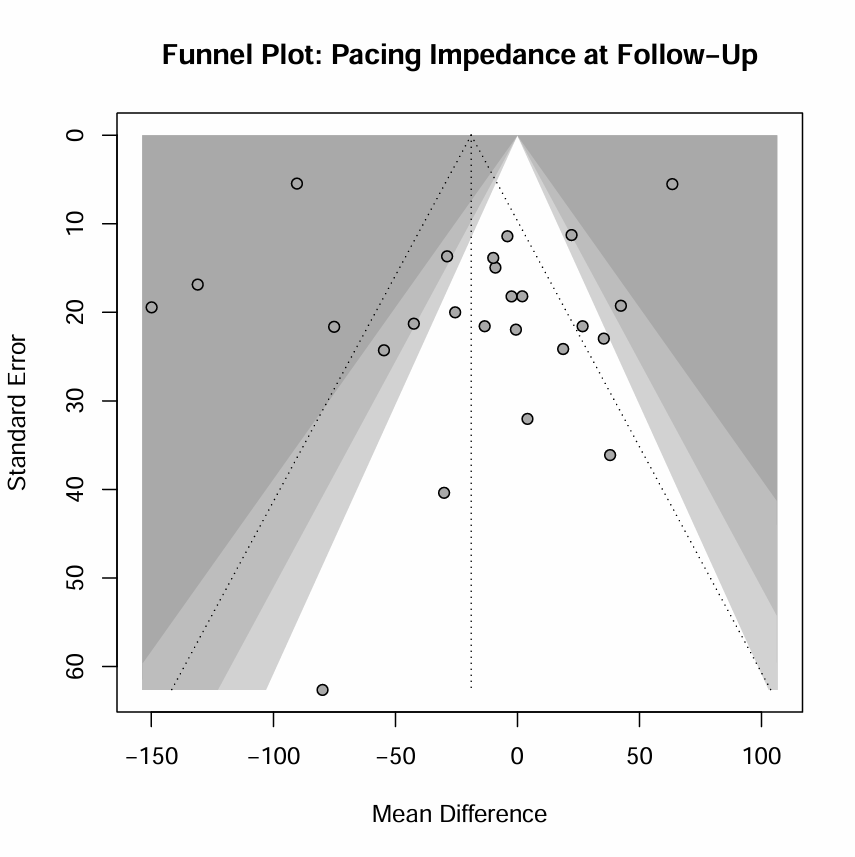
**

**F.
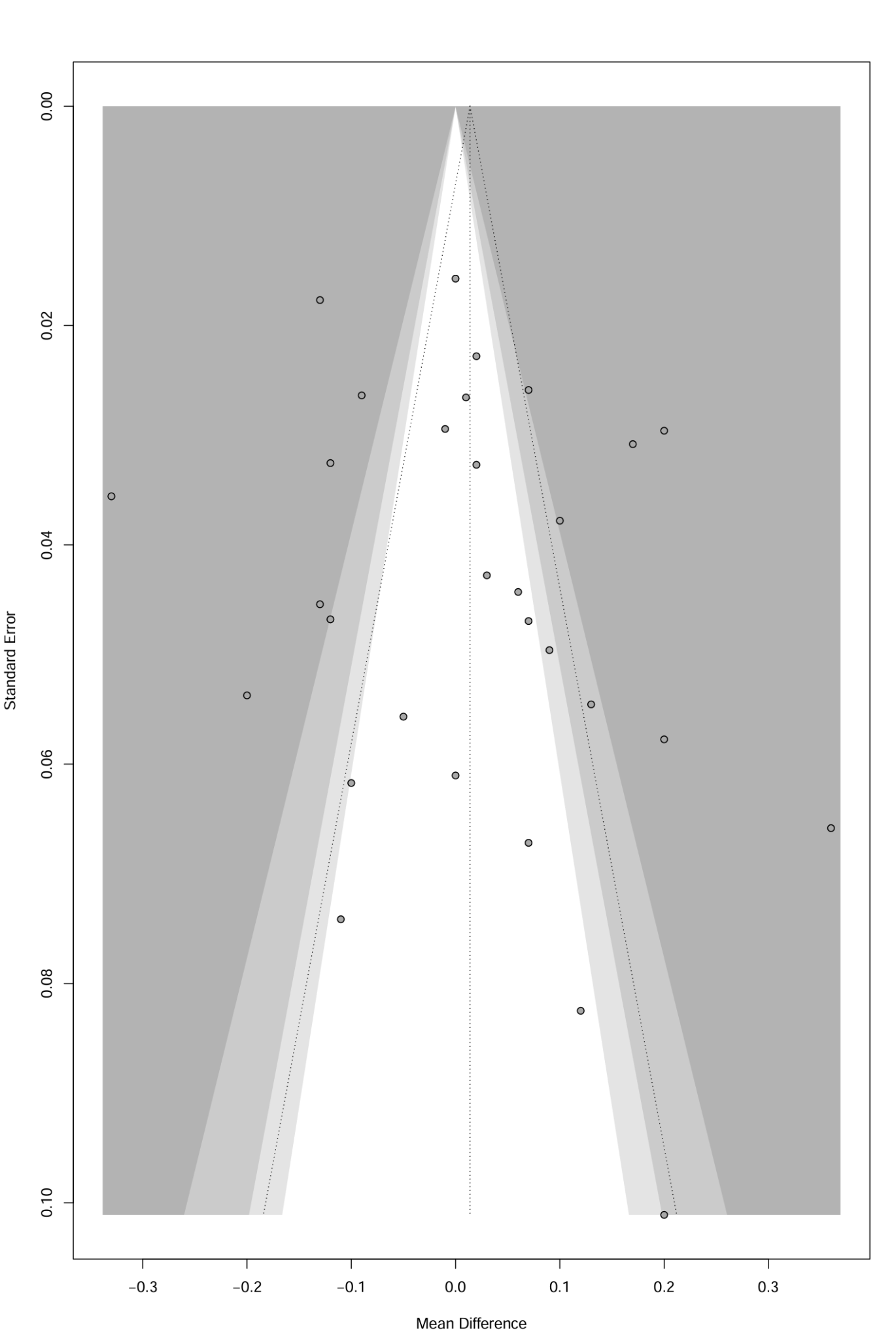
**

**G.
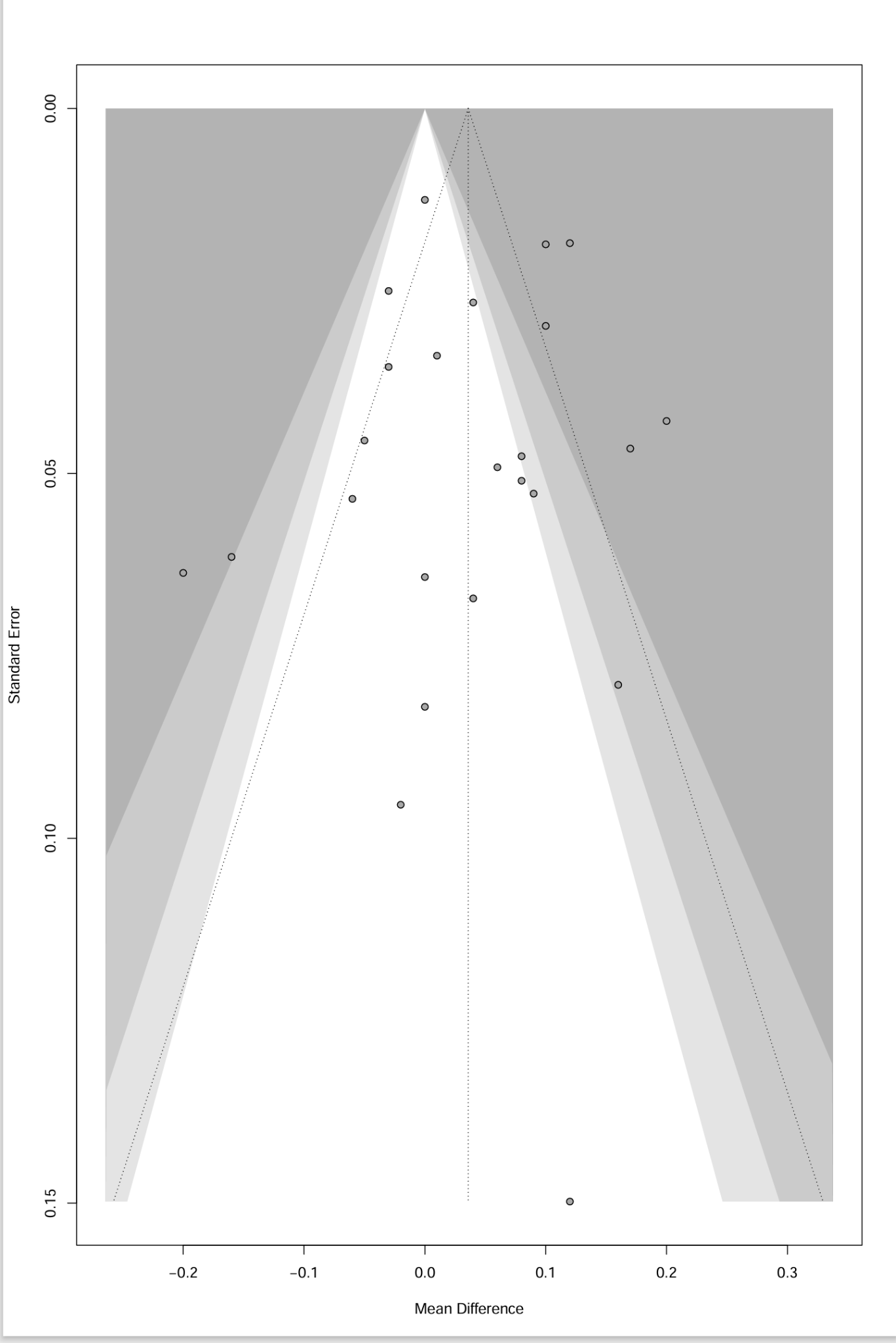
**

**H.
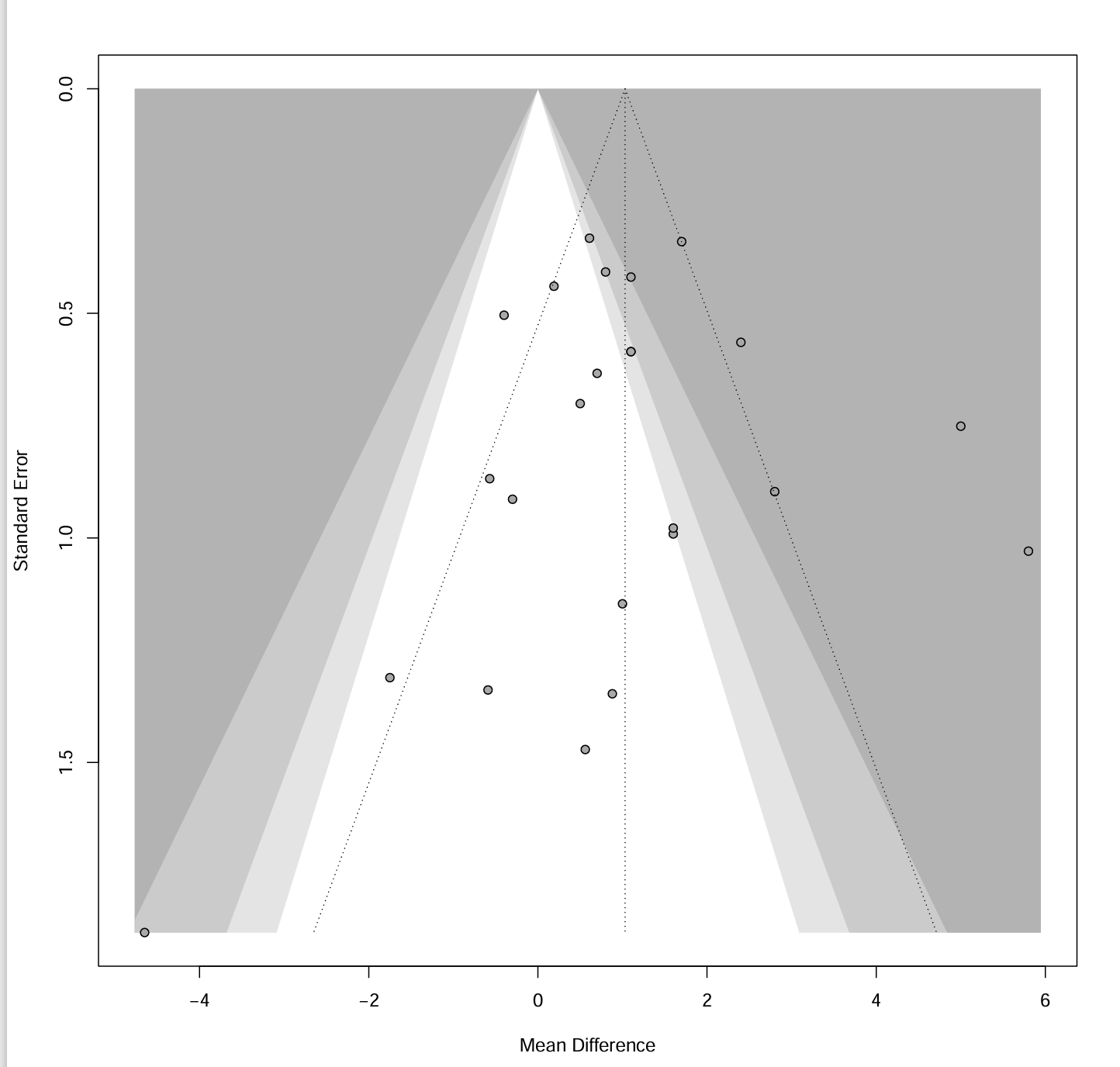
**

**I.
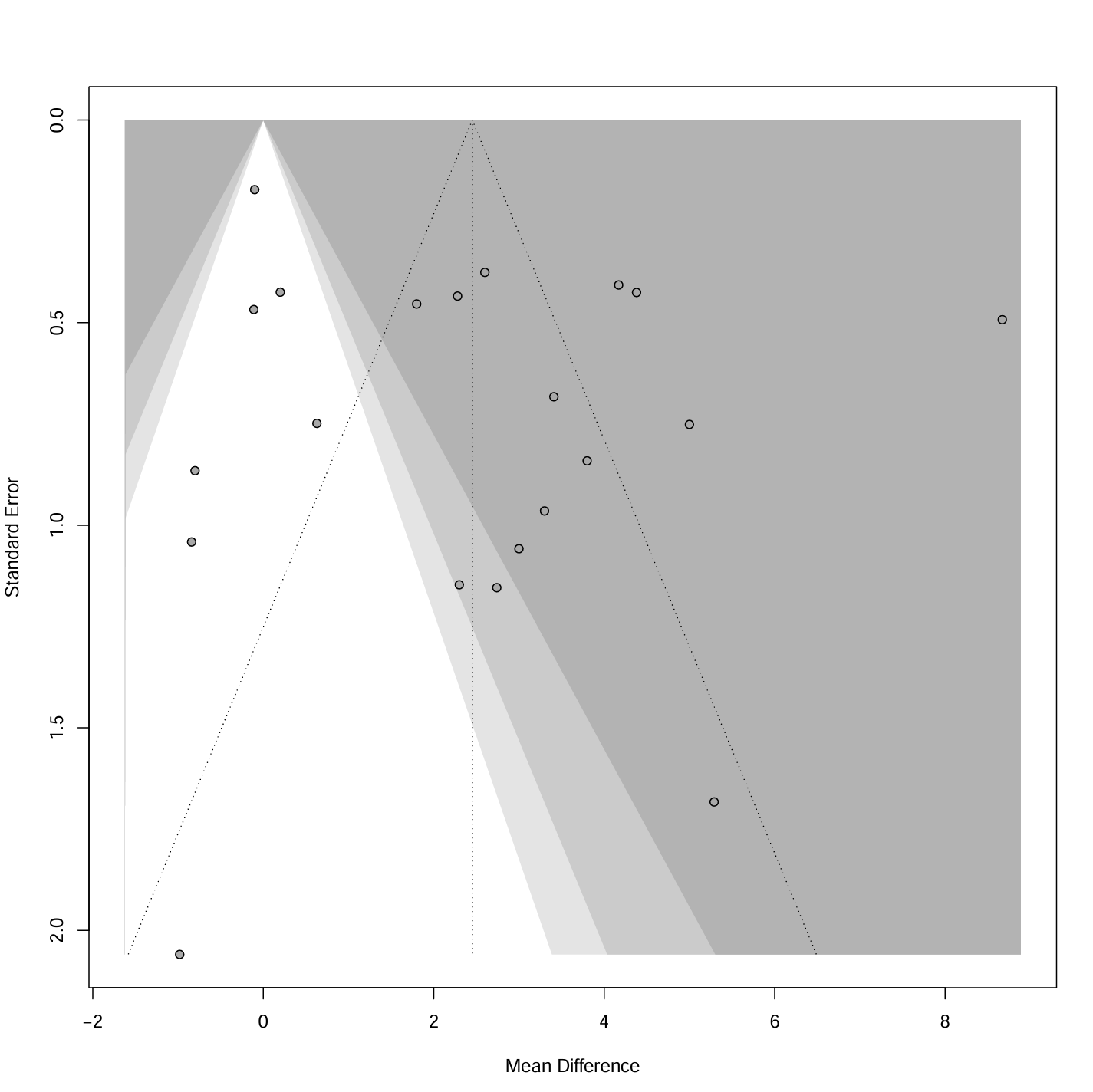
**

**J.
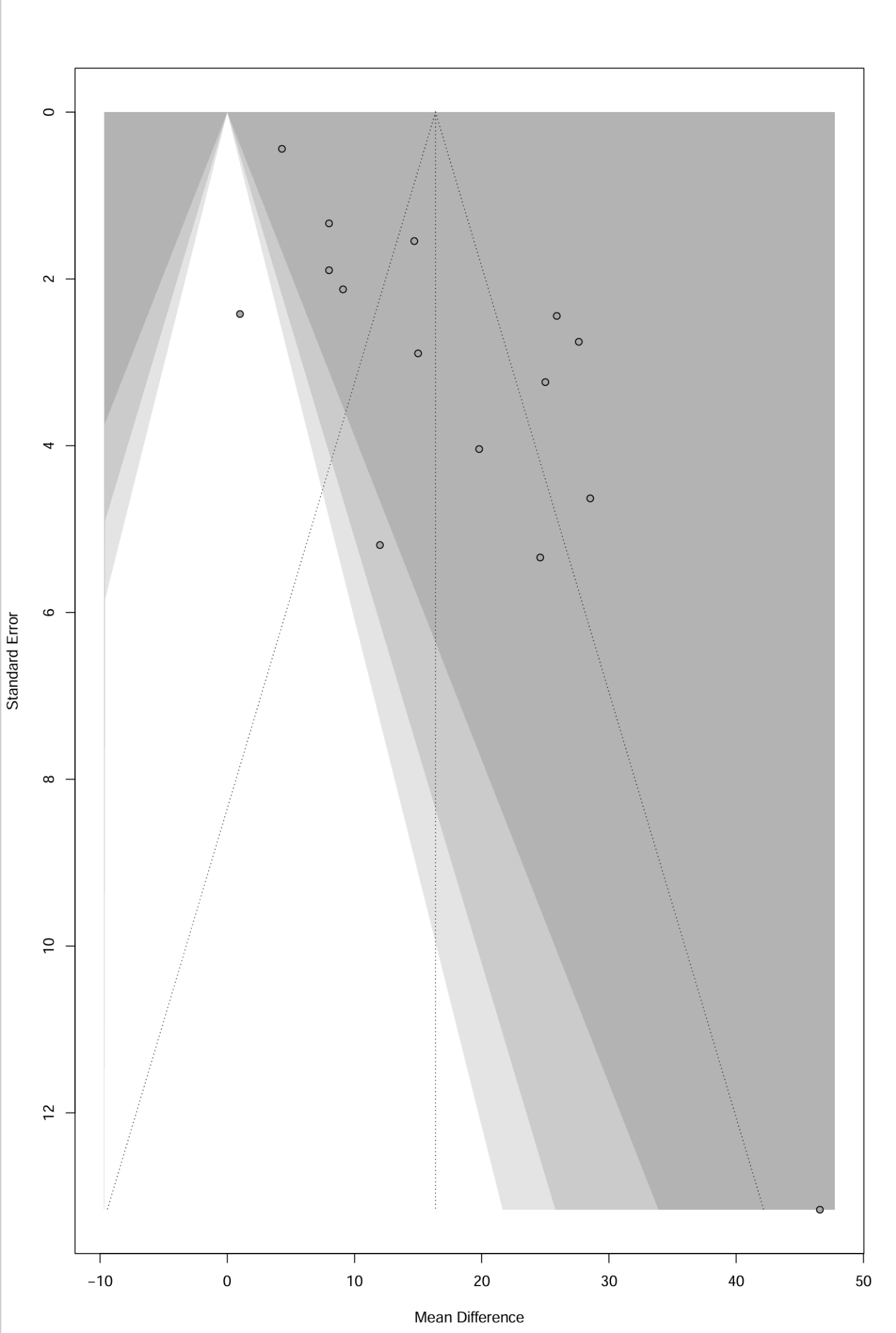
**

**K.
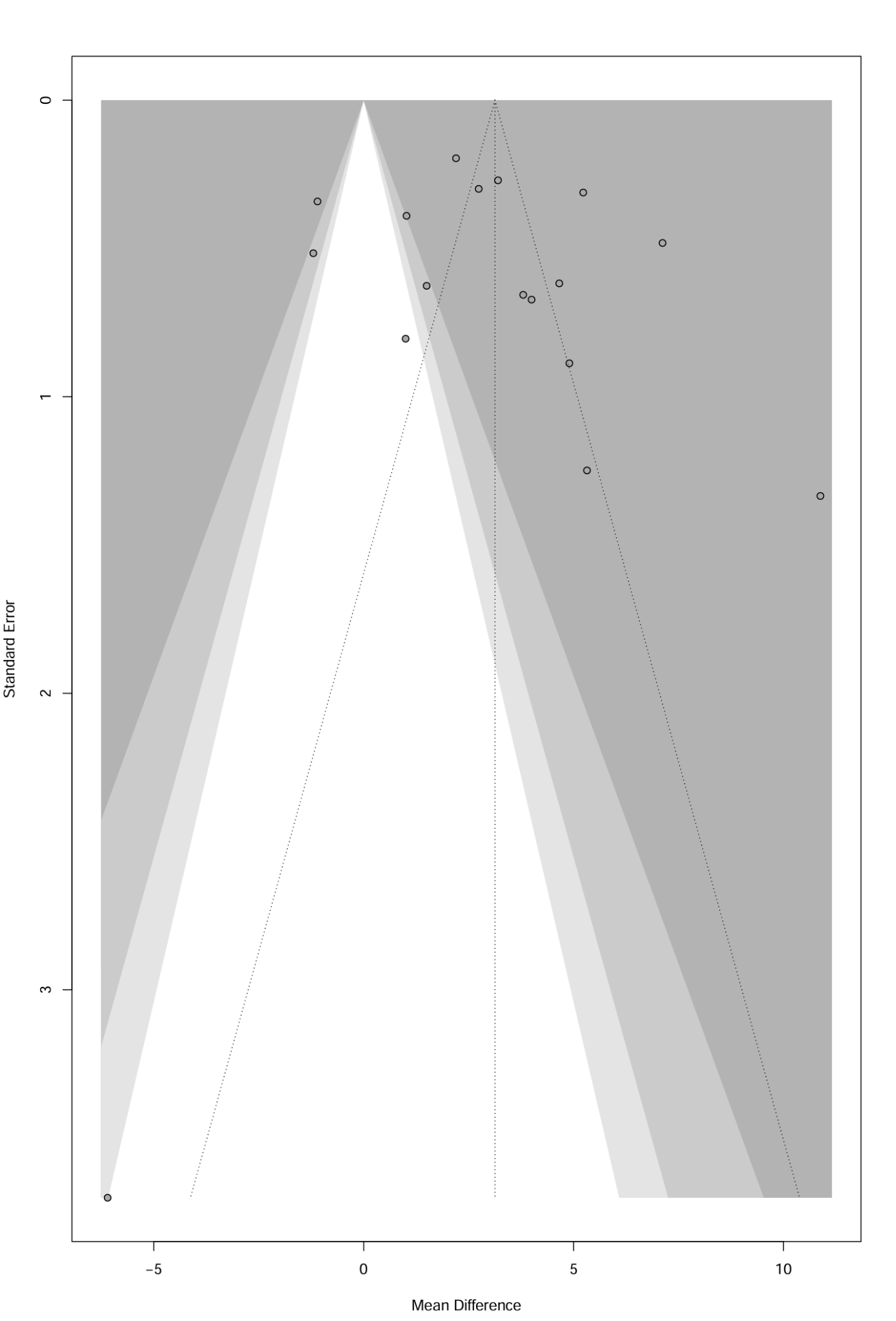
**

**L.**

**
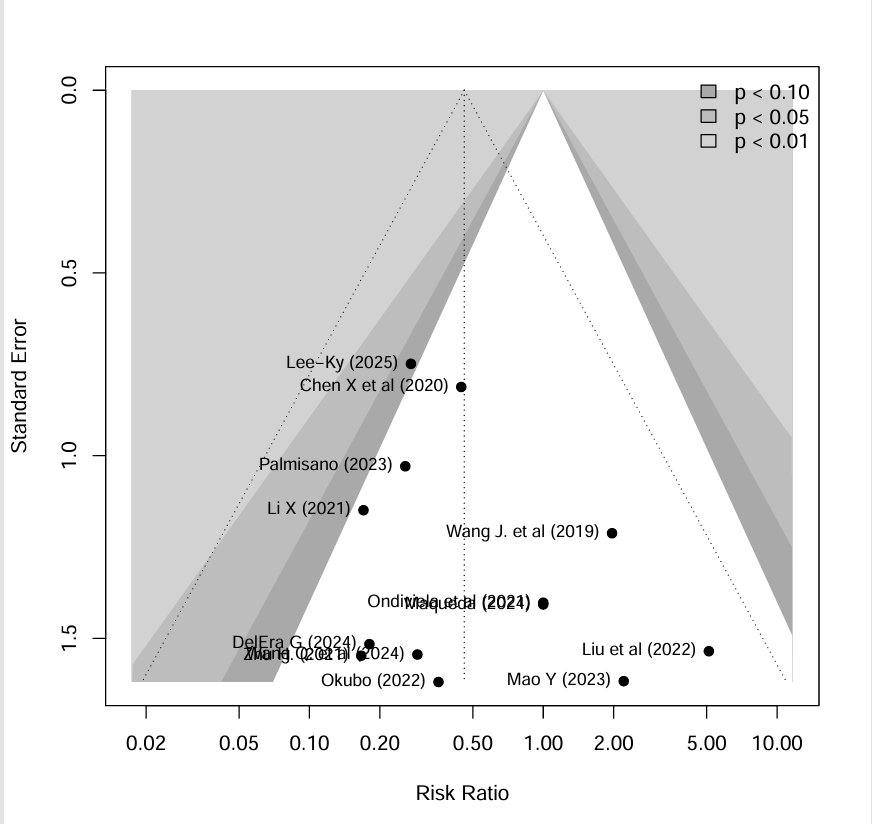
**

**M.**

**
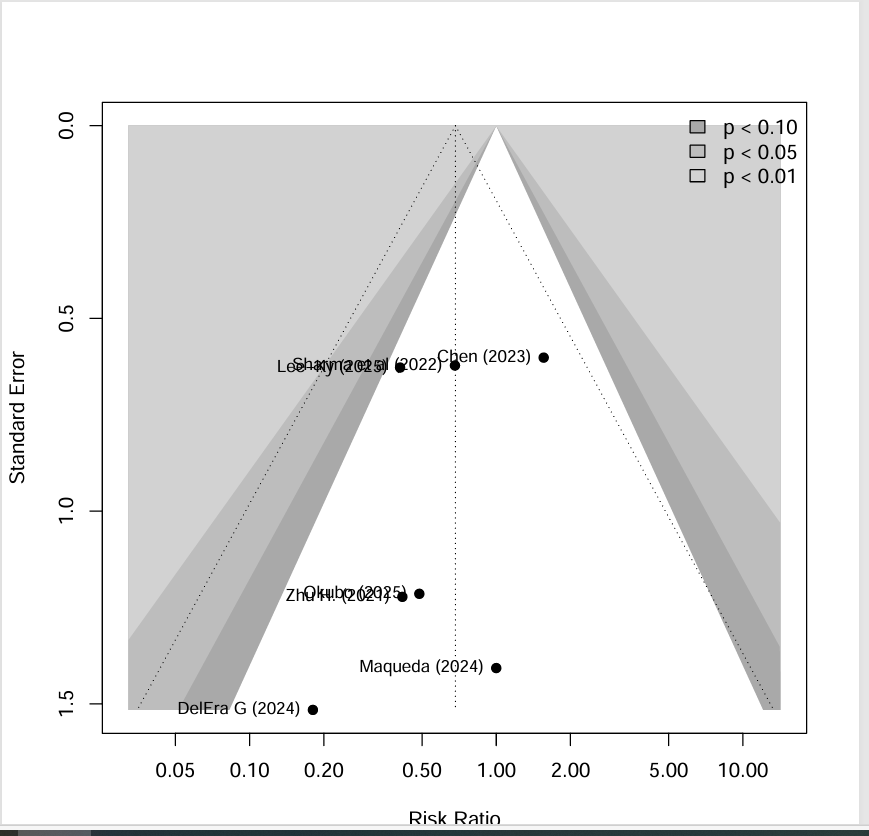
**

**N.**

**
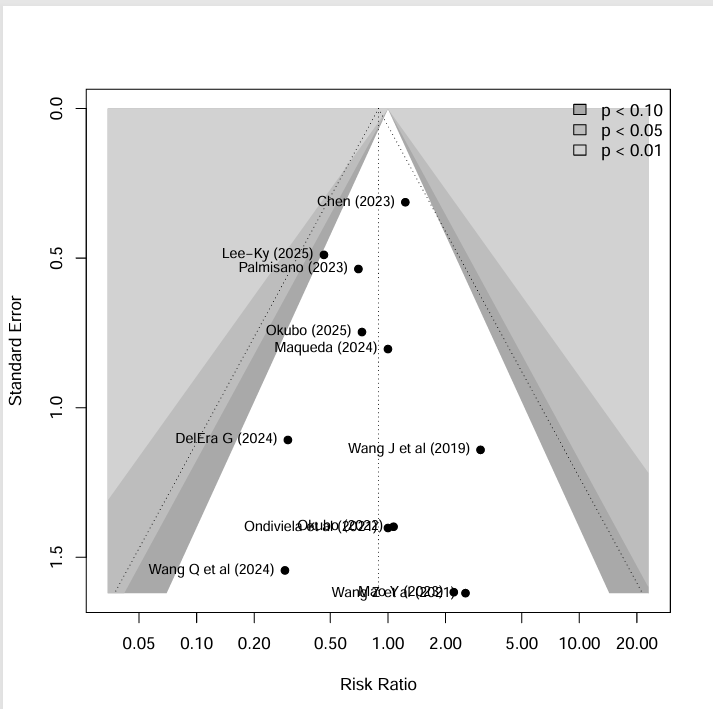
**

**O.**

**Less than 10 studies, that’s why no funnel plot was assessed or egger’s test was performed.**

**P.**

**Less than 10 studies, that’s why no funnel plot was assessed or egger’s test was performed.**

**Q.**

**Less than 10 studies, that’s why no funnel plot was assessed or egger’s test was performed.**

**R.**

**Less than 10 studies, that’s why no funnel plot was assessed or egger’s test was performed.**

**S.**

**
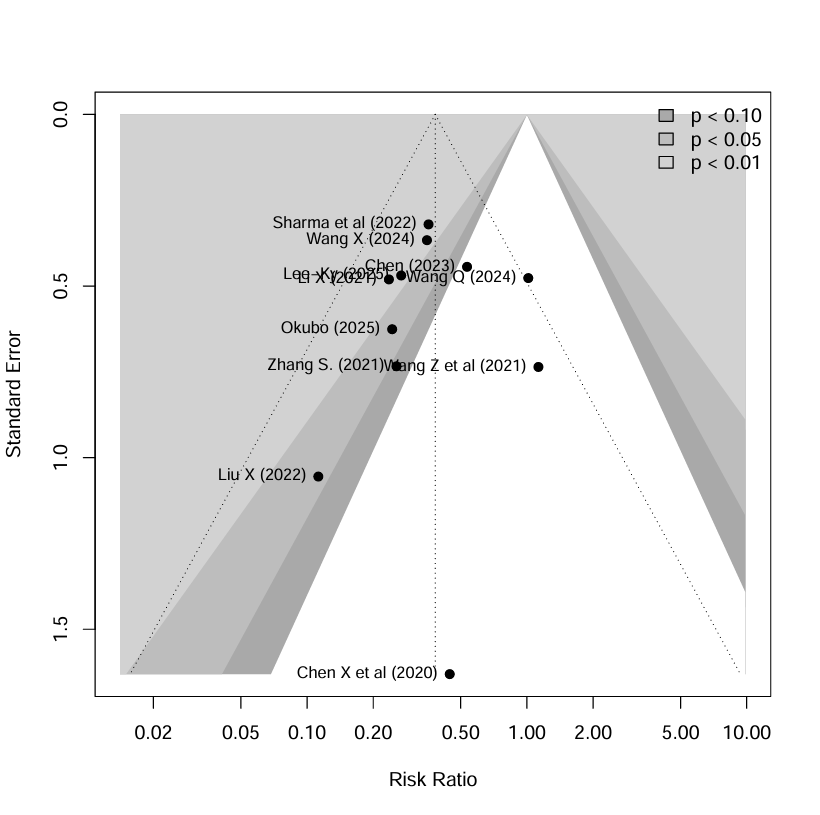
**

**T.**

**
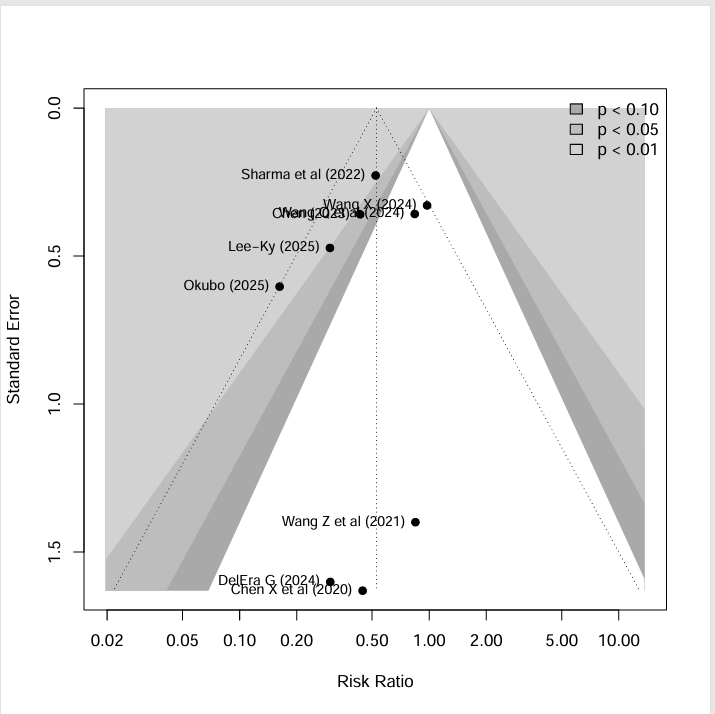
**

**U.**

**Less than 10 studies, that’s why no funnel plot was assessed or egger’s test was performed.**

**Egger’s Tests Results:**

1. **Change in QRS duration: p = 0.7828**
2. **Change in LVEF: p = 0.0946**
3. **Change in LVEDD: p = 0.0132**
4. **Pacing impedance at time of implantation: p = 0.0077**
5. **Pacing impedance at time of follow-up: p = 0.8164**
6. **Pacing threshold at time of implantation: p = 0.2924**
7. **Pacing threshold at time of follow-up: p = 0.8033**
8. **R wave amplitude at time of implantation: p = 0.9243**
9. **R wave amplitude at time of follow-up: p = 0.0992**
10. **Procedural time: p = 0.0005**
11. **Flouroscopic time: p = 0.5483**
12. **Lead dislodgement: p = 0.2471**
13. **Lead revision rates: Number of studies < 10 (so egger’s test not performed).**
14. **Overall complications rate: p-value = 0.9414**
15. **Pericardial effusion/tamponade: less than 10 studies ( so, no egger’s test was performed).**
16. **Reintervention rates: less than 10 studies ( so, no egger’s test was performed).**
17. **Periprocedural mortality: less than 10 studies ( so, no egger’s test was performed).**
18. **Septal perforation: less than 10 studies ( so, no egger’s test was performed).**
19. **HF hospitalisations: p-value = 0.8286**
20. **All-cause mortality: p-value = 0.7862**
21. **NT-pro BNP change: Less than 10 studies, that’s why no funnel plot was assessed or egger’s test was performed.**

**S6: Detailed baseline characteristics table of the included studies:**

| **Baseline characteristics table of all the included studies with demographics and clinical characteristics.** | | | | | | | | | |
| --- | --- | --- | --- | --- | --- | --- | --- | --- | --- |
|  |  |  |  |  |  |  |  |  |  |
| **Author** | **Year of Publication** | **Sample Sizes** | | **Study Design** | **Males (N or %)** | | **Age (mean +- SD)** | | **Follow-up** |
|  |  |  |  |  |  |  |  |  |  |
|  |  | **LBBAP** | **RVP** |  | **LBBAP** | **RVP** | **LBBAP** | **RVP** |  |
| Wang X | 2024 | 120 | 117 | multicenter, retrospective, observational study | 78 (65%) | 73 (62%) | 74.3 +- 6.6 | 74.7 +- 6.5 | 48.5 (34.9–60) months |
| Zhao | 2023 | 36 | 36 | Single-center, prospective, randomized controlled trial (RCT) | 20 (55.56%) | 18 (50.00%) | 64.26 ± 14.10 | 68.11 ± 10.04 | 6 months |
| Zhang | 2024 | 43 | 43 | Prospective observational study | 28 (65.1) | 26 (60.5) | 75.0 +- 10.6 | 72.4 +- 10.0 | 14.1 ± 7.5 months |
| Yao | 2025 | 30 | 30 | retrospective, observational cohort study | 20 (66.7%) | 20 (66.7) | 70.43 ± 12.68 | 74.00 ± 9.58 | 12 months |
| Liu X. et al | 2022 | 33 | 21 | Prospective observational | 21 | 11 | 73.67±11.87 | 68.14±11.66 | 13.80 ± 4.47 months. |
| Li Q. et al | 2021 | 42 | 42 | Prospective, controlled trial | 20 | 10 | 65.36 ± 13.08 | 68.19 ± 9.52 | 7 days |
| Liu et al. | 2022 | 45 | 46 | Observational | 26 | 18 | 74 +/- 9.2 | 70.5 +- 11.7 | LBBAP = 14 +- 6.1 months, RVSP = 13.3 +- 6.1 months |
| Li X. et al | 2021 | 235 | 120 | Prospective observational study | 161 | 81 | 63.3 ± 15.5 | 62.1 ± 17.2 | 11.4 ± 2.7 months |
| Li W | 2022 | 30 | 38 | retrospective cohort study | 12 | 26 | 70.23 ± 9.58 | RVAP=73.13 ± 6.62 | 12 months |
| Das. et al | 2020 | 22 | 28 | RCT | NR | NR | 63.36 +/- 7.82 | 61.64 +/- 5.90 | 6 months |
| Chen X. et al | 2020 | 237 | 317 | Prospective , non-randomized | 130 | 157 | 67.76 ± 13.29 | 69.15 ± 11.48 | 18.13± 1.77 months for LBBP, 18.37 ± 2.13 months for RVP |
| Chen K. et al | 2022 | Total 20 | NR | Single-center, Self-controlled Observational study | In total: 15 (75%) | In total: 15 (75%) | 66.15 ± 13.65 | 66.15 ± 13.65 | 18 months |
| Chen K. et al | 2018 | 20 | 10 | Prospecitve | 7 | 9 | 66.90 +/- 7.49 | 71.65 +/- 7.80 | 3 months |
| Cai B. et al | 2020 | 20 | 17 | Case Control? | 14 | 9 | 84 +/- 6 | 83 +/- 7 |  |
| Byeon K | 2022 | 42 | 84 | Registry | 22 (52) | 45 (54) | 71 ± 16 | 69 ± 15 | 6.8 ± 4.8 months |
| Okubo 2025 | 2025 | 81 | 79 | Single-center, prospective, non‑randomized controlled observational study | 40 (49.4) | 47 (59.5) | 76.9±11.7 | 77.4±10.3 | 12 months |
| Palmisano | 2023 | 73 | 201 | Analysis of multicentre registry | 48 | 120 | 79.2±9.8 | 79.3 6 ±10.0 | 18 months |
| Ramalingam et al | 2024 | 50 | 50 | Cohort | 19 | 25 | 63 (14) | 64 (13) | 6 months |
| Wang Q | 2024 | 109 | 158 | prospective observational study | 54 | 66 | 80.7 ± 4.1 | 80.8 ± 4.0 | LBBAP= 35.2 ± 15.6 months  RVP= 28.0 ± 17.1 months |
| Chen | 2023 | 393 | 510 | Retrospective cohort study | 240 (61.07%) | 311 (60.98%) | 71.7 ± 11.7 years | 73.0 ± 13.2 years | 4 years |
| Chen | 2025 | 122 | 166 | retrospective cohort | 73 (59.84%) | 89 (53.61%) | 64.5 ± 13.2 | 67.3 ± 11.7 | 24 ± 6 months |
| Dell'Era | 2024 | 20 | 18 | prospective, multicenter, observational, comparative study. | 14 (70%) | 9 (50%) | 83 ± 7 years | 84 ± 6 years | 4.2 ± 2.8 months. |
| Kono | 2025 | 75 | 296 | Retrospective, single-center cohort study | 39 (52.0%) | 160 (54.1%) | 78.0±11.1 years | 78.0±8.9 years | 2.9 years (IQR 2.0 to 3.6 years) |
| Lee-Ky | 2025 | 243 | 495 | observational cohort study | 130 (53.5%) | 251 (50.7%) | 71.5 ± 13.4 | 72.5 ± 11.6 | 1 year median follow-up |
| Mao | 2024 | 31 | 29 | Non-randomized, observational study | 18 (58%) | 23 (79%) | 71 ± 10 years | 75 ± 9 years | 15 ± 9 months |
| Mao Y | 2023 | 45 | 33 | Non-randomized, prospective, observational study. | 25 (55.6%) | 21 (63.6%) | 72.7 ± 12.2 | 72.9 ± 11.8 | 1 year |
| Maqueda | 2024 | 100 | 100 | Non-randomized, single-center, observational study | 60 (60%) | 67 (67%) | 77.3 ± 8.0 years | 78.7 ± 6.7 years | 6 months |
| Zhang S. | 2021 | 29 | 37 | retrospective observational study | 13(44.83%) | 17(45.95%) | 63.60 ± 8.80 | 67.40 ± 8.81 | 2 year max follow up, 12-24 months |
| Zhu H. | 2021 | 406 | 313 | single-center prospective observational study. | 197 (48.5%) | 150 (47.9%) | 64.9 ± 14.3 | 67.5 ± 12.2 | 13.6 ± 7.8 months |
| Zhu H. | 2023 | 257 | 270 | prospective observational cohort study | 119 (46.3%) | 130 (48.1%) | 63.6± 13.5 | 66.9±11.5 | 11.1 months |
| Miyajima et al. | 2022 | 39 | 42 | Prospective | 20 | 24 | 78 ± 10 | 79 ± 11 | 3 months |
| Niu H.X.et al. | 2021 | 20 | 30 | Prospective single center study | NR | NR | NR | NR | 15.0 ± 9.1 months |
| Riano Ondiviela et al. | 2021 | 60 | 60 | RCT | 62 | 60 | 76.7 ± 9, | 79.7 ± 8 | 3 months |
| Sharma et al. | 2022 | 321 | 382 | Observational study | 168 | 200 | 75.33 ± 12.26 | 74.96 ± 11.85 | 18 months |
| Okubo et al | 2022 | 43 | 46 | single-center, prospective study | 20 | 25 | 77.4 ± 10.6 | 76.2 ± 10.9 | 6 months |
| Sun Z | 2020 | 16 | 16 | retrospective study. | 7 | 5 | 71.4 ± 14.4 | 73.6 ± 8.9 | 12 months |
| Wang J.F. et al. | 2019 | 66 | 65 | RCT | 38 | 41 | 71.12 ± 13.14 | 72.03 ± 12.11 | 6 months |
| Wang Z et al | 2021 | 52 | 44 | prospective observational study | 34 | 22 | 67.9 ± 12.6 | 67.2 ± 11.6 | 13.9 ± 7.0 months, |
| Yao L. et al. | 2022 | 25 | 25 | RCT | 13 | 14 | 66.3 ± 11.0 | 69.2 ± 12.8 | 18 months |
| Zhang J. M. | 2019 | 23 | 21 | prospective, randomized controlled trial (RCT) | 17 | 10 | 64.61 ± 12.65 | 65.76 ± 13.53 | 12 months |

| **Baseline characteristics table of all the included studies with demographics and clinical characteristics. (continued)** | | | | | | | | | |
| --- | --- | --- | --- | --- | --- | --- | --- | --- | --- |
| **Author** | **Year of Publication** | **LBBB n(%)** | | **RBBB n(%)** | | **Comorbidities** | | | |
|  |  |  |  |  |  | **Atrial fibrillation n(%)** | | **Hypertension n(%)** | |
|  |  | **LBBP** | **RVP** | **LBBP** | **RVP** | **LBBP** | **RVP** | **LBBP** | **RVP** |
| Wang X | 2024 | 3 (3%) | 5 (4%) | 8 (7%) | 9 (8%) | 23 (19%) | 20 (17%) | 67 (56%) | 59 (50%) |
| Zhao | 2023 | 1 (2.78%) | 0 (0.00%) | 6 (14.29%) | 2 (4.76%) | NR | NR | 25 (69.44%) | 21 (58.34%) |
| Zhang | 2024 | NR | NR | NR | NR | NR | NR | 36 (83.7) | 33 (76.7) |
| Yao | 2025 | 3 (10.0) | 4 (14.3) | 10 (33.3) | 13 (46.4) | 6 (20.0) | 6 (20.0) | 17 (56.7) | 20 (66.7) |
| Liu X. et al | 2022 | 5 | 2 | 0 | 3 | 6 | 6 | 19 | 12 |
| Li Q. et al | 2021 | NR | NR | NR | NR | 6 | 0 | 28 | 29 |
| Liu et al. | 2022 | 5 | 2 | 0 | 5 | 13 | 15 | 25 | 27 |
| Li X. et al | 2021 | NR | NR | NR | NR | 72 | 19 | 132 | 65 |
| Li W | 2022 | 9 | 2 | 5 | RVAP=12 | 7 | 2 | 20 | 29 |
| Das. et al | 2020 | NR | NR | NR | NR | NR | NR | NR | NR |
| Chen X. et al | 2020 | NR | NR | NR | NR | 35 | 31 | 102 | 162 |
| Chen K. et al | 2022 | In total: 2 (10%) | NR | In total: 9 (45%) | NR | NR | NR | In total: 10 (50%) | NR |
| Chen K. et al | 2018 | 2 | 0 | 1 | 2 | 5 | 4 | 13 | 14 |
| Cai B. et al | 2020 | NR | NR | NR | NR | 7 | 7 | 19 | 18 |
| Byeon K | 2022 | NR | NR | NR | NR | 9 | 13 | 20 | 43 |
| Okubo 2025 | 2025 | 16 (19.8%) | 16 (21.1%) | 21 (25.9%) | 14 (17.8%) | 29 (35.8%) | 29 (36.7%) | 63 (77.8%) | 58 (73.4%) |
| Palmisano | 2023 | 5 | NR | NR | NR | 16 | 19 | 56 | NR |
| Ramalingam et al | 2024 | 10 | 11 | 19 | 11 | NR | NR | 27 | 26 |
| Wang Q | 2024 | 9 (8.3%) | 0 (0) | 23 (20.8%) | 9 (5.7) | 43 (39.4) | 70 (44.3) | 76 (69.7) | 121 (76.6) |
| Chen | 2023 | NR | NR | NR | NR | 65 (16.54%) | 60 (11.76%) | 226 (57.51%) | 288 (56.47%) |
| Chen | 2025 | 8 (6.56%) | 7 (4.22%) | 12 (9.84%) | 14 (8.43%) | 34 (27.87%) | 46 (27.71%) | 73 (59.84%) | 102 (61.45%) |
| Dell'Era | 2024 | NR | NR | NR | NR | 7 (35%) | 7 (39%) | 19 (95%) | 18 (100%) |
| Kono | 2025 | NR | NR | NR | NR | 10 (13.3%) | 95 (32.1%) | 50 (66.7%) | 191 (64.5%) |
| Lee-Ky | 2025 | 33 (13.6%) | 32 (6.5%) | 56 (23.0%) | 136 (27.5%) | Paroxysmal AF 24 (9.9%), Persistent AF 24 (9.9%) | Paroxysmal AF 99 (20.0%), Persistent AF 76 (15.4%) | 118 (48.6%) | 259 (52.3%) |
| Mao | 2024 | NR | NR | 13 (42%) | 15 (52%) | 5 (16%) | 10 (35%) | 17 (55%) | 16 (55%) |
| Mao Y | 2023 | NR | NR | NR | NR | 17 (37.8%) | 13 (39.4%) | 35 (77.8%) | 28 (84.8%) |
| Maqueda | 2024 | 11 (11%) | 18 (18%) | 44 (44%) | 46 (46%) | 3 (3%) | 5 (5%) | 62 (62%) | 68 (68%) |
| Zhang S. | 2021 | NR | NR | NR | NR | 4 (13.80%) | 3 (8.11%) | 17 (58.62%) | 16 (43.24%) |
| Zhu H. | 2021 | 43 (10.5%) | 1 (0.3%) | 95 (23.4%) | 14 (4.5%) | 178 (43.8%) | 129 (41.2%) | 244 (60.1%) | 200 (63.9%) |
| Zhu H. | 2023 | 33 (12.8%) | 4 (1.5%) | 65 (25.5%) | 16 (6.5%) | NR | NR | 142 (55.3%) | 164 (60.7%) |
| Miyajima et al. | 2022 | NR | NR | NR | NR | 11 | 20 | 22 | 32 |
| Niu H.X.et al. | 2021 | 4 | 7 | 7 | 12 | NR | NR | NR | NR |
| Riano Ondiviela et al. | 2021 | 15 | 13 | NR | NR | NR | NR | NR | NR |
| Sharma et al. | 2022 | NR | NR | NR | NR | 148 | 145 | 280 | 330 |
| Okubo et al | 2022 | 8 | 11 | 10 | 8 | 13 | 16 | 33 | 33 |
| Sun Z | 2020 | 1 | 2 | 2 | 3 | 2 | 0 | 4 | 4 |
| Wang J.F. et al. | 2019 | NR | NR | NR | NR | 14 | 12 | 34 | 37 |
| Wang Z et al | 2021 | 3 | 2 | 3 | 6 | NR | NR | 34 | 25 |
| Yao L. et al. | 2022 | NR | NR | NR | NR | NR | NR | NR | NR |
| Zhang J. M. | 2019 | NR | NR | NR | NR | 5 | 2 | 11 | 9 |

| **Baseline characteristics table of all the included studies with demographics and clinical characteristics. (continued)** | | | | | | | | | |
| --- | --- | --- | --- | --- | --- | --- | --- | --- | --- |
| **Author** | **Year of Publication** | **Comorbidities n(%)** | | | |  |  |  |  |
|  |  | **DM n(%)** | | **CAD n(%)** | |  |  |  |  |
|  |  | **LBBP** | **RVP** | **LBBP** | **RVP** |  |  |  |  |
| Wang X | 2024 | 29 (24%) | 24 (21%) | 45 (38%) | 52 (44%) |  |  |  |  |
| Zhao | 2023 | 9 (21.43%) | 7 (16.67%) | NR | NR |  |  |  |  |
| Zhang | 2024 | 13 (30.2) | 9 (20.9) | 11 (25.6) | 7 (16.3) |  |  |  |  |
| Yao | 2025 | 11 (36.7) | 6 (20.0) | 2 (6.7) | 3 (10.0) |  |  |  |  |
| Liu X. et al | 2022 | 6 | 2 | 11 | 3 |  |  |  |  |
| Li Q. et al | 2021 | 9 | 3 | NR | NR |  |  |  |  |
| Liu et al. | 2022 | 7 | 4 | 12 | 5 |  |  |  |  |
| Li X. et al | 2021 | 50 | 25 | 31 | 20 |  |  |  |  |
| Li W | 2022 | 11 | 5 | 3 | 2 |  |  |  |  |
| Das. et al | 2020 | NR | NR | NR | NR |  |  |  |  |
| Chen X. et al | 2020 | 35 | 38 | 32 | 46 |  |  |  |  |
| Chen K. et al | 2022 | NR | NR | NR | NR |  |  |  |  |
| Chen K. et al | 2018 | NR | NR | 5 | 10 |  |  |  |  |
| Cai B. et al | 2020 | 10 | 4 | 8 | 2 |  |  |  |  |
| Byeon K | 2022 | 15 | 30 | NR | NR |  |  |  |  |
| Okubo 2025 | 2025 | 20 (24.7%) | 19 (24.1%) | 7 (8.6%) | 8 (10.1%) |  |  |  |  |
| Palmisano | 2023 | 13 | NR | 16 | NR |  |  |  |  |
| Ramalingam et al | 2024 | 21 | 17 | 22 | 21 |  |  |  |  |
| Wang Q | 2024 | 30 (27.5) | 41 (25.9) | 37 (33.9) | 50 (31.6) |  |  |  |  |
| Chen | 2023 | 91 (23.16%) | 128 (25.10%) | 75 (19.08%) | 92 (18.04%) |  |  |  |  |
| Chen | 2025 | 20 (16.39%) | 29 (17.47%) | 18 (14.75%) | 23 (13.86%) |  |  |  |  |
| Dell'Era | 2024 | 10 (50%) | 4 (22%) | 8 (40%) | 2 (11%) |  |  |  |  |
| Kono | 2025 | 23 (30.7%) | 73 (24.7%) | NR | NR |  |  |  |  |
| Lee-Ky | 2025 | 71 (29.2%) | 112 (22.6%) | 42 (17.3%) | 78 (15.8%) |  |  |  |  |
| Mao | 2024 | 6 (19%) | 9 (31%) | 6 (19%) | 7 (24%) |  |  |  |  |
| Mao Y | 2023 | 17 (37.8%) | 9 (27.3%) | 17 (37.8%) | 10 (30.3%) |  |  |  |  |
| Maqueda | 2024 | 53 (53%) | 49 (49%) | NR | NR |  |  |  |  |
| Zhang S. | 2021 | 9 (31.03%) | 6 (16.22%) | 7 (24.14%) | 12 (32.43%) |  |  |  |  |
| Zhu H. | 2021 | 79 (19.5%) | 72 (23.0%) | 76 (18.7%) | 66 (21.1%) |  |  |  |  |
| Zhu H. | 2023 | 39 (15.2%) | 60 (22.3%) | 48 (18.7%) | 59 (21.9%) |  |  |  |  |
| Miyajima et al. | 2022 | 10 | 11 | 8 | 9 |  |  |  |  |
| Niu H.X.et al. | 2021 | NR | NR | NR | NR |  |  |  |  |
| Riano Ondiviela et al. | 2021 | NR | NR | NR | NR |  |  |  |  |
| Sharma et al. | 2022 | 114 | 130 | 163 | 173 |  |  |  |  |
| Okubo et al | 2022 | 10 | 14 | NR | NR |  |  |  |  |
| Sun Z | 2020 | 7 | 4 | 1 | 2 |  |  |  |  |
| Wang J.F. et al. | 2019 | 11 | 15 | 10 | 12 |  |  |  |  |
| Wang Z et al | 2021 | 7 | 10 | 10 | 11 |  |  |  |  |
| Yao L. et al. | 2022 | NR | NR | NR | NR |  |  |  |  |
| Zhang J. M. | 2019 | 6 | 1 | 4 | 6 |  |  |  |  |
